# Bioactive Compounds with Antiglioma Activity from Marine Species

**DOI:** 10.3390/biomedicines9080886

**Published:** 2021-07-25

**Authors:** Rodion Khotimchenko, Igor Bryukhovetskiy, Maksim Khotimchenko, Yuri Khotimchenko

**Affiliations:** 1 School of Biomedicine, Far Eastern Federal University, 690090 Vladivostok, Russia; khotimchenko.ry@dvfu.ru (R.K.); bryukhovetskiy.is@dvfu.ru (I.B.); khotimchenko.my@dvfu.ru (M.K.); 2 Laboratory of Pharmacology, A. V. Zhirmunsky National Center of Marine Biology, Far Eastern Branch, Russian Academy of Sciences, 690950 Vladivostok, Russia

**Keywords:** antitumor activity, natural compounds, glioma multiforme, brain tumors, marine species

## Abstract

The search for new chemical compounds with antitumor pharmacological activity is a necessary process for creating more effective drugs for each specific malignancy type. This review presents the outcomes of screening studies of natural compounds with high anti-glioma activity. Despite significant advances in cancer therapy, there are still some tumors currently considered completely incurable including brain gliomas. This review covers the main problems of the glioma chemotherapy including drug resistance, side effects of common anti-glioma drugs, and genetic diversity of brain tumors. The main emphasis is made on the characterization of natural compounds isolated from marine organisms because taxonomic diversity of organisms in seawaters significantly exceeds that of terrestrial species. Thus, we should expect greater chemical diversity of marine compounds and greater likelihood of finding effective molecules with antiglioma activity. The review covers at least 15 classes of organic compounds with their chemical formulas provided as well as semi-inhibitory concentrations, mechanisms of action, and pharmacokinetic profiles. In conclusion, the analysis of the taxonomic diversity of marine species containing bioactives with antiglioma activity is performed noting cytotoxicity indicators and to the tumor cells in comparison with similar indicators of antitumor agents approved for clinical use as antiglioblastoma chemotherapeutics.

## 1. Introduction

### 1.1. Brain Tumors: Main Limitations of Therapeutics 

Despite substantial success achieved in the therapy of malignancies within the recent years, there are a few tumors even now considered absolutely incurable. Among them, the most malignant ones with the fulminant progression belong to a group of brain tumors generally termed gliomas that were shown to be barely sensitive to chemotherapy. Gliomas are a group of specific brain tumors originated from the glial (or non-neuronal) cells in the central nervous system (CNS). The term ‘glioma’ covers a large and diverse group of intrinsic tumors with a common classification based on typical microscopic patterns of the originated putative cells along the glial precursor cell lineages [1,2,3].

The standard therapeutic strategy of the GBM treatment includes surgical resection with the following radiotherapy and chemotherapy with temozolomide (TMZ). Based on the report issued by the Central Brain Tumor Registry of the United States (CBTRUS), one- and five-year survival rates were 40.2% and 5.6% from 2000 to 2015, respectively [4]. Despite recent findings published by the National Cancer Database (NCDB) showing significant improvement in the three-year survival rates for glioblastoma multiforme from 8.0% to 10.5% (*p* < 0.01) in 2004–2013 [5], an overall life prognosis remains poor, indicating that contemporary therapeutic approaches for GBM are not effective. Although chemotherapy is considered as essential part of the treatment and prevention of malignancies, just a few drugs have been approved for the treatment of gliomas including TMZ, carmustine, lomustine, and bevacizumab [6]. Therefore, obvious unMet medical need still exists requiring discovery of the lead compounds with significant anti-glioma activity.

The biggest problem of the malignant tumor chemotherapy is their multidrug resistance. It involves several pathways responsible for development of the tumor cell tolerance to the methylating agents such as increased O-6-methylguanine-DNA methyltransferase (MGMT) expression, impairment of the post-replication mismatch repair system (MMR), or the base excision repair (BER) pathways induced by genetic and epigenetic changes, and resistance to apoptosis due to the reduced Bax (pro-apoptotic protein) level and increased Bcl-2 (anti-apoptotic protein) content [7,8].

BER is the main pathway involved in elimination and/or repair of the oxidized, alkylated, and mismatched bases, apurinic/apyrimidinic (AP) sites, and DNA single strand breaks produced by reactive oxygen species (ROS), ionizing radiation, and alkylating agents [9,10].

Apoptosis is a caspase-dependent programmed cell death usually manifested by distinctive morphological and biochemical hallmarks, such as membrane blebbing, cell shrinkage, nuclear condensation and fragmentation, mitochondrial fragmentation, and caspases activation [11]. Apoptosis reaction is a result of either death receptor-independent (intrinsic or mitochondrial) or dependent (extrinsic) pathways [12]. One of the main mechanisms of the cancer drug resistance is an apoptosis evasion that can be realized through the reduced caspase function, impaired death receptor signaling, and induced misbalanced between pro-apoptotic and anti-apoptotic proteins. Autophagy is considered a universal process that can provide both cell death and cell survival effects depending on the cellular content. The cell death mechanism is commonly activated when apoptotic mechanisms are suppressed as it works as a reverse mechanism of the cancer death eradication. In some studies, autophagy was described as a pro-survival response that possibly contributes to the development of resistance to other anticancer drugs in glioma cells.

Taking to account the abovementioned mechanisms of the drug resistance development, the following ways leading to increased efficiency of the anti-glioblastoma chemotherapy may be suggested including MGMT level modulation and inhibition of the BER pathway via poly(ADP-ribose) (PARP) inhibition, which is known to take part in detecting and signaling the DNA damage generated by methylating agents. Other approaches include blockage of the APE endonuclease activity; modulation of the expression and activity of the regulatory elements responsible for the proper cell functions and apoptosis such as the Bcl-2 protein family, p53 protein, inhibitor of apoptosis proteins or the receptor tyrosine kinases (e.g., EGFR); inhibition of molecular targets related to apoptosis and autophagy resistance pathways, such as the mammalian target of rapamycin (mTOR), phosphatidylinositol 3-kinase (PI3K), and protein kinase B (PKB or Akt) pathway.

Another serious problem of the chemotherapy against glioblastomas is related to severe adverse effects induced by the use of alkylating agents. They include hematotoxicity, myelosuppression, hepatotoxicity, cerebral edema, interference in normal blood flow, blood clots, coronary heart disease attacks and peripheral artery disorders, gastrointestinal lesions and bleeding, as well as allergic reactions [13]. All these substantial limitations of the up-to-date methods of chemotherapy require development of the novel pharmaceuticals with high efficiency and low toxicity with reduced risk of adverse effects providing favorable forecast of the disease outcome.

Discovery of the new chemical compounds exerting pharmacological effects, in particular, showing anti-glioma effects is a sequential process of building new pharmaceuticals, which should be more effective towards each specific tumor type. Due to the enormous diversity of the tumor genotype, antitumor therapy based on the use of only one drug is less effective for elimination of the tumor cells in comparison with a joint administration of two and more pharmaceuticals. Such combinations are in theory more promising approach for eradication of the high tumor cell number the use of monotherapy [14]. The new approaches discovering combinations of multiple inhibitors have been proposed along with the identification of key driver mutations that are specific to each patient.

### 1.2. Molecular Targets for the Anti-Glioma Drug Discovery

Conventional antitumor compounds may be divided into cytostatics and cytotoxic agents. Their majority were selected through empirical screening of synthetic and natural compounds. Character of their damaging influence and nature of the targets being affected have been studied after determination of their antitumor potency. At the same time, within the recent two decades the discovery of the new potential antitumor compounds has demonstrated a tendency of using rational drug design synthesis of compounds with their properties predicted beforehand. Their influence on the tumor tissues is basically limited by specific molecular targets, which presence in the cancer cells is mandatory for administration of such agents. Antitumor drugs created via such approach are called ‘targeted drugs’. In contrast to conventional cytostatics and cytotoxins, they affect molecular targets performing intracellular transduction of the ‘death’ and ‘survival’ signals and playing important role in the cancer cell fate. Determination of the molecular targets make a ground for the high-throughput screening accelerating new drug discovery process and preclinical studies. On the other hand, it helps develop more effective combinatorial treatment regimens specifically focused on the problems of differential sensitivity and therapeutic resistance. Molecular characteristics of GBM have shown a few goal mutations regulating gliomagenesis. This approach makes a base of personalized influence on GBM, which is considered highly promising. Despite the lack of the full understanding of all GBM key mutations and absence of agents targeting some known key drivers, significant progress in this field was achieved within recent years.

Molecular markers may include telomerase reverse transcriptase (TERT) promoter mutation, amplification of the epidermal growth factor receptor (EGFR) gene, phosphatase and tension homolog (PTEN) tumor suppressor gene mutation as well as tumor protein (TP53) and isocitrate dehydrogenase (IDH) 1/2 mutations [15]. EGFR amplification is often associated with high expression of EGFRvIII, a ligand-independent constitutively active mutant of EGFR, capable of persistently activating PI3K/v-Akt murine thymoma viral oncogene homolog (Akt) signaling pathway that promotes the survival of the glioma cells. The genetic mutations of PTEN down-regulate the Akt signaling pathway. The secondary GBM, that is usually associated with TP53 mutations, consistently exhibits the genetic mutation of (IDH1) down regulating the hypoxia-inducible factor 1-alpha (HIFA). A series of oncogenic pathways in GBM including the p53, the retinoblastoma tumor suppressor (RB), and the receptor tyrosine kinases/RAS/phosphatidylinositol 3 kinase (RTK/RAS/PI3K) pathways are activated in majority of cases [16]. These genetic alterations promote cell proliferation and enhance cell survival capacity, thus making tumor cells to escape from cell-cycle checkpoints, senescence, and apoptosis. Beyond genetic abnormalities, epigenetic alterations such as hypermethylation of the pro-apoptotic genes and tumor suppressor genes, and hypomethylation of genes that are normally silenced such as matrix metalloprotease (MMP9) gene (related with invasion) has been described in GBM [17]. Current drugs specifically targeting EGFR tyrosine kinase activity or selectively inhibiting mTOR, a PI3K/Akt downstream signal transducer showed little efficacy in treatment of primary glioblastoma [18]. Probably, the most promising approach for suppression of the glioblastoma progression involves concurrent inhibition of the multiple signaling pathways affecting molecular targets that play pivotal role in the survival and progression of tumor cells.

Cell cycle abnormalities in many human cancer types are generally often caused by hyperactivation of cyclin-dependent kinases (CDKs) belonging to the serine/threonine-specific protein kinase family. Abnormal regulation of the CDK4- and CDK6-cyclin D-INK4-retinoblastoma protein (Rb) signaling pathway is one of the most common aberrations noted in many human cancers, in particular, in GBMs, which cells show excessive CDK4/CDK6 activation [19]. Therefore, CDKs can be considered as targets for potential antitumor agents.

The new findings were published within the recent years indicating the aberrant activation of the specific molecular pathways that play crucial roles in progression, recurrence, and invasion of the glioma tumors. A group of molecular signals suggested to be important for glioma cells includes PI3K/AKT, mitogen-activated protein kinase (MAPK), and Wingless (WNT)/b-catenin pathways. PI3K/AKT pathway was shown to be activated in glioma and this activation of such signaling is essential for survival, proliferation, invasion of cells and oncogenesis in glioma cells [20]. Akt is phosphorylated following the PI3K activation. This phosphorylated Akt promotes cell survival and proliferation through phosphorylating downstream targets including anti-apoptotic protein Bad, transcriptional factors forkhead box protein (FOXO) and NF-kB. Moreover, PI3K/AKT signaling is essential for the glioma tumor growth and it increases glioma cell resistance to apoptosis induced by various stimuli. PTEN deletion or mutation is commonly noted lesion in various cancer types leading to activation of the PI3K/AKT pathway. PI3K/AKT pathway targeting has also been shown to be an effective way providing negative influence on glioma [20]. Therefore, suppressed activation of the PI3K/AKT pathway is thought to be one of the mechanisms inhibiting glioma growth. A protein chaperon Hsp90 providing correct folding, stability, and activation of its client proteins maintains stability and normal functioning of various signal proteins and plays a role in cell proliferation, growth, and survival. One of the serine-threonine kinase is Akt, whichis also referred as protein kinase B and shown to be closely associated with the growth factor-induced signaling pathway. Akt stimulates the growth factors and cytokines and is also recruited from cytosol to plasma membrane and then phosphorylated at two key regulatory sites, Thr308 and Ser473, by 3-phosphoinositide-dependent protein kinase-1 (PDK1). Akt generally binds to the HSP90 protein, which is required for the proper function of Akt as they form a chaperone–substrate protein complex. Reduction of the mutual Hsp90/Akt binding usually results in the Akt inactivation. Inhibition of the Hsp90/AKT complex formation with the following suppression of the PI3K/AKT signaling pathway can be considered as a promising way for therapeutic inhibition of glioma growth rate [21].

Checkpoint kinase 1 (Chk1) is another promising target for glioma therapy. Chk1 belongs to a group of Ser/Thr protein kinases involved into the pathways controlling the G2/M phase of the cell cycle. Increased expression of the Chk1 is typical for major part of the cancer types due to oncogenic activation and constant replicative stress. Inactivation of the Chk1 is a promising mechanism for the anticancer therapy because it contributes to genomic instability, chromosome catastrophe, and cancer cell death. Cdc25C is one of the well-defined targets for Chk1 regulation. Chk1 phosphorylates Cdc25C phosphatase at the Ser216 site promoting Cdc25C binding to the molecular chaperone 14-3-3 protein. The 14-3-3 protein then sequesters Cdc25C in the cytoplasm and may lead to the G_2_/M arrest of malignant human glioma cells [22]. Chk1 along with ataxia-telangiectasia-mutated-and-Rad3-related kinase (ATR) are two key components of the replication stress response. ATR/CHK1 signaling pathway prevents the entry of cells harboring damaged or incompletely replicated DNA into mitosis when the cells are exposed to DNA damaging agents. These properties of CHK1 and ATR make them promising target structures for discovery of the new anticancer drugs [23,24], and the Chk1 inhibitors should be considered as potential antitumor agents inducing increased DNA damage.

Tumor glycolytic enzymes hexokinase 2 (HK2), 6-phosphofructo-2-kinase/fructose-2,6-bisphosphatase (PFKFB3), pyruvate kinase M2 (PKM2), and lactate dehydrogenase 5 (LDH5) demonstrate increased activity in the glioma cells and supposed to be preferentially used by cancer cells. High glycolysis level was confirmed to be necessary for the fast and unlimited cell proliferation in tumors and may serve as marked sign of the glioma metabolism [25,26,27,28]. These enzymes can be also considered promising targets for potential anti-cancer agents. 

### 1.3. Marine Compounds as a Source of the New Anti-Glioma Agents

One of the sources for the prototypes of the new medicines with antitumor activity is a group of natural products (NPs), in particular, NPs of marine origin. Approximately 80% of the approved chemotherapeutic drugs, and more than 50% of all drugs used in clinical practice are thought to be based on the bioactive NPs [29]. In the numerous drug discovery studies, NPs have been a prime source of pharmacologically active agents purposed for the treatment of many cancer forms offering a promising opportunity for evaluation of new chemical classes of anticancer drugs [30].

Macrotaxonomical diversity of marine species is substantially higher than biodiversity of the terrestrial life forms. Among 35 animal phyla that are taxonomically identified today, 34 are found in the marine environMent, and many of them inhabit only aqueous media [31]. During the much longer evolution of marine species that lasted for 100 million years, harsh oceanic conditions—such as wide temperature range, salinity, great pressure, as well as a risk of predator attacks—contributed to the production of a high variety of molecules bearing unique properties in terms of diversity and structural and functional features. At present, many marine species including bacteria, cyanobacteria, fungi, microalgae, seaweeds, marine sponges, soft corals, sea fans, sea hares, nudibranchs, bryozoans, tunicates, sea cucumbers, and starfishes have been investigated for the presence of compounds with anti-cancer activities [32,33]. Despite the material supply and availability poses a great problem for the drug development based on marine products, which are usually obtained from natural sources in very tiny amounts, modern methods of the total chemical synthesis or semi-synthesis provide a reasonable solution. Biotechnological technologies are supposed to be more time and resource consuming and can be implemented for such purposes in a form of large-scale fermentation of the producer microorganisms or the cultivation of marine invertebrates [34]. Nowadays, numerous marine-based drugs initially obtained from NPs including spongian nucleoside cytarabine and spongian macrolide eribulin mesylate, brentuximab vedotin (an antibody-drug conjugate brentuximab with the monomethyl auristatin E (MMAE), which is a synthetic analog of dolastatin-10, produced by marine cyanobacteria) and second antibody-drug conjugate, polatuzumab vedotinconsisting of a CD76b-targeting antibody and MMAE, ascidian alkaloid trabectidine (ET-743) and ascidian depsipeptide plitidepsin is in use in the treatment of lymphocytic leukemia, anaplastic large T-cell systemic malignant lymphomas and B-cell lymphomas, Hodgkin’s and non-Hodgkin lymphomas, multiple myeloma, metastatic breast cancer, soft tissue sarcoma, and ovarian cancer [35].

The main goal of the present study was focused on the diversity of molecular structures or, speaking professional language, pharmacophore structures that are capable to inhibit development of human brain tumors and assess their effective concentrations against malignant and normal cells. Taken together, these data will allow us to outline a strategy for further research of the new anti-glioma drugs development and methods of personalized therapy for brain tumors. There are compounds with anti-glioma activity belonging to the various classes of chemical substances was discovered. Pharmacological properties of these substances will be discussed in the following chapters on the present review article.

## 2. Alkaloids

Alkaloids are cyclic compounds containing one or more nitrogen atoms in their cycle or side chain that demonstrate weak alkali properties. Main natural sources of alkaloids belong to the plant kingdom, and mainly to higher plants. However, within the recent years the number of alkaloid molecules found in the marine sources is gradually growing [36]. Those species include marine bacteria including cyanobacteria (or blue-green algae), actinomycetes, marine-derived fungi, marine algae, bryozoans, starfishes, holothurians, ascidians, and marine sponges [37]. Bioactive alkaloids as well as officinal medicine alkaloids exert various pharmacological effects including antitumor properties [38]. Alkaloids compounds demonstrating antiglioma activity are listed below with anticancer mechanisms given in details.

### 2.1. Imidazolone and Indole Alkaloids

A new alkaloid named zorrimidazolone isolated from the Mediterranean stolidobranch ascidian *Polyandrocarpa zorritensis* was shown to exert cytotoxic effects against C6 cells resulting in 60% decreased viability when applied in concentration 250 μM for 48 h under in vitro conditions. IC_50_ for zorrimidazolone providing inhibiting effects against C6 cells was within the micromolar range (Table 1), which is generally considered as moderate cytotoxic activity [39]. This new metabolite belongs to the 2-aminoimidazolone class of marine metabolites, which have been predominantly isolated from *Axinella* and *Agelas* marine sponges and very rarely found in the ascidians. The compounds from this class isolated from ascidians are the N,N-dimethylaminoimidazolone found in *Dendrodoa grossularia* [40] and the polyandrocarpamines A and B found in a Fijian *Polyandrocarpa* sp. [41]. Two monoindole alkaloids, 3-indolylglyoxylic acid and its methyl ester that were also found in *Polyandrocarpa zorritensis,* demonstrated weaker cytotoxic activity toward the C6 cells, which was found selective and depending on the concentration. The structurally close compound, 4-hydroxy-3-methoxyphenylglyoxylic acid methyl ester, which does not belong to the alkaloid class, did not affect C6 cell viability. According to the author conclusion, zorrimidazolone may be of interest for the development of potential antiproliferative molecules against gliomas.

**Figure 1:**
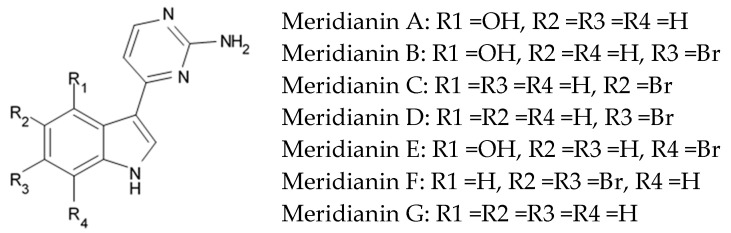
Chemical structure of meridianins A–G [44].

Indol alkaloids meridianins isolated from ascidian *Aplidium meridianum* [42] and variolin B found in Antarctic sponge *Kirkpatrickia varialosa* [43] became prototypes of the novel synthetic derivatives as well as hybrid molecules meriolins obtained from those compounds. The natural meridianins A, B, C, D, E, F, and G are brominated and/or hydroxylated 3-(2-aminopyrimidine)-indoles differing in the bromine and/or hydroxyl substitution (Figure 1) [44,45]. Meridianins were shown to inhibit various protein kinases, such as CDKs, glycogen synthase kinase-3, cyclic nucleotide-dependent kinases, and casein kinase 1 [46] playing important role in the cancer cell lifecycle. They also prevent cell proliferation and induce apoptosis due to their ability to penetrate cellular membrane and disturb activity of kinases responsible for cell division and death. Meridianin E was experimentally proved an effective protein kinase inhibitor with high selectivity regarding CDK1 and CDK5 [46]. One of the meridianin C derivatives substituted at the C-5 position was found to be strong and selective inhibitor of pim kinases (including pim-1, pim-2, and pim-3 overexpressed in various cancer cell types) with IC_50_ values within a nanomolar concentration range [47].

**Figure 2:**
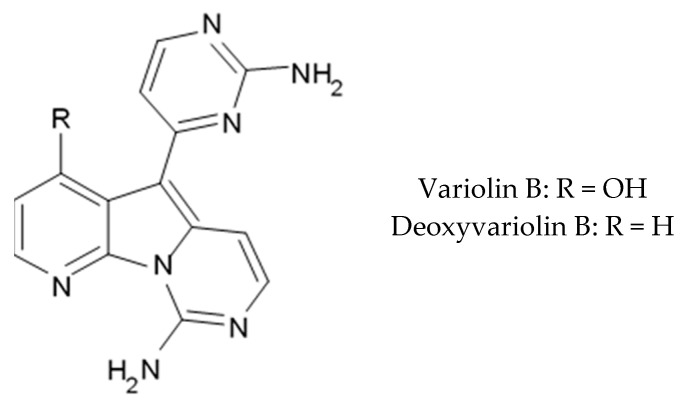
Chemical structure of variolin B and deoxyvariolin B.

Variolins (A, B, deoxyvariolin B, and N(3′)-methyl tetrahydrovariolin B) are natural marine alkaloids possessing an uncommon pyrido[3′,2′:4,5]pyrrolo[1,2-c]pyrimidine skeleton (Figure 2) [43]. Variolin B exerted anticancer activity on the P388 murine leukemia cell line with an IC_50_ value 716 nM. Variolin A and N(3′)-methyl tetrahydrovariolin B displayed significantly weaker activity against these cancer cells. Variolin B had been shown to induce apoptosis exerting highly potent cytotoxic activity against various human cancer cell lines including the ones with overexpressed level of cell efflux pump p-glycoprotein (PGP). Variolin B and deoxyvariolin B were experimentally confirmed to exert strong cytotoxic activity against various cancer cell lines with IC_50_ values varying from 50 to 100 nM. Both compounds were noted to inhibit the histone H1 phosphorylation mediated by cyclin E-CDK2, cyclin A-CDK2, cyclin B-CDK1, cyclin H-CDK7, and cyclin D-CDK4, with IC_50_ values in the micromolar range [48].

A new CDK inhibitory scaffold with antitumor activity has been composed by combining the common features of meridianins and variolins. Thus, the new class of synthetic 7-azaindole-containing analogues have been designed and the term ‘meriolin’ has been coined to describe this hybrid structure [49,50]. Meriolins exhibit better antiproliferative and proapoptotic properties in cell cultures than their ‘inspirational parent’ molecules. Meriolins was shown to exert significant inhibiting effects against CDKs and possess antiproliferative and pro-apoptotic activity in human cancer cell lines in vitro [50]. In particular, phosphorylation at CDK1-, CDK4-, and CDK9-specific sites has been shown to be counteracted by meriolins in neuroblastoma SH-SY5Y [49].

Nineteen different meriolin structures have been investigated in such human glioma cell lines as anaplastic astrocytoma SW1088 and glioblastoma U87. All mentioned meriolins efficiently decelerated tumor cell growth rate within IC_50_ range from 1 nM to 1 μM. Meriolin types 3, 5, and 15 provoked proliferation rate inhibition in both glioma cell lines. IC_50_ for SW1088 cells were 34, 32, and 46 nM, respectively, whereas these values for U87 were 76, 18.4, and 5.1 nM, respectively (Table 2). Meriolin types 5 and 15 showed the strongest antiproliferative activity via induction of the cell cycle block and apoptosis in glioma cells [51].

In the in vivo experiments, meriolin 15 inhibited proliferation of the glioma cells, induced apoptosis, and reduced the number of non-differentiated tumor cells in the U87 glioblastoma xenograft model in nude mice. The results suggested that meriolin 15 inhibits DK7/CDK9 consequently decreasing RNA polymerase II and its phosphorylation that results in downregulation of the survival factor Mcl-1, thereby allowing the activation of proapoptotic factors (Noxa, Bim, etc.) [51]. The cytotoxic effect of meriolins have been also investigated in the rat primary proliferating astrocyte and neuron cultures. Meriolin type 5 and 15 demonstrated antiproliferative and pro-death activities. IC_50_ of the meriolin 15 is 7.8 nM in astrocytes and 4.7 nM in neurons. Therefore, meriolins provide extremely potent antiglioma activity and at the same time exert strong toxic effect in healthy astrocytes. This requires further investigations focused on development of other derivative with less toxicity regarding normal healthy cells.

### 2.2. Fascaplysins

**Figure 3:**
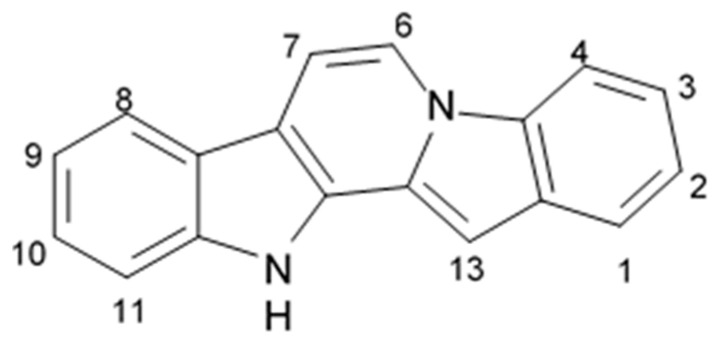
Structure of 12H-pyrido[1,2-a:3,4-b′]diindole.

Fascaplysin is a red pigment and bis-indole alkaloid (12, 13-Dihydro-13-oxopyrido[1,2-a:3,4-b′] diindol-5-ium chloride) that was initially isolated from the Fijian sponge *Fascapfysinopsis* sp. in 1988 and characterized as the novel 12H-pyrido[1,2-a:3,4-b′]diindole ring system (Figure 3). It was considered a unique compound among the natural products [52]. Later fascaplysin and some related compounds homofascaplysin A and 3-bromohomofascaplysin A were discovered and isolated from ascidian *Didemnum* sp. [53]. Then synthesis of fascaplysin and its derivatives was elaborated in a relatively short period, and this process is still ongoing [54,55].

Fascaplysin exerts various biological activities including selective kinase 4 (CDK-4) inhibition, DNA binding, and antiangiogenic effects [56]. Fascaplysin was found to exert cytotoxic effects in a panel with at least 36 cancer cell lines with IC_50_ in a range 0.6–4 μM [54,57,58,59]. It was also shown that fascaplysin and its derivatives are quite efficacious in in vivo mouse tumor models against human colon carcinoma HCT-116, human non-small-cell lung carcinoma NCI-H460 [54], human malignant melanoma A375 [58], and murine sarcoma S180 via induction of apoptosis and antiangiogenesis [57].

The molecular mechanism of fascaplysin-induced apoptosis is directly linked to activation of the caspase-3, caspase-8, and caspase-9 pathways, cleavage of Bid, release of cytochrome C into cytosol and downregulation of the Bcl-2 level in cancer cells. Fascaplysin was also shown to block vascular endothelial growth factor (VEGF), inhibit proliferation, and induce apoptosis of human umbilical vein endothelial cells (HUVECs) [56]. TNF and TNF receptor superfamily in HUVECs and hepatocarcinoma cells BeL-7402 cab be regulated by fascaplysin resulting in the tumor necrosis-related apoptosis-inducing ligand-(TRAIL)-induced apoptosis leading to activation of caspases 3 and 9 and Bid decrease [60]. Fascaplysin was noted to induce high cytotoxicity against small-cell lung cancer (SCLC) cells resulting in the cell cycle arrest in G_1_/G_0_ at lower concentration of active compounds and in S-phase at the higher fascaplysin level. Its high cytotoxic activity against these cancer cells is due to multiple routes of action, affecting topoisomerase I, integrity of DNA and generation of reactive oxygen species (ROS) [57].

Anticancer activity of fascaplysin under in vitro conditions results in reduced expression of CDK4, cyclin D1 and downregulation of the CDK4-specific Ser795 retinoblastoma protein (Rb) phosphorylation in HeLa cell line. Apoptosis induced by fascaplysin is related to activation of effector caspases, migration of cytochrome C into cytosol, and reduced Bcl-2 expression. Cytotoxic effects of fascaplysin were observed in chemosensitive promyelocytic HL-60 cancer cells as it activates both pro-apoptotic events like PARP-1 cleavage/caspase activation and triggered autophagy as shown by the increased expression of LC3-II, ATG7, and beclin [61]. It should be emphasized that fascaplysin demonstrates significant anticancer activity in non-small cell lung cancer (NSCLC) and small cell lung cancer (SCLC) lines, which is not depending on the CDK4 pathway suggesting the direct effects on the DNA function and transcription of various proteins [59]. Another mechanism of antitumor activity was found to be related in increase of the phosphorylation of Akt/PKB and adenosine monophosphate-activated protein kinase (AMPK) that play a key role in anti-apoptotic or pro-survival pathways in cancer [58]. Fascaplysin in addition was shown to abolish phosphorylation of mTOR, 4EBP1, and p70S6K1 thus triggering the cap-dependent translation machinery and affecting expression of oncoproteins such as survivin, c-myc, cyclin D1, VEGF, and HIF-1α. Alkaloid derivative 7-chloro-fascaplysin similarly inhibits cell survival through interference with the PI3K/Akt/mTOR pathway, which in turn modulates HIF-1α, eNOS, and MMP-2/9 in the breast cancer cell line [62]. Experimental treatment of the HCT116 (colorectal), A375 (malignant melanoma), and H1975 (lung) xenografted tumor tissues resulted in decreased tumor angiogenesis and increased cleaved-caspase-3. Consequently, survivin and HIF-1α are downregulated by suppressing 4EBP1-p70S6K1 axis-mediated de novo protein synthesis as was confirmed in the in vitro and in vivo experiments. In addition, fascaplysin inhibits vascular endothelial growth factor receptor 2 (VEGFR2) and tropomyosin-related kinase A (TRKA) via DFG-out non-competitive inhibition. These data suggest that fascaplysin inhibits TRKA and VEGFR2 and downregulates survivin and HIF-1α resulting in tumor growth inhibition [58].

In our laboratory, antitumor efficiency of fascaplysin and its synthetic derivatives such as 7-phenylfascaplysin, 3-chlorofascaplysin, 3-bromofascaplysin, and 10-bromofascaplysin in C6 glioma cells was compared under in vitro conditions. Fascaplysin was applied in concentration range from 0.5 to 2 µM and exerted significant dose- and time-dependent antiproliferative and cytotoxic effects with IC_50_ about 1.0 µM (Table 3). Inhibiting effects were noted to be associated with the dose-dependent increase of the glioma cell number being in apoptosis stage. Inhibiting effect induced by fascaplysin in that model was significantly higher than that of temozolomide [63]. Cytotoxic influence of all fascaplysin derivatives investigated in our study was superior to the activity of unsubstituted fascaplysin. In particular, 3-bromofascaplysin and 7-phenylfascaplysin had shown the highest capacity to induce C6 glioma cell death [64].

On the other hand, high cytotoxicity of fascaplysin regarding normal cells should be emphasized, because it may be associated with its planar structure contributing d-s DNA intercalation [65]. Development of the regulating approaches for such unusual cytotoxicity requires further investigation of various fascaplysin derivatives with the better safety profile.

### 2.3. Carboline Alkaloids (Tricyclic Pyridoindoles)

A large group of natural, semisynthetic, and synthetic compounds is presented with tricyclic pyridoindoles, i.e., carbolines, which are classified as α-carbolines (pyrido[2,3-b]indoles), β-carbolines (pyrido[3,4-b]indoles, γ-carbolines (pyrido[4,3-b]indoles) and δ-carbolines (pyrido[3,2-b]indoles) depending on the position of the pyridine nitrogen relative to the indole. β-carbolines initially discovered in *Peganum harmala* and later widely found in medicinal plants and natural herbal products are the well investigated compounds [66]. Alkaloids composed of tricyclic moiety and structurally relayed to both indole and carbazole compounds belong to the group of α-carbolines. Among the marine species, the representatives of the Tunicata subtype, mostly ascidians, are the source of α-carboline alkaloids [67]. Natural α-carbolines and their synthetic derivatives are of great interest as the lead compounds in the new drug development. α-Carboline related derivatives have been synthesized and experimentally demonstrated to show anticancer activities [68].

Six α-carboline analogues were synthesized, designated as TJY-13, TJY-14, TJY-16, TJY-18, TJY-22, TJY-24, and then tested on the human glioma cell lines U87, U251, T98G and rat glioma C6. 48 h incubation of the glioma cells with those compounds resulted in inhibited proliferation as shown in Table 4. α-Carboline analog TJY-16 (6-acetyl-9-(3,4,5-trimethoxybenzyl)-9H-pyrido[2,3-b]indole) was found to be the strongest and highly potent inhibitor of the glioma cell viability even when applied in the nanomole concentration [69]. 50 nM concentration of TJY-16 induced cell circle arrest in the G_2_/M phase in U87 and T98G glioma cells. Moreover, 24–48 treatment of the tumor cells with TJY-16 led to significantly greater portion of the sub-G_1_ phase blocked cells. Microscopic images of the glioma cells treated with TJY-16 demonstrated apoptotic signs, such as nuclear shrinkage and DNA condensation, and increased level of the cleaved caspase-3. Caspase-8 activation and depolarization of the mitochondrial membrane potential (ΔΨm) indicated that both extrinsic and intrinsic apoptotic pathways were involved in TJY-16-induced apoptosis. Surprisingly, cell death was noted in three human glioma cell lines, but it was not observed in the C6 rat glioma cell culture.

TJY-16 administered intraperitoneally once per day for 10 days in a dose 24 mg/kg in the nude mice with xenograft tumor model of U87 glioma cells effectively inhibited tumor growth and induced caspase-3 activation. Anti-glioma effect of TJY-16 was significantly higher than that of temozolomide, which was administered orally once per day for 5 days in a dose 80 mg/kg [69]. Based on these results, authors of the research study considered α-carboline derivative TJY-16 as a perspective agent for the therapy of malignant gliomas.

Tetrahydroisoquinoline alkaloids with antitumor activity isolated from marine species are commonly categorized into ecteinascidins and renieramycins. Ecteinascidin-743 (ET-743), which is also known as trabectedin, yondelis, and CID 108150 is the one of the most potent representatives of ecteinascidins, isolated from Carribean ascidian (subphylum *Tunicata*) *Ecteinascidia turbinata* [70]. ET-743 was approved as a first line drug for the treatment of inoperable soft tissue sarcoma with high resistance to conventional chemotherapeutics. Mechanism of antitumor activity of the ET-743 is related to its capacity of making complexes with minor groove of DNA double helix and alkylate N2 guanin, thus preventing cell proliferation, DNA reparation, and activation of transcription. This cascade leads in apoptosis of the target cells [71]. Three alkaloids namely ecteinascidin-770 (ET-770, a stabilized derivative of ET-743, isolated from ascidian *E. thurstoni*), 2′-N-4″-pyridinecarbonyl derivative of ET-770 and renieramycin M, a major bis-1,2,3,4-tetrahydroisoquinolinequinone alkaloid from the marine sponge *Xestospongia* sp. were tested in human glioblastoma cells U373MG. All tested compounds exerted strong anti-glioma influence at the nanomolar concentrations after a 72 h treatment (Table 5) [72].

Drug effects against U373MG cells exerted by each compound in IC_50_ concentration within 72 h resulted in induced PARP and CASP3 cleavage that reflect molecular markers of ongoing apoptosis. Investigation of the gene expression profile of the whole genome of the U373MG cells treated with alkaloids in IC_50_ concentrations within 24 h have shown that ecteinascidin-770 reduced expression of 426 genes and upregulated 45 genes, renieramycin M suppressed expression of 274 genes and increased expression of 9 genes, and 2′-N-4″-pyridinecarbonyl derivative of ET-770 downregulated 417 genes and upregulated 84 genes. Generally, upregulated genes significantly prevailed over the genes with reduced expression for each tested compound. It should be noted that a set of 196 downregulated genes and 6 upregulated genes has shown to be the same for all tested compounds suggesting the presence of joint pathway involved in induction of apoptosis.

Analysis of molecular network made possible identifying EGFR signal pathway in the U373MG cells as a supplementation to the axonal and cell adhesion as significant downregulating gene pathways. ErbB (EGFR) signal pathway was found to be composed of focal adhesion kinase (FAK)/PTK2, Akt3, and GSK3β acting as key molecules involved in cell migration and development of the nervous system. At the same time, a set of genes being upregulated by alkaloid have shown significant linkage via cell cycle with CDC25A working as a hub molecule. It was also shown that suppressed expression of Akt3 by RNA interference reduce expression of the Bad phosphorylated form resulting in induced caspase-dependent apoptosis in glioma cells [73]. Akt3 is known to be required for anchorage-independent growth of glioma cells. Glycosynthase kinase 3β (GSK3β) involved in apoptotic pathways presents a serine/threonine kinase that regulates Wnt/β-catenin named Hedgehog in an integrated manner and receptor tyrosine kinase (RTK) signaling pathways that play a key role in such cellular functions as glycogen metabolism, cell differentiation, proliferation, and apoptosis. Downregulating effects of siRNA towards GSK3β activity inhibit cell migration and induce apoptosis of glioma cells via c-Myc activation and suppression of the nuclear factor-κB (NF-κB) activity [74]. The cell cycle progression inhibiting and activating processes are known to be regulated by the complex checkpoint mechanism that includes cyclins A, B, D, and E along with cyclin dependent kinases (CDKs) and CDK inhibitors (CDKIs) of both the Cip/Kip and Ink4 families. The hypophosphorylated Rb protein interacts with the E2F family transcription factors E2F1, E2F2, and E2F3, and activates gene expression that is crucial for the cell cycle progression. Rb protein, hyperphosphorylated by cyclin D1-CDK4 and cyclin E1-CDK2 complexes, in turn releases E2Fs and represses the cell cycle gene expression [75]. Thus, Rb/E2F pathway acts a molecular switch contributing to either progressing or arrest of the cell cycle. The U373MG molecular network in the glioma cells composed of the integral set of downregulated and upregulated genes generally affected by all tested tetrahydroisoquinoline alkaloids was found to have a significant relation with transcription regulating mechanisms via transcription factors Rb/E2F [72]. These findings suggest those alkaloids work as the DNA-alkylating agents interfering cell division finally resulting in apoptosis of the target cells.

It is already known that the human glioblastoma cell lines MO59K and MO59J are basically characterized by the presence or absence of the DNA-dependent protein kinase (DNA-PK) catalytic subunit, respectively, which considered a part of the DNA double-strand-break repair pathway. These cells have been shown to have different responses to the treatment with ET-743. MO59J cells were much more sensitive to ET-743 treatment compared to the MO59K cells with 5-fold lower values of the ET-743 IC_50_ (0.041 ± 0.004 vs. 0.2 ± 0.02 nM) (*p* < 0.05) [76]. These results confirm that tetrahydroisoquinoline alkaloids possess a unique mechanism of interaction with DNA.

### 2.4. Pyrrole Alkaloids

**Figure 4:**
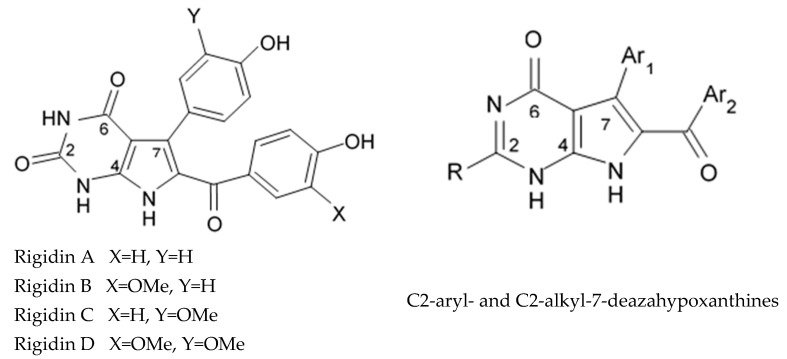
Structures of the marine alkaloid rigidins A, B, C, D, and their 7-deazahypoxanthine based synthetic analogues [79].

Alkaloids rigidins A, B, C, D, and E initially isolated from the tunicate *Eudistoma* cf. *rigida* [77,78] and later synthesized [79] were the most notable ones among the group of pyrrole alkaloids but have been shown to have low activity regarding cultivated human cancer cells. Further investigations of synthetic compounds led to development of the promising approaches to modification of the 7-deazaxanthine skeleton that typical of rigidins converting into corresponding 7-deazahypoxanthines (Figure 4).

Novel marine rigidin analogues C2-aryl- and C2-alkyl-7-deazahypoxanthines obtained via synthesis techniques were proposed for construction of the pyrrolo[2,3-d]pyrimidine ring system and applied in submicromolar and nanomolar quantities exerting strong anti-proliferating activity against different cell lines including multidrug resistant tumors such as glioblastoma, melanoma, and non-small cell lung cancer. As the only difference between 7-deazaxantine rigidin scaffold and 7-deazahypoxanthines skeleton structure is the lack of carbonyl group at C2 in the latter structure, such modifications in this position are supposed to be critical for their activity. It was demonstrated that one of the C2-methyl-7-deazahypoxanthines, namely 6-benzoyl-2-methyl-5-phenyl-1H-pyrrolo[2,3-d]pyrimidin-4(7H)-one, exerted the greatest antiproliferative activity against glioblastoma cell line U-87 (GI_50_ = 0.077 ± 0.002 μM), human cervical adenocarcinoma HeLa (GI_50_ = 0.029 ± 0.001 μM), breast adenocarcinoma MCF-7 (0.035 ± 0.003 μM), and lung carcinoma cell line A549 (0.25 ± 0.01 μM). Other synthetic C2-methyl-7-deazahypoxanthines also exerted substantial antiproliferative effects against U-87 cell line with a concentration range from 0.90 ± 0.16 μM to 9.23 ± 2.13 μM being inferior to the original C7-phenyl analogue but still preserving submicromolar potency, except only one derivative demonstrating micromolar potency (Table 6). The drop of the activity of this analogue could be also explained by its polar character impairing cell membrane permeability.

Synthetic 7-deazahypoxanthines were shown to be capable of disrupting microtubule cytoskeleton organization in the tumor cells via binding to the colchicine site of β-tubulin [79]. One of the 7-deazahypoxanthines at concentrations between 1 and 2 μM induced significant alterations of the mitotic microtubule organization when cultivating on the HeLa cell line. The interphase microtubules were less affected at these concentration range suggesting that this compound was primarily affecting dynamic microtubules. It exerted slight influence on the stable interphase or spindle microtubules but at the same time induced a marked mitotic spindle shift that may be related to the astral microtubule defects. Astral microtubules as the most dynamic microtubule population at mitotic stage are likely to be the most sensitive targets for tubulin-targeting drugs such as 7-deazahypoxanthines [79].

### 2.5. Pyrrospirone Alkaloids

**Figure 5:**
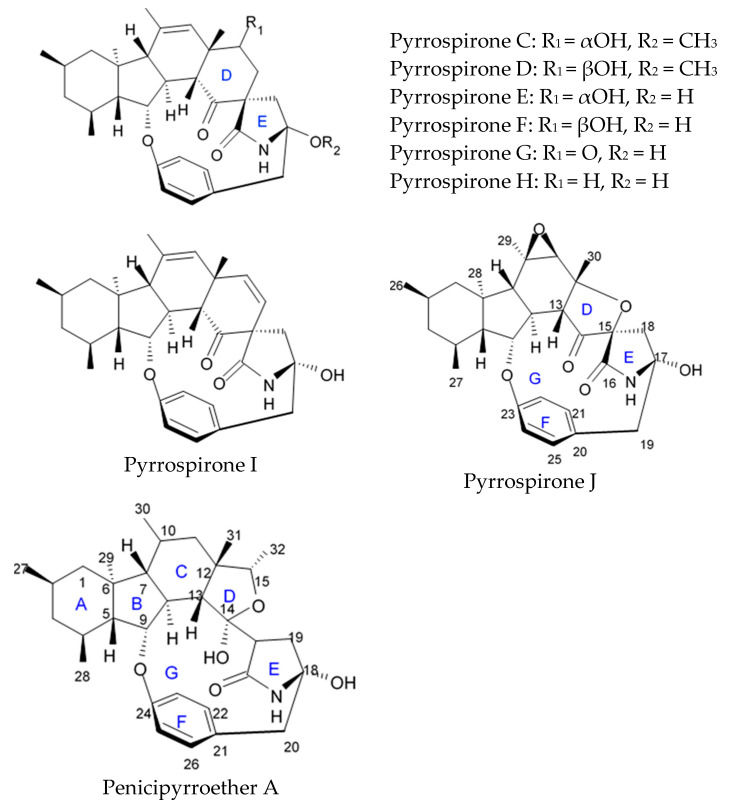
Structure of pyrrospirones C—J and penicipyrroether A isolated from the cultures of marine-derived fungus *Penicillium* sp. ZZ380 [81].

Novel pyrrospirone alkaloids C, D, E, F, G, H, and J as well as penicipyrroether A were isolated from marine fungus *Penicillium* sp. (ZZ380 strains) generally found in the wild sea crab *Pachygrapsus crassipes*. These alkaloids along with other chemically related compounds make a family of fungal secondary metabolites, which are also called hirsutellones and contain a unique 13-membered ether ring composed of specific structural units such as decahydrofluorene, para-cyclophane, and pyrrolidinone. That fungal metabolite family were isolated from fungi belongings to the genera Cylindrocarpon, Embellisia, Hirsutella, Lewia, Neonectria, Penicillium, and Trichoderma and are divided into four chemical groups: hirsutellones, pyrrospirones, pyrrocidines, and GKK1032s [80]. Pyrrospirones C and D have the same molecular formula (Figure 5). From the structural point of view, both of them are composed of two carbonyls, six aromatic carbons, two olefin carbons, four quaternary carbons, two oxymethines, one methoxyl, six methines, five methylenes, and five methyls as well as they have closed spiro ring system but different configurations at C-17. In a similar fashion, pyrrospirones E and F have the same molecular formula. The ^13^C NMR data of pyrrospirones E and F show close similarities with those of pyrrospirones C and D, respectively, except the different chemical shifts for C-19 to C-21 due to the absence of a methoxy at C-19 in pyrrospirones E and F. This indicates pyrrospirones E and F are the analogues of pyrrospirones C and D without methoxyl at C-19. Similar to pyrrospirones C and D, the structural difference between pyrrospirones E and F is related to different C-17 configurations. Molecular formula of pyrrospirones G is two protons less than that of pyrrospirones E. Structural difference between pyrrospirones G and E presented with oxymethine at C-17 in E is replaced by a carbonyl group in G compound. That is 16 mass units less than that of pyrrospirone E. Pyrrospirone H is considered a pyrrospirone E analogue with a methylene attached at C-17. Pyrrospirone I is two protons less than that of pyrrospirone H. The pyrrospirone I structure is considered as analogue of pyrrospirone H with a double bond at C-16 and C-17 [80]. Despite structural parts of rings A–C, F, and G for penicipyrroether A and pyrrospirones C–I are the same, the structures of penicipyrroether A and pyrrospirones have specific differences. First of all, D ring contains five-membered ether ring for penicipyrroether A and cyclohexanone for pyrrospirones. Then, penicipyrroether A does not have spiro junction for rings D and E. NMR spectroscopic data analysis and HRESIMS data (high resolution electrospray ionization mass spectroscopy) demonstrated that compound pyrrospirone J is an analogue of penicipyrroether A with the same structural part of rings A, B, F, and G but possesses an epoxy moiety at C-10 and C-11 and a different five-membered ether ring D fused with ring E through a spiro carbon of C-15. In addition, the dehydro-pyrrolidinone moiety (ring E) in penicipyrroether A is replaced by a pyrrolidinone moiety in pyrrospirone J [81].

Inhibiting activity regarding proliferation of the glioma cells U87MG, U251, SHG44, and C6 of the pyrrospirone alkaloids isolated from the ZZ380 strain were investigated. The results showed that pyrrospirone G exerts relatively strong activity towards aforementioned cell lines with IC_50_ values approximately between 1.06 and 8.52 μM. Pyrrospirones C, D, E, F, H, I, and J have demonstrated moderate anti-glioma activity with IC_50_ values generally between 7.44 and 26.64 μM (Table 7) [80]. Penicipyrroether A exerted strong inhibiting influence on the cell proliferation in glioma lines U87MG and U251 with the IC_50_ values 1.64 and 5.50 μM, relatively [81]. Doxorubicin used as a positive control have suppressed glioma cell proliferation with IC_50_ approximately 1.20 and 8.03 μM, respectively. These results suggest that antiglioma activity of penicipyrroether A is equivalent or slightly stronger than doxorubicin potency. Furthermore, cytotoxicity of penicipyrroether A and doxorubicin against normal human astrocytes (HA, cat. no. 1800, ScienCell, Carlsbad, CA, USA), expressed in CC_50_ was shown to be 23.28 ± 1.05 μM and 8.57 ± 0.16 μM, respectively. Selectivity index (CC_50_/IC_50_) of penicipyrroether A towards U87MG and U251 cells was found to be 14.2 and 4.2, respectively, which is substantially higher than that of doxorubicin i.e., 7.1 and 1.1, respectively [81].

Penicipyrrodiether A containing phenol A derivative fused to the pyrrolidinone core via the five membered ether ring added have shown the moderate antiglioma activity manifesting in reduced proliferation of the glioma U87-MG, U251, SHG-44, and C6 cells with IC_50_ values between 11.32 and 29.10 μM. Doxorubicin used as positive control was found to exert its activity with IC_50_ values 0.70 to 9.61 μM [80].

Therefore, among the pyrrospirone alkaloids that have been studied, at least two compounds namely pyrrospirone G and penicipyrroether A are worth attention as promising antiglioblastoma agents.

### 2.6. Pyrrocidine Alkaloid

**Figure 6:**
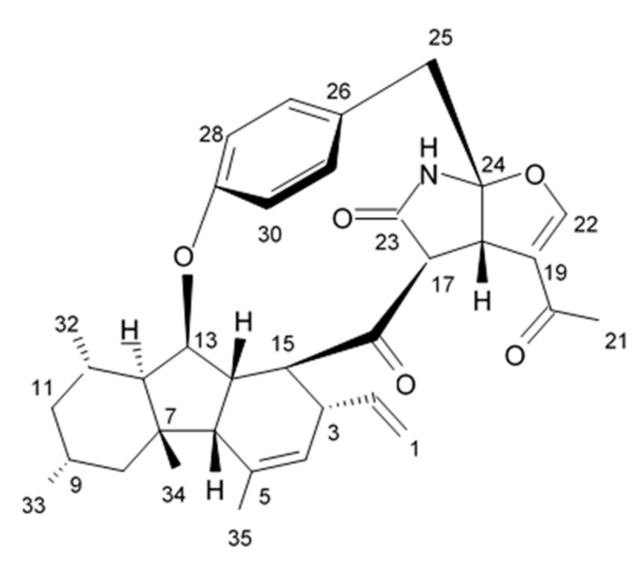
Chemical structure of trichobamide A from ascidian-derived fungus *Trichobotrys effuse* 4729 [82].

Novel pyrrocidine alkaloid trichobamide A isolated from the ascidian-derived fungus *Trichobotrys effuse* 4729 had shown significant inhibition of the cell proliferation in two glioma lines such as U251 and SNB19. This novel alkaloid is different due to the presence of unprecedented tetrahydro-5H-furo[2,3-b]pyrrol-5-one moiety (Figure 6). Structurally, trichobamide A represents the first example of tetrahydro-5H-furo[2,3-b]pyrrol-5-one ring system and biosynthetically is related to a family of pyrrocidine alkaloids bearing a macrocyclic ether and succinimide-derived moieties.

Within the concentration range from 1 to 10 µM trichobamide A exerted concentration- and time-dependent inhibiting effects against glioma cells. It was found out that the mechanism of antiproliferative activity of trichobamide A is directly related to increased relative expression of proteins P53, Bax, caspase 3, and caspase 9 as well as reduced expression of Bcl-2 in glioma cells. Trichobamide A was discussed to induce apoptosis in glioma cells through a pathway upregulated by P53/Bax/Bcl-2 [82].

### 2.7. Alkaloid Pseurotin A

An alkaloid pseurotin A initially isolated from the cultures of some fungus species such as *Pseudeurotium ovalis*, *Aspergillus* spp. and *Hericium erinaceum*, was later isolated from a culture broth of marine bacterium *Bacillus sp*. (the strain FS8D), marine barnacle *Lepas anatifera*. The pseurotin A was shown to be highly active in reducing proliferation of four different glioma cells with IC_50_ values of 0.51–29.3 μM [83] (Table 8). Doxorubicin used as positive control in the same cell lines had exerted antiglioma activity with IC_50_ 0.5–9.6 μM.

Mechanisms of antitumor activity were investigated in the U87-MG cell line. Pseurotin A was shown to reduce the expression levels of PKM2 and especially LDH5, which is a key enzyme responsible for accelerating conversion of pyruvate to lactate, the final product of tumor glycolysis. In addition, pseurotin A upregulates expression of pyruvate dehydrogenase beta (PDHB), adenosine triphosphate synthase beta (ATPB), and cytochrome C (Cyto-C) [83], which are main enzyme components in the processes of tricarboxylic acid (TCA) cycle and oxidative phosphorylation in normal cells.

Based in the results obtained through the study, authors have proposed that pseurotin A exerts anti-glioma effects via inhibition of the accelerated rate of glycolysis int the glioma cells through the downregulation of PKM2 and LDH5 expression. Also, it alters tumor metabolic pathway to the process of oxidative phosphorylation by enhancing TCA cycle and oxidative phosphorylation activities through the upregulation of PDHB, ATPB, and Cyto-C.

### 2.8. Polycyclic Diamine Alkaloids

Polycyclic diamine alkaloids represent the specific class of alkaloid compounds that are mostly composed ammonia, propenal, and long-chain dialdehydes as the universal building blocks. These alkaloids are typically found in the marine sponges belonging to the order *Haplosclerida* with a major part of species represented with four families of that order (*Callyspongiidae*, *Chalinidae*, *Niphatidae*, and *Petrosiidae*). Two recently described alkaloids namely papuamine and haliclonadiamine as well as two newly discovered compounds called neopetrocyclamine A and neopetrocyclamine B were isolated from the Indonesian sponge *Neopetrosia* cf *exigua* and then screened for their efficacy in vitro against glioblastoma cell line SF-295. A symmetrical diamine compound papuamine was originally discovered from a marine sponge belonging to the genus *Haliclona* from Papua-New Guinea [84] and from Palau [85]. In the Palau specimens, papuamine was just a minor component in its mixture with haliclonadiamine, which is an unsymmetrical diastereoisomer of papuamine.

Neither neopetrocyclamine A nor neopetrocyclamine B had shown significant cytotoxicity at 20 μM. Nevertheless, both alkaloids papuamine and haliclonadiamine demonstrated strong inhibiting influence against glioblastoma cells (Table 9). As it is shown in the table, papuamine is more potent than haliclonadiamine against glioblastoma SF-295 cells because its GI_50_ value is almost 8-fold lower [86]. Authors suggested that the stereogenic center at C-6 of the papuamine play important role in the anti-glioma activity.

Papuamine was recently shown to possess antimetastatic activity against MDA-MB-231 breast cancer cells and reduce tumor cell vitality via mitochondria damage and JNK activation [87]. However, specific molecular targets and mechanisms of the anti-glioma effects exerted by these compounds are to be studied yet.

### 2.9. Polycyclic Granulatimide Alkaloids

Alkaloids granulatimide and isogranulatimide isolated from ascidian *Didemnum granulatum* were shown to exert inhibiting activity against checkpoint kinase 1 (Chk1) [88].

The result of the molecular docking study suggested that amino group at the para-indolic position in the granulatimide framework could be directed towards the binding site opening. After that, 17 amido and amino analogues of granulatimide and isogranulatimide were tested in the oligodendroglioma Hs683 and glioblastoma U373 cells and the results demonstrated that two derivatives with open structure exerted antiglioma activity exceeding that of the granulatimide. Their anticancer effects were noted at micromolar concentrations (Table 10) [89]. It is worth noting that the closure of the open structure into the corresponding final product usually resulted in the lowered in vitro tumor growth inhibition. Nevertheless, no relevant inhibition of Chk1 was detectable with these structures [89]. That may suggest the presence of other pathways playing important role in the inhibition of the glioma cell proliferation.

## 3. Antitumor Antibiotics

Main sources of the marine-derived antimicrobial agents are bacteria (including bacteria from marine sediments and marine water samples, marine alga-associated and marine/mangrove plant-associated bacteria, and invertebrate-associated bacteria), fungi (including fungi from marine sediments and marine water samples, marine alga-, plant-, and invertebrate-associated fungi), algae, and invertebrates (mainly, marine sponges and ascidians) [90]. In addition to their antimicrobial activity, some antibiotics provide antiproliferative influence playing valuable role in chemotherapy of malignant tumors. Therefore, they are commonly termed antitumor antibiotics [91]. In the present review section, we will turn our attention to antibiotics with antiglioma activity.

### 3.1. Actinomycins

Actinomycins present a family of closely related chromopeptides exerting both anticancer and antibiotic activities and obtained from a variety of streptomyces strains. Each strain synthesizes a unique set of actinomycin compounds. All actinomycins consist of 2-amino-4,6-dimethyl-3-oxo-phenoxazine-1,9-dicarboxylic chromophore with two pentapeptide lactone rings separately connected to one of the carboxylic groups in positions 1 and 9 in the phenoxazinone core. Variations in the actinomycin structure are possible but restricted to amino acid substitutions in the peptide lactone rings. Phenoxazinone core is unModified as it is essential for their activity.

To date, about 50 actinomycins have been isolated from various species of *Streptomyces* including actinomycin D, N-demethylactinomycins, actinomycins C, F, Z, G, and Y. Some actinomycin analogues, such as methylated actinomycin D and actinomycin Z analogue having additional oxygen bridge between chromophore and β-depsipentapeptide as well as actinomycins D1–D4 and neo-actinomycins A and B, contain structurally modified cyclopeptide rings or chromophore [92]. Inside tumor cell the core chromophore phenoxazinone intercalates between the stacked nucleobases at the DNA guanine/cytosine sites whereas the pentapeptide elements bind to the minor groove. These binding interactions effectively inhibit replication and transcription processes in the tumor cells [93].

Actinomycin D, actinomycin V, and actinomycin X_0β_ were isolated from the *Streptomyces* sp. ZZ338 of marine origin and then tested for their activity inhibiting proliferation of U251, SHG44, and C6 cells (Table 11). All tested compounds were shown to have high potency regarding cell proliferation inhibition in three glioma cell lines when applied in a nanomolar half maximal inhibitory concentration (IC_50_). Doxorubicin served as positive control have shown anti-glioma effects within IC_50_ range from 0.70 to 9.61 µM. In experimental settings, actinomycin D significantly downregulated expression levels of HK2, PKM2, glutaminase (GLS), and fatty acid synthase (FASN) [94]. These results suggest that glioma metabolic enzymes of glycolysis, glutaminolysis, and lipogenesis are the potential targets in the anticancer pathway initiated by actinomycins.

### 3.2. Antimycins

Up to date more than forty natural antimycins A were discovered including deacyl antimycins exhibiting antifungal, anti-inflammatory, insecticidal, and anticancer activities [95]. Natural antimycins A contain unique nine-membered dilactone core conjugated with a 3-formyl aminosalicylic acid moiety.

Two recently discovered benzamide noncyclic dilactones neoantimycin A and neoantimycin B as well as already known antimycins A1ab, A2a, and A9 were isolated from the culture broth of the actinomycete *Streptomyces antibiotics* H12-15 obtained from the sea sediment collected in mangrove area. Aforementioned compounds were investigated regarding their cytotoxic activity against human glioblastoma cells SF-268. Both neoantimycins have demonstrated moderate cytotoxic activity, which was comparable to the one of anticancer drug cis-dichlorodiamine platinum. Antimycins A1ab, A2a, and A9 contain 3-aminosalicylic acid and proved to be scientifically more potent by 20 and 26 times with IC_50_ values below 1.6 μg/mL (Table 12). According to the results of the cytotoxicity assay study [96], it may be concluded that dilactone ring may be directly involved in the interaction between antimycin A and its site of action structures.

### 3.3. Anthracyclines

Biologically active compound SZ-685C, an anthracycline analogue isolated from the secondary metabolite of mangrove endophytic fungus #1403 collected in the South China Sea was tested for its antitumor activity in the glioma cell line LN-444. SZ-685C was shown to inhibit cell vitality of the glioma cells with IC_50_ around 7.8 μM after 48 h since the drug administration (Table 13) [97].

It was shown in the human breast cancer cell lines MCF-7 and MDA-MB-435 that SZ-685C compound selectively kills cancer cells via activation of both caspase-8- and caspase-9-based apoptotic pathways by suppressed whole or at least part of the Akt phosphorylation. SZ-685C inactivates Akt in the breath cancer cells that in turn results in reduced FOXO1 and FOXO3a phosphorylation and consequently leads to increased expression of its downstream target genes, such as Bim. Upregulation of Bim, which can bind to Bcl-2 and Bcl-XL via its BH3 domain, thereby sequestering Bcl-2 from the pro-apoptotic Bax and Bad, and initiating the apoptotic cascade, has been seen in breast cancer cells treated by SZ-685C [97]. Despite SZ-685C is already considered as promising anti-Akt and antitumor drug candidate, the mechanisms of its cytotoxic effects regarding glioma cells are not cleared out yet.

### 3.4. Bagremycins

A crude extract prepared from a culture of a mangrove-derived actinomycete, *Streptomyces* sp. Q22, isolated from a sample of mangrove soil was found to be active against glioma cells. A large culture of this actinomycete in liquid medium was used for isolation of eight compounds, bagremycins A, B, C, D, and E, bagrelactone A, and two styrene derivatives (4-hydroxystyrene and 4-hydroxystyrene 4-O-α-D-galactopyranoside). Bagremycins C−E and bagrelactone A were found to be the new compounds. Activity of all isolated compounds against U87MG, U251, SHG44, and C6 was assayed. The results indicated bagremycin C is active against four different glioma cell lines with IC_50_ values within a range between 2.2 and 6.4 μM (72 h), compared to the activity of the doxorubicin (0.4 to 3.3 μM) (Table 14). Bagremycin B also showed similar activity with IC_50_ values 7.3 to 13.3 μM [98].

Bagremycin C was found to induce apoptosis in U87MG cells in a dose- and time-dependent manner and provoke the U87MG cell cycle arrest at G_0_/G_1_ phase. The total number of apoptotic cells induced by bagremycin C was increased by 20.59% (48 h) and 58.32% (72 h) at 2.2 μM and 61.16% (48 h) and 93.00% (72 h) at 4.4 μM, respectively, compared to the control (5.80% for 48 h and 5.24% for 72 h). Doxorubicin (10.0 μM) induced apoptosis in the U87MG cells by 10.42% (48 h) with 26.87% (72 h) increase of the total apoptotic cell number. Moreover, cell cycle assay showed that the cell population at the G_0_/G_1_ phase was enhanced by 16.18% and 20.56% after 12 h of exposure, respectively, to 4.4 and 8.8 μM bagremycin C concentration [98]. These changes occurring in the cell cycle indicated that bagremycin C might block the glioma cell cycle at the G_0_/G_1_ phase.

### 3.5. Capoamycins

**Figure 7:**
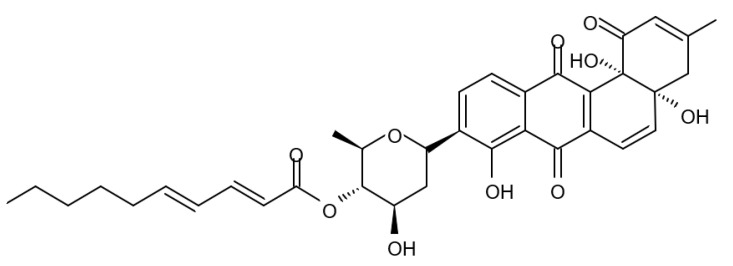
Structure of capoamycin.

Antibiotic agent capoamycin was originally isolated from the culture broth of soil *Streptomyces capoamus* strain no. 23–41. Its characteristic structure consists of a modified benz[a]anthraquinone chromophore, a deoxysugar unit and a long chain polyene acid (Figure 7). Capoamycin has been reported to inhibit the growth of gram-positive bacteria, yeasts and fungi as well as it induces differentiation of mouse myeloid leukemia cells, and prolongs survival periods of mice with xenografted Ehrlich ascites carcinoma [99]. Capoamycin type antibiotic compound MK844-mF10 was initially isolated from the soil strain of *Streptomyces* sp. MK844-mF10 and later identified in the cultural media of the *Streptomyces fradiae* PTZ00025 strain found in the marine precipitates along with two novel compounds named fradimycin A and fradimycin B.

All three marine antibiotics have shown strong dose-dependent activity toward inhibition of the glioma C6 cell growth in the concentration range between 0.125 and 40 μM with the average IC_50_ values from 0.47 to 1.31 μM (Table 15). Fradimycin B was found to be the most active compound in concentration between 0.625 and 1.25 μM and it induced apoptosis and necrosis in C6 cells as well as it blocked cell cycle at the phase G_0_/G_1_ in the human colon HCT-15 cell line [100]. Polyenoic acids assigned as fradic acid A and fradic acid B were isolated with fradimycins from *Streptomyces fradiae* PTZ00025 and later found to be inactive.

### 3.6. Polyene-Polyol Macrolides

Polyene-polyol macrolides are mainly microbial metabolites with the main structural elements presented as a continuously conjugated chain of four to seven unsubstituted double bonds connected to a polyol fragment with alternating hydroxy groups.

Some of these polyene-polyol macrolides were found to have significant antifungal, antibacterial, antiviral, and antitumor activities [101]. During the search for the new antioglioma antibiotic in the marine sources, among 10 bacterial strains isolated from a sample of mangrove soils, extract made of Streptomyces sp. ZQ4BG was found to be the most active one against the proliferation of glioma cells U87MG and U251. Several polyene-polyol macrolides were isolated from above-mentioned strain and identified. They include well-known flavofungins I and II, that wre previously isolated from the actynomicet I, a couple of new compounds named flavofungins III–IX as well as previously known spectinabilin, a nitro-containing metabolite first isolated from *Streptomyces spectabilis*. Chemical structures of the new compounds isolated from ZQ4BG are given in the Table 16.

Flavofungins I and II were 32-membered polyene-polyol macrolides with a conjugated chain of five unsubstituted double bonds connected to a polyol fragment with eight hydroxy groups alternated. New compounds named as flavofungins III–IX are new members of flavofungins with only four unsubstituted conjugated double bonds and more oxygenated moieties. Flavofungin III is an analogue of flavofungin I with two hydroxy groups substituted at C-10 and C-11. Flavofungins IV–IX are rare 32-membered polyene-polyol macrolides with a tetrahydrofuran ring between C-10 and C-13 for flavofungins IV–VIII or a unique oxepane group (ring) between C-10 and C-15 for flavofungin IX.

All 10 isolated compounds were evaluated for their activity in inhibiting proliferation of glioma cells U251, U87MG, SHG44, and C6, and doxorubicin was used as a positive control. Flavofungin II and spectinabilin showed moderate activity against the proliferation of four tested glioma cell lines. Flavofungin I showed weak activity (Table 16). Flavofungins III–IX were inactive. The cytotoxicity of the flavofungin II and spectinabilin towards normal human astrocyte and human foreskin fibroblast 1 cells were also assayed. Both compounds showed a higher selectivity index (CC_50_/IC_50_) for normal astrocytes with 4.6–16.5 for flavofungin II and 3.4–13.3 for spectinabilin, when compared to the selectivity index for fibroblasts with 1.8–6.4 for flavofungin II and 1.3–5.1 for spectinabilin [102].

## 4. Saponins

Saponins are the natural glycosides with triterpene or steroid aglycon. Steroid saponins are generally found in starfishes, while triterpenoid saponins are mainly evidenced in sea cucumbers. Steroid saponins are divided into three groups: asterosaponins, polyhydroxysteroidal glycosides and basically rare type of these compounds, cyclic glycosides. Asterosaponins have typical structural patterns including an aglycone with Δ9(11),3β,6β-dihydroxysterodal nucleus and a sulfate group at C-3. The oligosaccharide chain generally is comprised of five or six sugar units glycosidically linked at C-6 and is made up of the structural diversity of asterosaponins. The sugar moieties of saponins are mostly presented with D- or L-forms of arabinose (Ara), fucose (Fuc), quinovose (Qui), xylose (Xyl), galactose (Gal), and glucose (Glc) [103].

### 4.1. Steroid Saponins

The first research study focused on antiglioma activity of the marine saponins [104], showed the a compound named asterosaponin 1 and isolated from the starfish *Culcita novaeguineae* and formulated as sodium (20S)-6α-O-{β-D-fucopyranosyl-(1→2)-α-L-arabinopyranosyl-(1→4)-[β-D-quinovopyranosyl-(1→2)]-β-D-xylopyranosyl-(1→3)-β-D-quinovopyranosyl}-20-hydorxy-23-oxo-5α-cholest-9(11)-en-3β-yl-sulfate) when dosed in a range from 2.5 to 20 μg/mL significantly suppressed U87MG cell proliferation in a time- and dose-dependent manner (Table 17).

Treatment with 3.4 and 4.3 μg/mL of asterosaponin 1 induced S phase arrest, whereas application of 10.0 μg/mL of asterosaponin 1 resulted in the G_0_/G_1_ phase arrest. Percentage of cells being in the G_2_/M phase was decreased, while the number of cells in G_0_/G_1_ cycle phase increased in 24 h since the treatment start. In this study, glioma cells demonstrated typical morphological apoptosis signs such as nuclear condensation with apoptotic bodies and DNA fragmentation. Furthermore, asterosaponin 1 decreased the expression of Bcl-2 protein and increased the expression of the Bax protein [104].

It was shown later that at least four more asterosaponins isolated from the starfish *C. novaeguinea* exerted significant inhibiting activity against various human and mouse glioblastoma cell lines in vitro, but did not affect the primary cultured human astrocytes (Table 18) suggesting good safety profile of asterosaponins in vitro. These results suggest that 20α-hydroxy is an effective functional group determining anti-glioblastoma activity of asterosaponins. Three asterosaponins exhibited tubulin polymerization activity, and one of them initially affected microtubule function and then blocked U87MG cell cycle resulting in mitochondrial damage, cristae disorganization, membrane potential collapse, cytochrome C release, and finally activated caspase 3 and induced apoptosis [103].

Apart from asterosaponins, there are four new polyhydroxysteroidal glycosides called culcinosides A, B, C, and D presenting along with three known glycosides identified as echinasteroside C, linckoside F, and linckoside L3. These compounds were isolated from the ethanol extract of starfish *C. novaeguineae* and their cytotoxicity was evaluated on the human glioblastoma cell lines U87, U251, and SHG44. Generally, polyhydroxysteroidal glycosides consist of an oxygenated steroidal aglycone with more than three hydroxy groups, and one or two (rarely three) monosaccharide residues attached to steroidal nucleus or a side chain. C Culcinoside A had shown the most potent and strongest cytotoxic activity. Culcinosides B, C, and D demonstrated moderate activity (Table 19). Average IC_50_ for doxorubicin tested on the same cell models were approximately 28.3, 47, and 53.6 times lower than those of culcinoside A [105]. Eleven novel polyhydroxysteroid glycosides ranging from anthenoside A to anthenoside K were isolated from the starfish *Anthenea chinensis*. Seven of them were found to exert selective inhibiting activity against K-562 and BEL-7402 cells. Three of these glycosides were active in the U87MG cell lines. Anthenoside A was shown to have substantially high cytotoxicity against U87MG cells (Table 20) [103,106,107]. In addition, it exhibited tubulin polymerization promoting activity [106]. The structure–activity relationship analysis indicated that the monosaccharide units and their attachment sites on aglycone might be considered important for promoting tubulin polymerization and inhibiting glioma cell proliferation [107].

**Figure 8:**
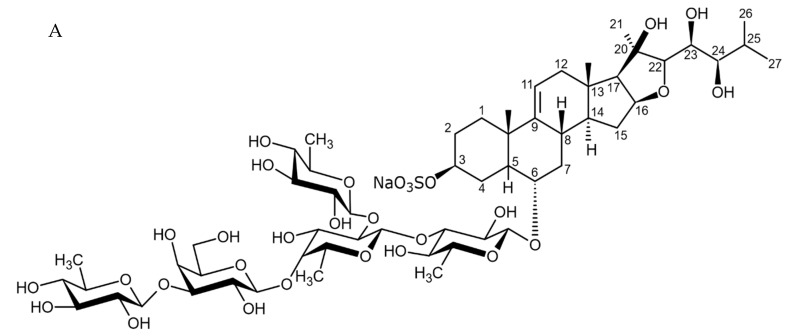

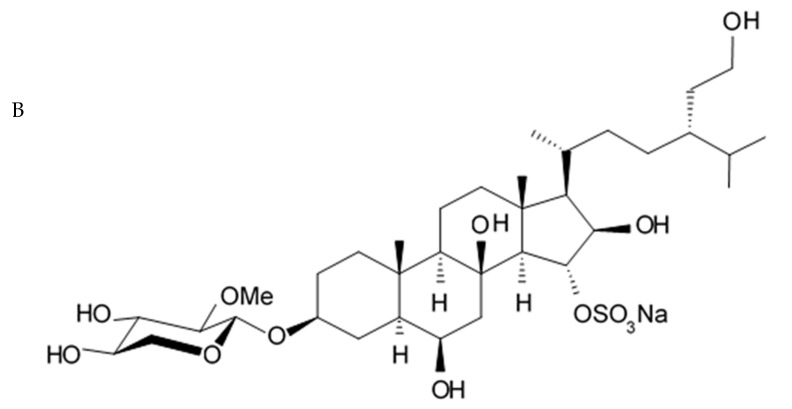
Asterosaponin (**A**) and polyhydroxysteroidal glycoside (**B**) from *Pentaceraster chinensis* exert antitumor activity against U87MG glioma cell line.

New asterosaponin and polyhydroxysteroid saponin found in starfish *Pentaceraster chinensis* (Figure 8) exerted strong cytotoxicity in the U87MG cell lines resulting in apoptosis modulated upregulation of Bax protein and downregulation of Bcl-2 protein [108].

### 4.2. Triterpenoid Saponins

Holothurian triterpene glycosides composed of oligosaccharide chain and holostane3-ol based aglycone often contain double bond between C-9 and C-11 or between C-7 and C-8 in the aglycone. Carbohydrate chain encloses up to six sugars units including xylose, glucose, 3-O-methylglucose, and quinovose that can be branched only once. The inter-specific differences in the triterpene glycoside structure are about the presence or absence of sulfate groups attached to the carbohydrate chain. More than 350 triterpenoid saponins from the sea cucumbers were described so far, and a plenty of them possess cytotoxicity against various cancer cell lines [32].

The new holostane-type triterpene glycosides, namely pentactasides I, II, and III, and two previously known glycosides philinopsides A and B were isolated from the sea cucumber *Pentacta quadrangularis*. Despite structural differences all isolated glycosides inhibited U87MG glioma cell line with IC_50_ ranging between 1.90 and 3.95 μM (Table 21) [109].

**Figure 9:**
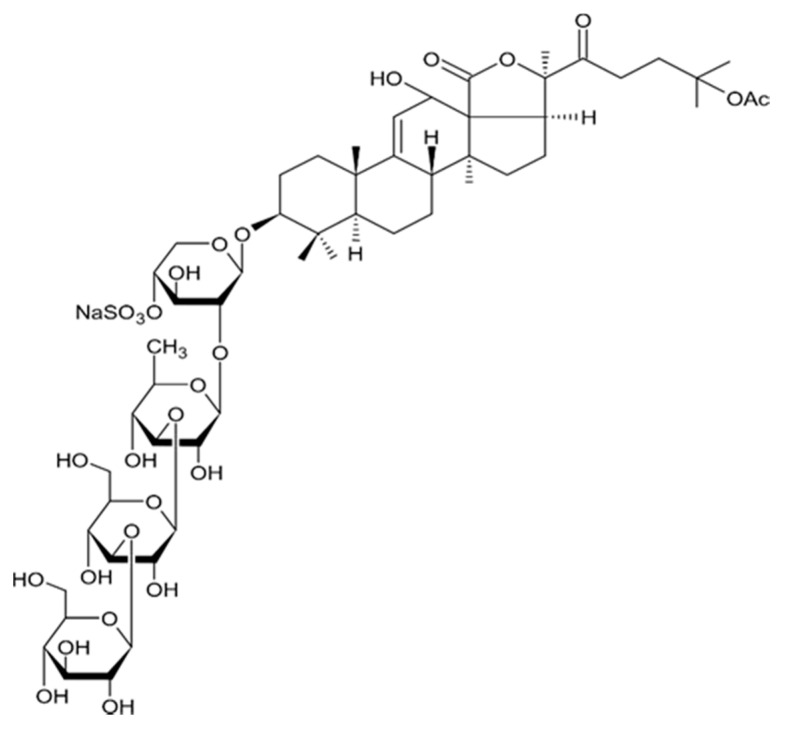
Fuscocineroside A from the sea cucumber *Holothuria fuscocinerea* exerting antiproliferative activity regarding glioma U251 cells [110].

The new triterpene glycoside fuscocineroside A shown on Figure 9 was isolated from the sea cucumber *Holothuria fuscocinerea*. It inhibited U251 cell proliferation in a dose- and time-dependent manner, induced apoptosis, and reduced surivin expression in the glioma cells, but at the same time, showed slight cytotoxicity regarding normal human astrocytes [110].

**Figure 10:**
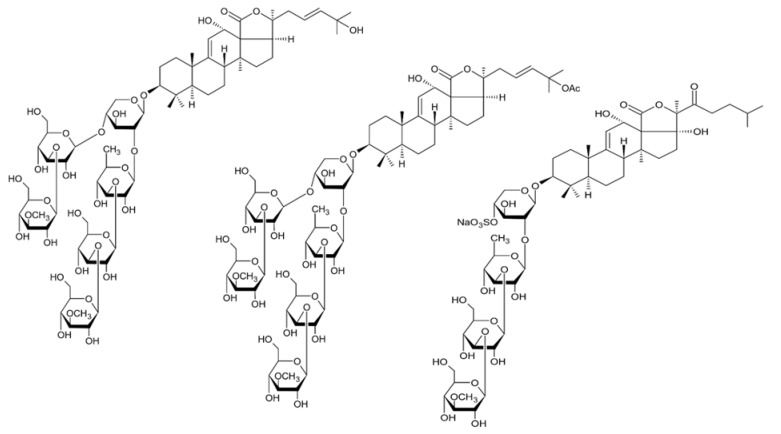
Triterpene glycosides from the sea cucumber *Bohadschia marmorata* exerting antiproliferating activity against glioma U87MG cells [103].

Three new holostan-type triterpene glycosides given on Figure 10 were isolated from the sea cucumber *Bohadschia marmorata* and induced significant inhibition of cell proliferation in five tumor lines including U87MG cells [103].

**Figure 11:**
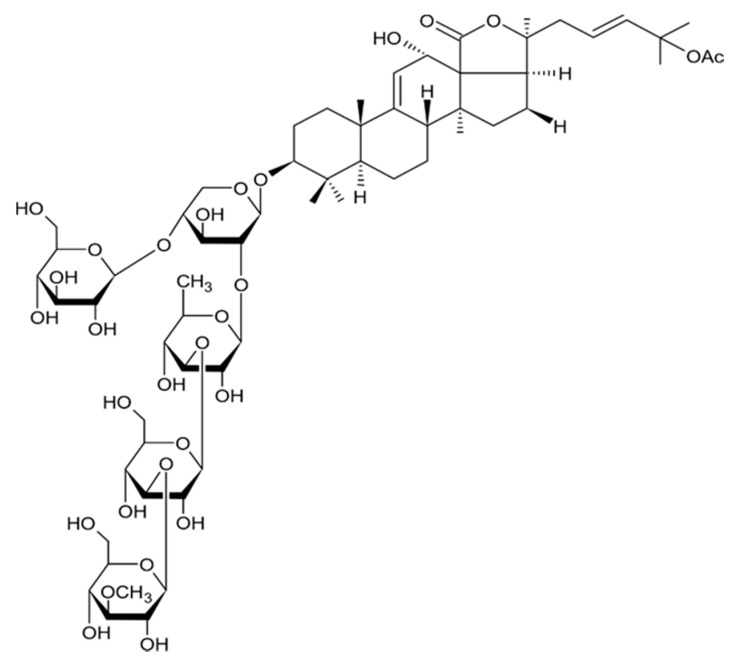
Pervicoside D from the sea cucumber *Holoturia axiloga* [103].

Another holostan-type triterpene glycoside, pervicoside D (Figure 11) was isolated from the sea cucumber *Holoturia* (*Microthele*) *axiloga* [111] and was confirmed to exhibit significant inhibiting activity against U87MG cells [103].

Ethyl acetate extract fraction of the sea cucumber *Holothuria scabra* body wall was applied in a dose range from 1 to 100 mkg/mL resulting in the substantial reduction of the cell vitality of the glioblastoma lines A172 and U87MG with IC_50_ 4.23 and 4.46 μg/mL, respectively, in 24 h. Furthermore, that extract was shown to exert greater cytotoxicity in comparison to the antitumor drug temozolomide on both cell lines at the same concentrations. This extract did not exhibit toxicity regarding primary human fetal astrocytes in the concentration range between 0, 1, and 5 μg/mL. In addition, extract induced early and late apoptosis stages, loss of the mitochondrial membrane potential, nuclear condensation and fragmentation.

Treatment of the glioblastoma cells with aforementioned extract for 24 h results in increased expression of proapoptotic Bax and caspase-3 whereas Bcl-2 expression was suppressed in a concentration-dependent manner. The following major morphological changes typical of apoptosis were observed: shortening of the cell processes, detachment and loss of confluence resulting in the cell round-ups, cytoplasmic condensation, and formation of apoptotic bodies. Results of the acetyl acetate holothurian extract separation using HPLC with the following LC-MS/MS data analysis have shown that the extract contain a group of compounds, which mass-spectrum was corresponding to the one of triterpene glycosides identified earlier in the other sea cucumber species. Scabrasides A and B and holothurin A3 found in the *H. scabra* were identified in greater amounts whereas in the less quantities were found substances corresponding triterpene glycosides from *H. forskali*, *H. lesson*, *Athyonidium chilensis*, and *Apostichopus japonicus* [112].

Already known sulphated saponins holothurin A, holothurin B, and 24-dehydroechinoside B as well as new triterpenoid saponin (3-O-[β-D-quinovopyranosyl-(1→2)-4-sodium sulfato-β-D-xylopyranosyl]-25-acetoxy-22-oxo-9(11)-holostene-3β,12α,17α-triol), isolated from the sea cucumber *Holothuria moebii* suppressed the proliferation of U87-MG, U251, SHG-44, and C6 cells with IC_50_ values ranging from 0.99 to 8.64 µM (Table 22). An artificial compound desulfated holothurin B had shown moderate or weak activity with IC_50_ values from 14.43 ± 1.33 to 53.01 ± 1.64 µM [113]. That is very likely that the sulfate group at C-4 of xylose is important for the activity of this type of triterpenoid saponins. Temozolomide exerted slight activity regarding C6 and SHG-44 cells and did not display any activity against U87-MG and U251 cells at 100 µM probably due to the cell resistance to temozolomide.

Recently discovered saponin was applied in concentrations 2 and 4 µM inducing apoptosis and necrosis in U87-MG cells in 24 h. Increase of the apoptotic cell number was 60.25% (2 µM) and 55.02% (4 µM), increase of necrotic cell amount was 0.23% (2 µM) and 10.47% (4 µM), respectively, whereas total number of both apoptotic and necrotic cells was 60.48% (2 µM) and 65.49% (4 µM) higher in comparison to control. Investigation of the effects exerted by the new sulfated saponin on proapoptotic genes BCL-2/BCL-XI-associated death promoter (BAD) and BCL-2-associated X protein (BAX) and on anti-apoptotic genes B-cell lymphoma 2 (BCL-2) and B-cell lymphoma-extra-large (BCL-XL) showed this saponin does not regulate expression level of both pro- and anti-apoptotic genes. The results of the study devoted to the influence of this sulfated saponin on the expression levels of glioma metabolic enzymes of glycolysis and glutaminolysis demonstrated that this compound (4 µM) significantly lowers the expression levels of HK2, PFKFB3, PKM2, and GLS in U87-MG cells after 24 h treatment [113]. At the same time, it did not affect expression of the enzyme in the normal human astrocytes. It did not influence the expression levels of aconitase 2, ATPB, pyruvate dehydrogenase beta, and CytoC as well [113], which are important regulators in the processes of the tricarboxylic acid cycle and oxidative phosphorylation involved into the glucose metabolism in normal cells. This makes a suggestion that holothurian sulfated saponins have unique antitumor mechanism selectively targeting glioma metabolic regulators of glycolysis and glutaminolysis.

Having discussion about substantial antiglioma activity of saponins in vitro, it should be pointed out that systemic administration of pharmaceuticals containing saponins is barely possible due to such side effect as hemolysis [114]. However due to the development of interstitial chemotherapeutic methods against glioblastoma in clinic and positive results of saponin administration in experimental animals by in situ administration with interstitial injection or catheter insertion through tumor region, the chemotherapeutical potential of the marine saponins may be implemented in a form of drug products purposed for topical administration.

## 5. Terpenes

Terpenes present a class of unsaturated hydrocarbons with general formula (C_5_H_8_)n with *n* > 2 [115] which are categorized into monoterpenes (C10H16), sesquiterpenes (C_15_H_24_), diterpenes (C_20_H_32_), triterpenoids (C_30_H_48_), and tetraterpenes. Terpenoid sources include as terrestrial higher plants as marine species (bacteria, fungi, micro-, and macroalgae, sponges, and others). Chemical synthesis as well as semisynthetic assembly have been playing a significant role in the terpenoid drug production [116]. Representatives of at least sesquiterpenes, di- and triterpenes was found to exert antitumor activity regarding glioma cells.

### 5.1. Sesquiterpenes

**Figure 12:**
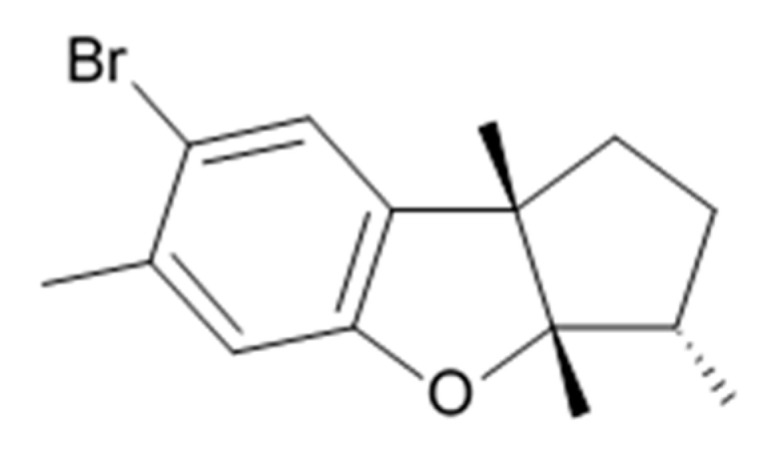
The structure of aplysin.

One of the first halogenated marine sesquiterpenes reported in the literature, aplysin was initially isolated from the opisthobranch gastropod mollusk *Aplysia kurodai* and later—from the red algae belonging to genus *Laurencia*. Aplysin is a bromosesquiterpene with molecular weight 295 (Figure 12). It has attracted much attention because of its potent pharmacological activities including antitumor and antioxidant effects. This compound had shown antitumor influence in the sarcoma, human breast cancer, and human gastric cancer lines via induction of apoptosis [117,118]. Orally fed aplysin purified from the red alga *Laurencia tristicha* was also demonstrated to suppress significantly the growth of 7,12-dimethylbenz[a]anthracene-induced breast tumor tissues in rat models. Tumor growth inhibition activity of aplysin was suggested to related to the PI3K/Akt/FOXO3a pathway inhibition [118].

Aplysin applied in concentrations 10 and 20 μg/mL significantly reduced cell viability in glioma lines U87 MG, U251 MG, U373 MG, and M059J in a dose-dependent manner [119]. Aplysin concentrations 20, 40, and 80 μg/mL significantly inhibited glioma GL26 cell proliferation rate in 24, 48, and 72 h and this effect was found to be time- and dose-dependent. After 48 h treatment with aplysin, the number of GL26 cells in G_0_/G_1_ phase was significantly higher compared with the control, whereas the numbers of cells in S and G_2_/M phases were much lower [120]. In contrast, no significant cytotoxicity was observed in normal astrocytes as a result of the aplysin treatment within the concentration range 1–20 μg/mL. Apoptosis detection revealed that aplysin concentrations 5 and 10 μg/mL can trigger apoptotic events in U-87 MG cell line in a dose-dependent fashion, with apoptosis noted in healthy astrocytes. These results confirm that aplysin selectively triggers apoptosis and cytotoxicity pathway in glioma cells. The colony-forming and transwell assays detecting the colony formation and invasion ability of cancer cells affected by drug treatment showed that the number of colonies was substantially reduced in the glioma cells treated with aplysin if compared to control. Transwell assay results demonstrated that aplysin can suppress glioma cell invasion capacity in a dose-dependent manner. Investigation of the expression profile of eighteen miRNAs related to the glioma progression revealed that miR-181, a potent tumor suppressor, was overexpressed in U-87 MG cell lines treated with 20 μg/mL aplysin [119]. miR-181 is known to suppress the malignant properties of glioma cells by inhibiting tumor growth, inducing apoptosis, and suppressing invasive capacities. Identified miR-181 targets include Bcl-2 and MEK1 [121,122], both of which are known oncogenes that facilitate glioma progression. Aplysin significantly decreased the expression both of MEK1 and Bcl-2 in a dose-dependent manner in the U-87 MG cells.

Exploration Akt and p-Akt protein expression in the glioma GL26 cells treated with aplysin showed that phosphorylated Akt expression was particularly reduced in glioma, while the total Akt level remained unchanged suggesting that aplysin may selectively inhibit PI3K/Akt signaling pathway. The level of Akt-binding heat shock protein 90 (Hsp90) was found to be decreased in the dose-dependent manner, while the total Hsp90 concentration remained unchanged suggesting that aplysin inhibits the Hsp90/Akt complex formation. These effects and pathway are possibly responsible for the growth inhibition of the glioma GL26 xenografts induced by aplysin with following prolonged survival period in mice with grafted tumor approximately by 1.5 and 2 times after drug administration of 40 and 80 µg/kg body weight, respectively [119].

Combined administration of aplysin and temozolomide was demonstrated to significantly increase glioma cell sensitivity to the effects of temozolomide if compared to single administration of temozolomide [119]. Taking to consideration the earlier received information about miR-181 enhancing glioma cell sensitization to temozolomide by silencing MEK1 expression [122], it may be suggested that aplysin-induced temozolomide sensitivity may be also dependent on MEK1 pathway in glioma cells.

Bcl-2 was also found to be downregulated in glioma cells treated with aplysin, implying that this antiapoptotic protein may be also involved in its effect on the action of temozolomide on glioma cells. Therefore, aplysin can suppress malignant properties of glioma and enhance anti-tumor efficacy of temozolomide making him a prospective lead compound for development of the potent anti-glioma drugs.

### 5.2. Diterpenes

Fourteen compounds namely eupalmerin acetate, isoeupalmerin acetate, 3′-O-acetyl-pseudopterosin U, 3-epi-14-deoxycrassin, asperdicin, pseudoplexauric acid methyl ester, 2-deoxyasperdiol acetate, N-methylpyridinum-3-sulfonate, dinosterol, kallolide A, kallolide A acetate, asperdoil, asperdoil acetate, isoasperdoil isolated from the combined terpenoid-rich fraction of the chloroform-methanol extract of specimens Caribbean gorgonian octocoral *Eunicea succinea*, and one compound, placotrid O isolated from the marine sponge *Plakortis halichondroides* were tested on the cell lines U87-MG and U373-MG for the presence of antitumor activity [123]. Placotrid O was the most potent one among the compounds tested in the study, but its cytotoxicity was substantially lowered after it was stored in solution for a few weeks. IC_50_ value changed from 4.0 to 31.0 µmol/L in the U87-MG cells and from 4.0 to 15.4 µmol/L in the U373-MG cells (Table 23).

Five compounds exerted inhibiting effects on the cell viability in both glioma cell lines with IC_50_ ranging from 5.1 to 64.2 μmol/L (Table 24). The most potent compound among them was eupalmerin acetate belonging to the cembranolide diterpenes. It was stable for several months with IC_50_ values 5.1 μmol/L for U87-MG cells and 6.9 μmol/L for U373-MG cells. This range of IC_50_ is comparable with that of cisplatin, which is in the ranging from 5 to 10 μmol/L for these cell types. Two compounds (pseudoplexauric acid methyl ester and 2-deoxyasperdiol acetate) exerted inhibiting effects against only one cell line in concentration below 100 μmol/L. The other compounds had IC_50_ values greater than 100 μmol/L in both cell lines and were therefore considered not potent enough to serve as anticancer drugs for these cell types.

Further investigations were focused on anti-glioma mechanisms involved into eupalmerin acetate activity. It was found out that eupalmerin acetate induces G2-M arrest and apoptosis in cells of both U87-MG and U373-MG lines, at least, partly activating the mitochondrial pathway and inducing Bax translocation to the mitochondria, as well as via activation of the c-Jun NH2-terminal kinase (JNK) pathway. Eupalmerin acetate in the experiments with nude mice with xenografted tumor nodes obtained from U87-MG cells have demonstrated significant antitumor effects. Authors concluded that this result indicate eupalmerin acetate provides therapeutic efficacy against glioma cells and it as well as similar marine-based compound may hold promise as a clinical anticancer agent [123].

### 5.3. Triterpenes

An isomalabaricane-type triterpene, stellettin B, isolated from the marine sponge *Jaspis stellifera* was experimentally tested on 39 human cancer cell lines including lung, gastric, ovarian, breast, kidney, prostate, and colon cancer lines, melanoma as well as gliomas (U251, SF-295, SF-539, SF-268, SNB-75, and SNB-78). Human glioblastoma cell SF295 exhibited high sensitivity to stellettin B (Table 25). At the same time, stellettin B has shown very weak inhibiting activity towards normal cell lines—such as human mammary epithelial cells (HMEC), human renal tubule epithelial cells (RPTEC), normal human bronchial epithelial cells (NHBE), normal human prostate epithelial cells (PrEC)—with GI_50_ higher than 10 μM, suggesting its relatively selective cytotoxicity against glioma cells compared to normal human cell lines. Stellettin B treatment of the cell lines induced apoptosis in a dose-dependent manner as well as enhanced caspase 3/7 activity, cleavage of poly-(ADP-ribose) polymerase (PARP) and reactive oxygen species (ROS) production in SF295 cells. Stellettin B strongly inhibited Akt phosphorylation but did not affect p-ERK and p-p38 activity and did not inhibited PI3K activity in concentration below 1 μM suggesting that antiproliferative and apoptosis-inducing activity is not attributed to PI3K inhibition [124]. The direct target of stellettin B might be signal protein upstream of Akt other than PI3K. Authors have suggested that stellettin B might be a lead compound for discovery of perspective drug candidate for the treatment of some types of glioblastomas.

## 6. Peptides and Cyclopeptides

Cyclic peptides make a group of compounds built with proteinogenic α-amino acids but, in contrast to the regular proteins, they contain D- configuration α-amino acids and modified L-α-amino acids looped into macrocycles and included into peptide chains. Cyclic depsipeptides are cyclopeptides with one or several amino acids replaced with oxy acids, and therefore, their molecules apart from amide groups contain one or few ester groups [125].

### 6.1. Peptides

In 1988 Nakamura et al. isolated a peptide named tachyplesin I from the hemocytes of the horseshoe crab *Tachypleus tridentatus*, determined its chemical structure, and demonstrated antitumor properties. Later this peptide was synthesized by Hanyu Bioengineering Company (China). Tachyplesin I presents cationic peptide with 17 residues (NH2-K-W-C-F-R-V-C-Y-R-G-I-C-Y-I-R-R-C-R-CONH2) [126]. In addition to antimicrobial activity, tachyplesin I can inhibit proliferation of such tumor cells as human hepatocellular carcinoma SMMC-7721 and TSU prostate cancer cells [127]. Tachyplesin I had also been demonstrated to activate classic complement pathways regulating tumor cell lysis and altering tumor suppressor genes expression and oncogenes to induce cell differentiation and reverse the malignant phenotype [128]. Negatively charged components of cancer cells, which are quite different from neutral normal cells, are more vulnerable by the positively charged cationic peptides, including tachyplesin I. Electrostatic attraction between cancer cell and cationic peptides is thought to play a major role in the selective disruption of cancer cell membranes helping avoid development if the typical drug resistance mechanism [129].

Tachyplesin I applied in concentrations 10, 40, and 80 µg/mL was shown in the in vitro experiments to inhibit the viability and proliferation of gliomaspheres isolated from U251 glioma cell lines in dose-dependent manner due to the plasma membrane damage and induced differentiation of the glioma stem cells [130]. Proteomic analysis using two-dimension difference gel electrophoresis and stable isotope dimethyl labeling based liquid chromatography–mass spectrometry/mass spectrometry revealed that in response to tachyplesin I influence 192 proteins were differentially expressed in U251 gliomaspheres [131]. Major part of those proteins is involved into the cellular metabolism processes, in particular, glycolysis processes, and many proteins are identified as cytoskeleton proteins and lysosomal acid hydrolases. The glycolytic/gluconeogenesis enzymes including alpha-enolase (ENO1), gamma-enolase (ENO2), triosephosphate isomerase (TPI1), and phosphoglycerate kinase 1 (PGK1) were down-regulated in response to tachyplesin I treatment indicating that tachyplesin I may disrupt normal energy metabolism in gliomaspheres via reduced glycolysis.

U251 gliomaspheres treatment with tachyplesin I altered the expression of 18 cytoskeleton proteins. Two of those proteins, namely vimentin and ezrin are known to be involved into the metastasis regulation and down-regulated due to the treatment with tachyplesin I suggesting that cytoskeleton is affected by tachyplesin I as well. Apart of that, tachyplesin I significantly reduced levels of lysosomal protease enzymes, cathepsins B and D, in gliomaspheres that are known to be involved into autophagy and apoptosis pathways. Inhibition of cathepsins B or D activity attenuates extracellular matrix degradation and reduces migration of glioma cells [132]. Those results suggest that tachyplesin I has a potency as a therapeutic agent for glioma by targeting the lysosomal activity. Protein–protein interaction network analysis of differentially expressed proteins showed that DNA topoisomerase 2-alpha (TOP2A) may serve as a possible critical target protein of tachyplesin I. DNA damage and fragmentation induced by covalent binding of TOP2A to DNA as well as forced expression of TOP2A in cells are known to trigger the apoptotic cell death. Furthermore, TOP2A level is known to have close relationship with the potency of anti-tumor drugs and high level of TOP2A regulating the main pathway of drug susceptibility. Vice versa, decreased level of TOP2A mutation can induce the loss of the anti-tumor drug targeting and multiple drug resistance development. Elevated TOP2A level was determined in the U251 gliomaspheres treated with tachyplesin I suggesting possible synergistic effect of tachyplesin I and anticancer drugs targeting TOP2A. Such combination may enhance antiglioma efficiency of chemotherapy [131].

### 6.2. Cyclodepsipeptides

Marine cyanobacteria have continued to be a prolific source of cytotoxic depsipeptides applicable to cancer research. One of notable representatives from the cyanobacterial cyclic peptide group is a natural cyclopeptide product coibamide A that was initially isolated from marine filamentous cyanobacterium Leptolyngbya sp. and named after its geographical location as it was collected from the Coiba Island National Park in Panama. Coibamide A is a lariat depsipeptide that features a highly methylated 22-membered macrocycle with a pseudo-tetrameric side chain (Table 26). The coibamide A in vitro screening tests performed in the National Cancer Institute with 60 human tumor cell lines using anticancer drug screen assay revealed differential picomolar–nanomolar potency as a tumor growth inhibitor against many cell lines and some histological selectivity for several malignant cell types such as CNS-derived tumors, breast, ovarian, and colon cancer cells [133].

Coibamide A induces concentration- and time-dependent cell death in human U87-MG and SF-295 glioblastoma cells. 72-h long influence of coibamide A on the cells from both lines demonstrated reduced proliferation and substantial morphological alterations. At the same time, coibamide A induced cytotoxicity appeared only for nanomolar depsipeptide concentrations (Table 26). Cyclization of the depsipeptide is likely to be a critical determinant of the cellular response to coibamide A because two full-length linearized analogues namely coibamide dehydrated seco acid and coibamide seco acid did not exert cytotoxicity towards cells of both glioblastoma and neuroblastoma [134].

Morphological and biochemical cell death forms induced by coibamide A were different in accordance to the cell types. Glioblastoma SF-295 cells demonstrated caspase-3 activation whereas cell death in glioblastima U87-MG line was characterized with a vast cytoplasmatic vacuolization without obvious apoptotic peculiarities despite these cells were approximately three times more sensitive to coibamide A than Sf-295 cells. Therefore, coibamide A can induce apoptosis in SF-295 cells via activation of a classic caspase-3-dependent pathway and also can effectively trigger cell death in U87-MG glioma cells via an alternate (non-apoptotic) pathway when caspase activity is inhibited. It was shown with the use of biochemical and morphological autophagia criteria that coibamide A induces accumulation of autophagosomes in apoptosis resistant U-87-MG cells—i.e., induced autophagia in those cells. It should be mentioned that coibamide A induced autophagosome storage in the human glioblastoma cells via mTOR-independent mechanism. No change was observed in the phosphorylation state of ULK1 (Ser-757), p70 S6K1 (Thr-389), S6 ribosomal protein (Ser-235/236), and 4EBP-1 (Thr-37/46) [134].

Both cell types of glioblastomas U87-MG and SF-295 in the in vitro tests have shown significant increase of the G_1_-phase cell number in response to the effects of coibamide A and appropriate reduction of the S-phase cells [135]. Therefore, a coibamide A action is phase specific and coibamide A induces a cell cycle block at the G_1_/S phase transition. In addition, coibamide A reduced migration and invasive capacity of U87-MG and SF-295 cells, inhibited extracellular VEGFA secreted from glioblastoma U87-MG cells, attenuated proliferation and migration of HUVECs and selectively decreased expression of VEGFR2 with low nM potency. All these observations suggest the presence of antiangiogenic activity of coibamide A. Intratumor administration of 300 μg/kg coibamide A in nude mice with xenograft glioblastoma model inhibited growth of subcutaneous xenograft U87-MG tumor. Tumor volume in all animals treated with coibamide A remained at initial unchanged level with no significant growth for more than four weeks of experimental therapy [135].

It is well known that U87-MG cells lack the PTEN signaling pathway and have normally functioning p53 signaling pathway whereas as SF-295 cells are lacking both p53 and PTEN. Mutations of these two genes are frequently found in GBM. Analysis of 601 genes obtained from 91 stratified patients with GBM executed in the frames of the “The Cancer Genome Atlas” project showed that the frequency of the p53 and PTEN mutations is 42% and 33%, respectively [136]. Coibamide A exerted cytotoxic influence on six glioma cell lines and four of them (SF-295, U251, SF-539, SNB-19) were characterized with the lack of both signaling pathways PTEN and p53. Two of these lines (SNB-75, SF-268) have disorders of the p53 pathways but normally functioning PTEN pathway. Therefore, coibamide A may be considered as an effective glioma cellular toxin regardless of the p53 and PTEN status. This specific capacity of coibamide A to induce more than one way of killing cancer cells is thought to be especially helpful pharmacological property for antitumor therapy.

Therefore, coibamide A is one of the most potent inhibitors of the human glioblastoma cells with a high drug potential. Despite receptor coibamide A structure is not determined, it should be considered as a realistic clinical candidate.

The known cyclodepsipeptide valinomycin and two new cyclodepsipeptides named as streptodepsipeptide P11A and streptodepsipeptide P11B were isolated from the culture of marine actinomycete *Streptomyces* sp. P11-23B, which was obtained from the marine mud sample [137]. Valinomycin was previously isolated from several *Streptomyces* species and is well known to enhance K+ permeability of several membrane systems including mitochondria, erythrocytes, and lipid bilayers. Valinomycin is composed of four units of D-valine (DVal), L-valine (L-Val), D-α-hydroxyisovaleric acid (D-Hiv), and L-lactate (L-Lac) with a trimer structure of cyclo-(D-Val-L-Lac-L-Val-D-Hiv)3. Streptodepsipeptide was determined as cyclo-(D-Val-L-Lac-L-Val-D-Hiv-D-Val-L-Lac-L-Val-D-Hiv-D-Val-L- Lac-L-Val-D-Hba). Streptodepsipeptide P11B was elucidated as cyclo-(D-Val-L-Lac-L-Val-D- Hiv-D-Val-L-Lac-L-Val-D-Hiv-D-Val-L-Lac-LVal-L-Lac).

Three isolated cyclodepsipeptides were assayed for their activity against the proliferation of four different glioma cell lines (U251, U87-MG, SHG-44, and C6). The results showed that streptodepsipeptides P11A and P11B had potent activity with IC_50_ values of 0.3–0.4 μM and 0.1–1.4 μM, respectively (Table 27), while valinomycin showed much stronger antiproliferative activity with IC_50_ values ranging from 7.6 to 30.0 nM. Doxorubicin had activity with IC_50_ 0.4–3.3 μM. Two new compounds were also tested for growth inhibiting activity in normal human astrocytes, and IC_50_ values were approximately 9.1 μM for P11A and 3.5 μM for P11B. The ratios of IC_50_ for human astrocytes to IC_50_ for glioma cells were within the range of 23–30 for streptodepsipeptide P11A and 3–35 for streptodepsipeptide P11B. Streptodepsipeptide P11A (0.8 μM) blocked U87-MG cell cycle at the G_0_/G_1_ phase; cell population at the G_0_/G_1_ phase was significantly increased by 40.92% after 12 h long treatment in comparison to negative control. Doxorubicin (0.8 μM) as a positive control also had 49.69% DNA increase at G_0_/G_1_ phase. Similar result was also obtained from streptodepsipeptide P11A treated U251 cells. After 72 h treatment streptodepsipeptide P11A (0.8 μM) caused 20.40% increase in the total number of apoptotic cells (early and late apoptotic cells) compared to the control. Moreover, streptodepsipeptide P11A (5 μM or 10 μM for 48 h) significantly downregulated expression of HK2, PFKFB3, GLS, and FASN and slightly downregulated of PKM2. Valinomycin remarkably reduced HK2, FANS, GLS expression levels and slightly affected regulation of PKM2 [137]. These data suggest that targeting multiple tumor metabolic regulators might be an antiglioma mechanism of streptodepsipeptides.

## 7. Steroids and Ergosterols

### 7.1. Steroids

Steroids are organic compounds built in natural sources from isoprenoid precursors. They possess a variety of pharmacological effects including antitumor activity [138].

**Figure 13:**
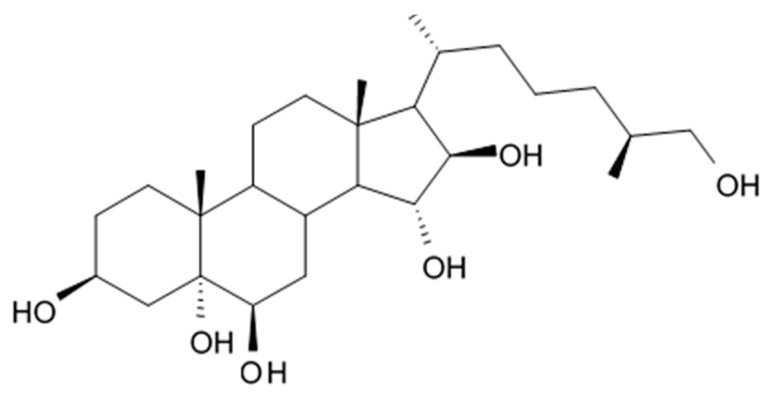
Structure of the steroid with anti-glioblastoma U87MG activity isolated from starfish *Ctenodiscus crispatus* [139].

Five structurally close steroid compounds namely 22E,24ξ)-26,27-bisnor-24-methyl-5α-cholest22-en-3β,5,6β,15α,25-pentol 25-O-sulfate, (22E,24R,25R)-24-methyl-5α-cholest-22-en-3β,5,6β,15α,25,26-hexol 26-O-sulfate, (28R)-24-ethyl-5α-cholesta-3β,5,6β,8,15α,28,29-heptaol-24-sulfate, Δ7-sitosterol, and (25S)-5α-cholestane-3β,5,6β,15α,16β,26-hexaol were isolated from the methanol extract of the starfish *Ctenodiscus crispatus* and investigated for their antiglioma activity [139]. Only the latter compound, which structure is presented on Figure 13, have shown significant dose-dependent cytotoxicity in a range of concentrations 50, 100, and 200 μM against glioblastoma U87MG cells via inhibition of cell growth and apoptosis induction.

After the treatment with anti-glioma steroid, the expression of Bcl-2 proteins was dose-dependently decreased whereas expression of Bax protein was accumulated in increasing concentrations. The ratio of Bax/Bcl-2 exhibited a dose-dependent increase in the U87MG cells. The treatment with this steroid significantly increased the cleavage of caspase-3, caspase-9, and poly (ADP-ribose) polymerase (PARP) in a dose-dependent manner [139]. Thus, anti-glioblastoma effects of active steroids were mediated through the apoptotic process.

### 7.2. Ergosterols

Ergosterols are a class of compounds with tetracyclic skeleton, short alkyl chain, and many hydroxyl groups. Ergosterols are usually found in fungi, marine sponges, corals, and in bacteria [140].

**Figure 14:**
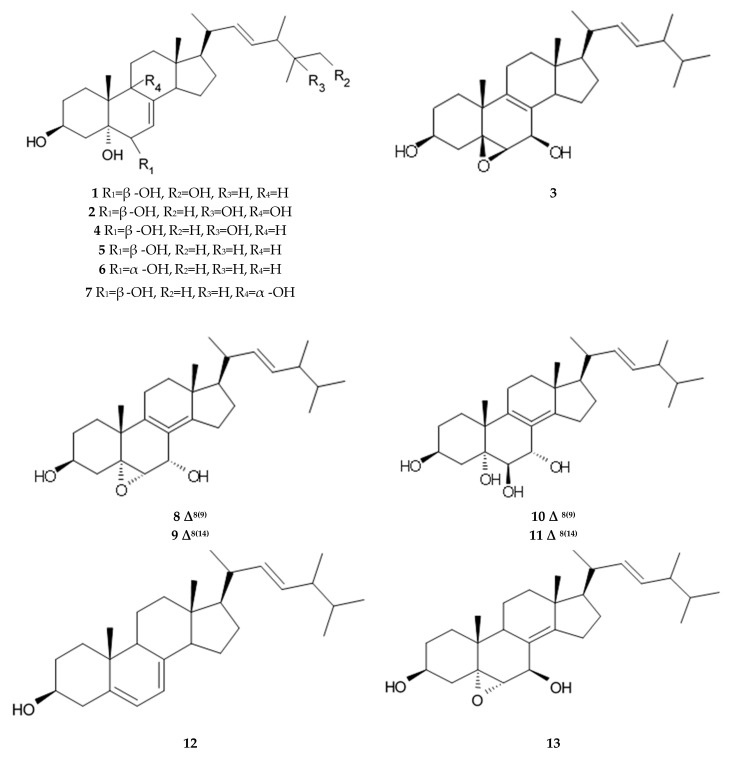
Structures of ergosterols isolated from the culture broth of marine *Streptomyces anandii* H41-59 (reproduced from work [141]).

Three new ergosterols—ananstreps A, B, and C—and 10 previously discovered ones (Figure 14) were isolated from the culture broth of marine *Streptomyces anandii* H41-59 and tested for their cytotoxicity against human glioblastoma cell line SF-268. New ergosterols was defined as ergosta-7,22-diene-3β,5α,6β,27-tetraol (ananstrep A), ergosta-7,22-diene-3β,5α,6β,9α,25-pentol (ananstrep B), and 5β,6β-epoxy-ergosta-7,22-diene-3β,7β-diol (ananstrep C). The rest of compounds were identified as ergosta-7,22-diene-3β,5α,6β,25-tetraol (4), ergosta-7,22-diene-3β,5α,6β-triol (5), ergosta-7,22-diene-3β,5α,6α-triol (6), ergosta-7,22-diene-3β,5α,6β,9α-tetraol (7), 5α,6α-epoxy-ergosta-8(9),22-diene-3β,7α-diol (8), 5α,6α-epoxy-ergosta-8(14),22-diene-3β,7α-diol (9), ergosta-8(9),22-diene-3β,5α,6β,7α-tetraol (10), ergosta-8(14),22-diene-3β,5α,6β,7α-tetraol (11), ergosta-5,7,22–triene-3β-ol (12), and 5β,6β-epoxy-ergosta-8(14),22-diene-3β,7β-diol (13). Major part of already described compounds were isolated from fungi and sponges.

All isolated compounds showed some moderate cytotoxicity against SF-268 cells, whereas Δ8(9)-sterols and ∆8(14)-sterols, namely, ananstrep C, compounds **8**, **10**, and **11** displayed higher efficacies against glioblastoma cells (Table 28) [141]. Active compounds showed cytostatic activity, that was not lower than that of cisplatin belonging to chemotherapeutics. Attention should be given to ananstrep C that bears a Δ8(9) moiety and a rare β-orientation at C-5 and C-6, which are commonly α-orientation in this kind of compounds, indicated much better effect against SF-268.

**Figure 15:**
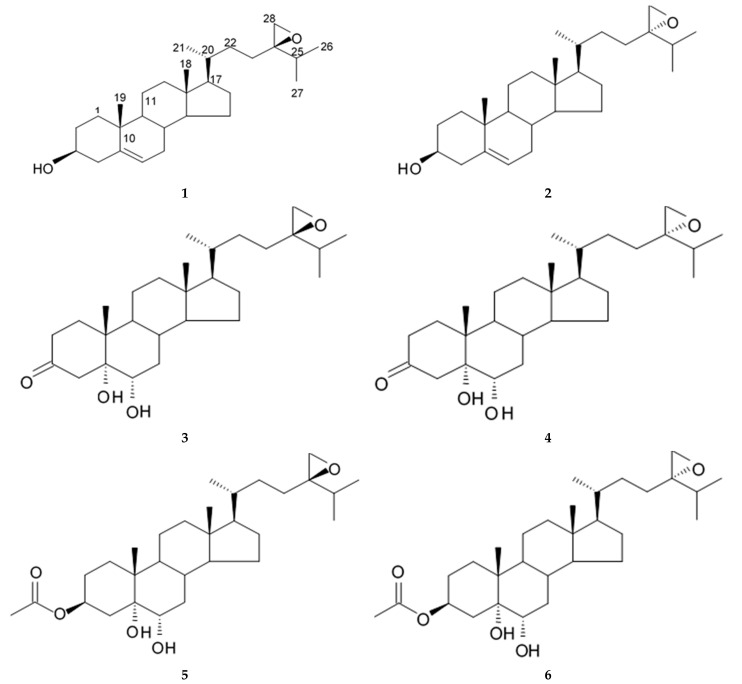
Structures of polyoxygenated 24,28-epoxyergosterols (**1, 2**—new natural products, **3**–**6**—new compounds) from sea anemone *Anthopleura midori* (reproduced from work [142]).

Eleven sterols were isolated from sea anemone *Anthopleura midori* and then identified. First six (1–6) compounds presented rare polyoxygenated ergosterols with a 24,28-epoxy moiety. These six compounds were three pairs of 24 isomers of ergosterols with a 24,28-epoxy group. Epoxyergosterols 1 and 2 were found to be new natural products and 3–6 were new compounds. Compounds **1** and **2** were C-24 isomers of ergosterols with an epoxy moiety at C-24 and C-28. The structures of 1 and 2 were assigned as 24(R),28-epoxyergost-5-en-3β-ol and 24(S),28-epoxyergost-5-en-3β-ol, respectively. Compounds **3** and **4** were also C-24 isomers of 24,28-epoxyergosterols and their structures were elucidated as 24(R),28-epoxyergost-3-one-5α,6α-diol (3) and 24(S),28-epoxyergost-3-one-5α,6α-diol (4). Compounds **5** and **6** were also a pair of C-24 isomers and structures 24(R),28-epoxyergost-3-acetyl-3β,5α,6α-triol and 24(S),28-epoxyergost-3-acetyl-3β,5α,6α-triol, respectively (Figure 15). The rest of the previously known substances were identified as 5α,8α-epidioxyergosta-6,24(28)-dien-3β-ol (7), cholestane-3β,5α,6α-triol (8), holest-5-en-3β-ol (9), ergosta-5,24(28)-dien-3β-ol (10), and ergosta-5,22,24(28)-trien-3β-ol (11).

Compounds isolated from natural sources were assessed for the proliferation inhibiting activity in glioma C6 and U251. Compounds **1**, **2**, **5**, **7**, and **11** exerted dose-dependent activity toward both glioma cell lines. Compounds **3** and **8** inhibited C6 cells proliferation only while **9** and **10** were inactive for both C6 and U251. Epoxyergosterols 1 and 5 were found to be the most active agents regarding C6 cells with an average IC_50_ values 2.41 and 10.58 μM, respectively. Other compounds were less active (Table 29). C6 cells were probably more sensitive then U251 cells. It is interesting that temozolomide exerts its activity towards C6 cells with IC_50_ approximately 69.58 μM (i.e., 29.9. times less active then compound **1** and in 6.6. times les then compound **5**) was not active at all against U251 cells, probably, due to the temozolomide resistance of the U251 cells.

24(R),28-epoxyergost-5-en-3b-ol (1) applied in 40 μM and 80 μM concentration in vitro were found to induce apoptosis and necrosis in U251 cells after treatment for 72 h. In addition, when applied in concentration 80 μM it caused 21.83% increased number of the early apoptotic cells from 0.56% (control) to 22.39%. Portion of the cells in the G_0_/G_1_ cell cycle phase increased by 11.59% and 9.94% after 6 h and 12 h of treatment with compound **1** (80 μM). The alternation occurring in the cell cycle suggests that epoxyergosterols might arrest U251 cells in G_0_/G_1_ phase. Epoxyergosterol 3 (40 μM) also significantly induced apoptosis in C6 cells increasing the number of early apoptotic cells by 44.66% and that of late apoptotic cells by 18.41% [142].

## 8. Antraquinones

One novel antraquinone and two already known ones were extracted from actinomycete *Streptomyces* sp. ZZ406 isolated from the sea anemone *Haliplanella lineata*. A new anthraquinone was elucidated as 1-hydroxymethyl-8-hydroxy-anthraquinone-3-carboxylic acid. Two antraquinones were previously described as 1,8-dihydroxy-3-methyl-anthraquinone (also known as chrysophanol or chrysophanic acid), and 3,8-dihydroxy-1-methyl-anthraquinone-2-carboxylic acid. It has been found that new anthraquinone had a promising activity against different glioma cells, which is comparable with antiglioma effects exerted by doxorubicin (Table 30). At the same time, cytotoxicity of the novel compounds toward normal astrocytes was extremely low with CC_50_ values greater than 100 μM whereas that of doxorubicin is 8.7 ± 1.2 μM. Therefore, selectivity index (CC_50_/IC_50_) of doxorubicin is 0.9 (for U251 cells), 3.5 (for SHG44), and 4.6 (for U87MG), whereas those values for the novel antraquinone were—>17.5, >12.3, and >21.3, respectively. Presented parameters of antiproliferative activity and selectivity index indicate the presence of substantial advantages of the new antraquinone, at least, in comparison to doxorubicin. Already known antraquinones isolated from *Streptomyces* sp. ZZ406 also demonstrated high antiglioma efficacy and activity of chrysophanol was significantly greater than that of doxorubicin [143].

New antraquinone was applied in concentration 30.0 μM and significantly reduced HK2, PFKFB3, PKM2, and LDH5 expression level in the U87MG cells. Based on the results indicating strong activity against glioma cells with extremely high selectivity index and unique antiglioma mechanism the authors of that work assume that the new antraquinone possesses a good potential of antiglioma agent [143].

Marine strain of *Streptomyces* sp. 182SMLY isolated from a sediment sample collected from the East China Sea was found to be a source of two new polycyclic antraquinones, which were elucidated as N-acetyl-N-demethylmayamycin and streptoanthraquinone A. Cytotoxic and antibacterial agent mayamycin was previously isolated from *Streptomyces* sp. strain HB202, a symbiotic bacterium of the marine sponge *Halichondria panicea* [144]. Both anthraquinones remarkably suppressed the proliferation of four different glioma cell lines with IC_50_ values within a range from 0.5 to 7.3 μM (Table 31). Doxorubicin as a positive control exerted its activity with IC_50_ 0.9–9.0 μM. IC_50_ values towards the normal human astrocytes were about 25 ± 1.3 μM for N-acetyl-N-demethylmayamycin and greater than 100 μM for streptoanthraquinone A. IC_50_ ratios between normal astrocytes and glioma cells were 6.4–50 for N-acetyl-N-demethylmayamycin and greater than 14–31 for streptoanthraquinone A. Those values indicate high selectivity index of antraquinones. Also, N-acetyl-N-demethylmayamycin (0.7 μM) and streptoanthraquinone A (3.3 μM) significantly induced apoptosis in the glioma U251 cells: the total number of apoptotic cells (early and late apoptotic cells) were increased by 38.76% and 36.67%, respectively, after 36 h of treatment when compared to control (3.58%) [145].

The two anthraquinone derivatives 1′-deoxyrhodoptilometrin and (S)-(−)-rhodoptilometrin, isolated from the sea lily *Colobometra perspinosa,* exerted moderate anticancer activity in vitro against human glioblastoma cell line SF-268 [146]. Both compounds from the sea lilies *Comanthus* sp. also demonstrated toxic effects against C6 glioma cells (Table 32). The higher cytotoxic activity of 1′-deoxyrhodoptilometrin indicates the relevance of the hydroxyl group at C-1′ position for anti-glioma activity of the quinone structure. 1′-Deoxyrhodoptilometrin, but the (S)-(−)-rhodoptilometrin induced a detected apoptotic cell death via caspase 3/7 activity increase: significant higher enzyme activity was found in C6 glioma cells in 24 h of agitation with concentrations 25 μM and higher. 1′-Deoxyrhodoptilometrin applied in concentration 25 μM also induced necrotic cell death in glioma cells in 24 h agitation period that was detected due to increased LDH activity in the supernatant. (S)-(−)-rhodoptilometrin caused a slight increase in the LDH activity only at the highest concentration used (50 μM). Both compounds did not cause oxidative stress as no increased accumulation of the reactive oxygen species (up to 50 μM) were noted in the C6 cells. They also did not activate the Nrf2/ARE signaling pathway. In addition, both these compounds did not affect transcription factor NF-κB in the H4IIE cell used as a model system that was stimulated by the cytokine TNF-α. Effects of those compounds on the protein kinases involved in different signal transduction pathways associated with cell proliferation (Aurora-A, Aurora-B, CDK2, CDK4, EGF-R, ERBB2, and others), survival (Akt1), angiogenesis (VEGF-R2, VEGF-R3), and metastasis (FAK, MET, SRC) were analyzed. The results demonstrated both antraqionones are highly potent inhibitors of various kinases, in particular, the ones involved into the cell proliferation and angiogenesis [147]. The protein kinases Aurora-A and Aurora-B were inhibited by both compounds with IC_50_ values of 3.0 and 1.81 μM for 1′-deoxyrhodoptilometrin and 4.14 and 4.14 μM for (S)-(−)-rhodoptilometrin, respectively. Aurora kinases are known as potential anticancer drug targets, since they are involved in the control of chromosome assembly and segregation during mitosis.

1′-Deoxyrhodoptilometrin and (S)-(−)-rhodoptilometrin inhibited the VEGF with IC_50_ values of 1.87 and 18.76 μM. That result demonstrates high activity of 1′-deoxyrhodoptilometrin inhibiting mentioned protein kinase 10 times more effectively than (S)-(−)-rhodoptilometrin. Other important protein kinases that were inhibited by 1′-deoxyrhodoptilometrin and (S)-(−)-rhodoptilometrin were cyclin-dependent kinases (regulation of cell cycle), SAK kinase (mitotic regulator), insulin-like growth factor receptor, FAK (cellular adhesion and spreading processes), and EGFR (cell proliferation).

The EGFR family of the tyrosine kinases receptors consists of distinct receptors. EGFR was inhibited by those compounds with IC_50_ values 4.0 µM (1′-deoxyrhodoptilometrin) and 12.4 µM ((S)-(−)-rhodoptilometrin), ERBB-2 was inhibited with IC_50_ values 6.7 µM (1′-deoxyrhodoptilometrin) and 12.1 µM ((S)-(−)-rhodoptilometrin). ERBB-4 was inhibited only by 1′-deoxyrhodoptilometrin with an IC_50_ value 9.4 µM [147]. It is already known that a constitutive activation of the MAPK signaling pathway occurs in many tumors. It was discovered that 1′-deoxyrhodoptilometrin substantially decreased ERK MAP kinase (p44/p42) phosphorylation in the glioma C6 cells despite the whole amount of ERK protein remained unchanged. Therefore, protein kinase inhibition may be one of the main mechanisms contributing antraquinone’s anti-glioma activity.

## 9. Marine Nucleosides: Trachycladines

**Figure 16:**
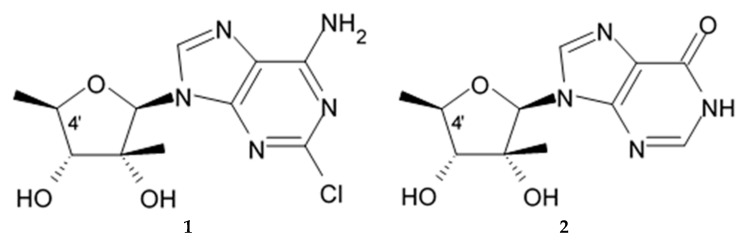
Structures of trachycladine A (**1**) and trachycladine B (**2**).

Naturally occurring modified nucleosides and its synthetic analogues exerting a wide spectrum of biological activity including anticancer effects [148] are considered as a key lead compounds for the drug discovery and development. Initially marine modified nucleosides trachycladines A and B were isolated from the sponges *Trachycladus laevispirulifer* [149] and later from another sponge of the genus *Theonella*. They both contain carbohydrate 2-C-methyl-D-5-deoxyribofuranose part, that was never found in natural sources before, and they differentiate by the nucleic bases (2-chloroadenine for trachycladines A and hypoxanthine for trachycladines B) attached to the carbohydrate part. Trachycladine A represents a carbohydrate-modified analogue of 2-chloro-2′-adenosine, whereas trachycladine B is a carbohydrate-modified analogue of inosine (Figure 16). The first studies revealed a potent in vitro cytotoxic activity of trachycladine A against human colon cancer, leukemia, and breast cancer cell lines with IC_50_ values ranging from 0.3 to 3.0 μM [149]. Trachycladine A exerted significant cytostatic effects on the glioblastoma T98 cell line when applied in concentrations 50 and 100 µM, and against U87 cell in concentrations 10, 50, and 100 µM. U87 cell line was found to be more sensitive to trachycladines. 50 μM concentration of trachycladine A reduced viability of those cells by 70%, and viability of the T98 cells by 50% [150].

**Figure 17:**
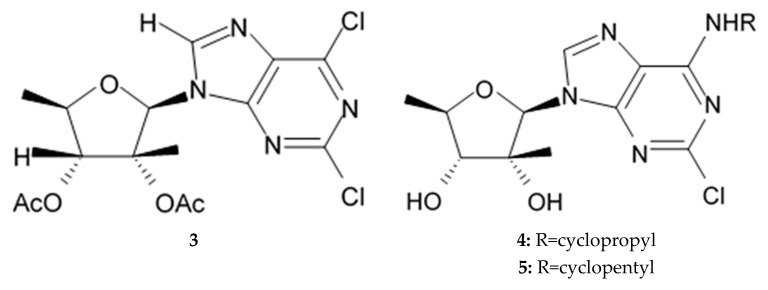
Synthetic analogues of trachycladine A [150].

Two trachycladine analogues, diacetate of the 2,6-dichloropurine derivative (Figure 17, **3**) and N-cyclopropyl trachycladine A (Figure 17, **4**) exerted greater activity against glioblastoma cells.

Comparison of the antiglioma properties of two natural trachycladines showed that trachycladine A definitely is more active compound. Cytotoxicity analysis of several synthetic analogues showed that nucleosides containing chlorine atom at C2 of purine ring are generally more active. On the other hand, N-cyclopentyl analogue of trachycladine A (Figure 17, **5**) demonstrated much lower activity than the corresponding N-cyclopropyl analogue and trachycladine A itself. Diacetate structure is following the same C2 chlorination pattern but it lacks the C-6 amino group, it also proved to be highly cytotoxic, probably due to the better cellular uptake because of its higher lipophilic nature. Additional experiments helped reveal the mode of action of active trachycladines relying more on mitotic catastrophes rather than DNA damage. Their activity as autophagic flux blockers was also postulated [150].

## 10. Glycosphingolipids and Sphingosins

Glycosphingolipids (GSLs) represent a group of biomolecules containing two basic structural units: hydrophobic ceramide moiety and hydrophilic oligosaccharide chain. The ceramide part is made up of a sphingosine moiety composed a long amino alcohol chain with 18–20 carbon atoms and a long fatty acid chain [151].

The cancer-associated GSLs have been considered as the tumor markers, and used as diagnostic markers and targets of cancer treatment [152,153]. On the other hand, bioactive GSLs exert some pharmacological effects—e.g., antimalarial activity—as well as immunomodulating and antitumor activities [154,155].

Various cerebrosides namely glycosylceramides were isolated from marine sponges (Porifera), ascidians (Chordata), octocorals and sea anemones (Cnidaria), and starfishes and sea cucumbers (Echinodermata) [151]. Thus, these marine animals are considered a source of bioactive GSLs.

A bioactive glycolipid fraction obtained from the lipid extract of starfish *Narcissia canariensis* was selected for its ability to significantly inhibit KB (human oral epidermoid carcinoma) cells proliferation. The fraction contained three homologous glycosphingolipids with β-glucopyranoside as a sugar head, 9-methyl-branched 4,8,10-triunsaturated long-chain aminoalcohol as sphingoid base and amide-linked 2-hydroxy fatty acid chains. Their majority (63%) had an amide-linked 2-hydroxydocosanoic acid chain and was identified as the ophidiacerebroside C (firstly isolated from the starfish *Ophidiaster ophidiamus*). The minor components differed by the presence one methylene group were corresponding to ophidiacerebroside B and ophidiacerebroside D. It was found that the final glycolipid fraction demonstrated moderate cytotoxic activity on astrocytoma cells obtained after tumor resection of patients with glioblastoma multiforme-primary culture after 24 h of treatment (Table 33) [154].

Three glycosphingolipids isolated from the marine sponge *Axinyssa djiferi* were named axidjiferosides A, B, and C. They contained an unsaturated long-chain amino alcohol as a sphingoid base. The sugar linked to the ceramide was identified as galactopyranose. The sphingoid base was identified as 2-amino-1,3,4-trihydroxy-octadecene. Generally, axidjiferosides were identified as three homologous β-galactopyranosylceramides composed of 2-amino-(6E)-octadec-6-en-1,3,4-triol and the major one, axidjiferoside A, containing 2-hydroxytetracosanoic acid [155]. These glycosphingolipids in a form of total fraction exerted low cytotoxicity against astrocytoma cells obtained from the tumor resection in patients with glioblastoma multiforme (Table 34).

### Sphingosins

Two modified long-chain sphingoid C18 bases: (2R,3R,6R,7Z)-2aminooctadec-7-ene-1,3,6-triol and (2R,3R,6R)-2-aminooctadec-1,3,6-triol, named halisphingosine A and halisphingosine B, respectively, were isolated from ethyl acetate fraction of methanol extract of the marine sponge *Haliclona tubifera*. Ethyl acetate fraction in contrast to the aqueous and hexane fractions exerted cytotoxic effect in the U87 glioma and SH-SY5Y human neuroblastoma cell lines (Table 35) [156].

Within the recent years, sphingosines are gaining recognition as important signaling mediator of apoptosis. Possible mechanisms involved in sphingosine-mediated cancer cell death are related to its kinase modulating potential, mainly, regarding protein kinase C (PKC), protein kinase A (PKA), and protein kinase Cδ (PKCδ). PKA activation and PKCδ cleaving are associated with regulation of the dimeric 14-3-3 protein function displaying a vital clue of the control pro-apoptotic mediators such as BAD and signal-regulating kinase 1 (ASK-1). Activation of the Jun N-terminal kinases (JNK) is subsequently regulated by ASK-1 [157].

Second suggested mechanism of action may be related to the sphingolipid biosynthetic pathway, which is known to be activated in response factors resulting in accumulation of ceramide and sphingosine in apoptotic cells [157]. Halisphingosines A and B may be a substrate for ceramide synthase and sphingosine kinase resulting in sphingosine converting either into ceramide or sphingosine-1-phosphate (S1P). Ceramide exert antiproliferative effects inducing apoptotic mediators, whereas S1P promotes cell survival and inhibition of apoptosis [158]. Based on the experimental results as well as on knowledge about sphingolipid synthesis and signaling role in debilitation of cell proliferation in U87MG glioma cells [159], authors came to a conclusion that possible cytotoxic mechanism of the sphingosines from *H. tubifera* marine sponge is elucidated by induction of apoptotic mediators and ceramide production. In addition, ethyl acetate fraction was shown to be able to inhibit the production of peroxyl radicals [156].

## 11. Psammaplins

Natural compound named psammaplin A possessing a wide spectrum of biological activities was initially isolated by several independent research teams from marine sponge *Psammaplysilla* (revised to *Pseudoceratina*) sp. or from unidentified sponges back in 1987 [160]. Actually, psammaplin A is found in marine microalgae, cyanobacteria, and in the heterotrophic bacteria living in association with invertebrates (e.g., sponges, tunicates, and soft corals). Psammaplin A was found to have unique symmetrical structure of the disulfide bromine tyrosine dimers with phenol properties and became a starting substance for the whole psammaplin family. Later, psammaplin A was synthesized by Hoshino et al. [161] and that allowed to produce a wide variety of its derivatives and then investigate their antitumor and other activities [162]. Later, psammaplins C, E, F, G, and K were also obtained [163].

Psammaplin A was confirmed to possess antiproliferative effects against various cancer cell lines including triple-negative breast (MDA-MB-231), doxorubicin-resistant human breast (MCF-7/adr), colon (HCT15), ovarian (SK-OV-3), lung (A549, LM4175), bone (BoM1833), endometria, brain (BrM-2a), skin (SK-MEL-2), and central nervous system (XF498) cancer cell lines [162,164]. Cytotoxic effects of psammaplin A are related to the multiple enzyme inhibition such as topoisomerase II, farnesyl protein transferase, mycothiol-S-conjugate amidase, leucine aminopeptidase, DNA polymerase α-primase, aminopeptidase N. In addition, psammaplin A activates peroxisome proliferator-activated receptor gamma (PPARγ) and induces apoptosis in MCF-7 cells [165]. Psammaplin A and its derivatives, psammaplin F and psammaplin G were discovered as highly potent inhibitors of DNA methyltransferase (DNMT) and histone deacetylases (HDAC) playing critical roles in the epigenetic regulation of gene expression [163]. Structural modification with the following investigations of structure–activity relationships demonstrate that disulfide bonds and the oxime moieties are indispensable for the antibacterial and antiproliferative activities of psammaplin A.

Psammaplin A (Sigma Chemical Co., St. Louis, MO, USA) in the in vitro experimental setting suppressed cell viability in the glioblastoma U373MG with IC_50_ values 5 μg/mL after 18 h agitation period (Table 36) [166].

One of the important effects of psammaplin A with high clinical value is related to its influence on the radiosensitivity of the human cancer cells. Investigation of the psammaplin A influence on the radiosensitivity of the glioblastoma U373MG cells have shown that pretreatment with psammaplin A resulted in increased radiosensitization U373MG cells and psammaplin A significantly enhanced radiation induced cell death in U373MG. The dose enhancement ratio (DER) was defined using experimentally adjusted dose necessary for the survival fraction. DER for psammaplin A in U373MG cells was 1.29. It is known that DNMT1, DNMT3A, and DNMT3B are the main functional methyl transferases responsible for setting and maintaining DNA methylation in mammas. Psammaplin A was shown to provoke dramatic reduction of the DNMT1 and DNMT3A expression in the U373MG cells and does not affect DNMT3B expression (at least in a dose 5 μg/mL) [166].

Previously apoptosis was considered as a potential radiosensitization mechanism. Different results were reported indicating the role of apoptosis as a radiosensitizing mechanism induced by DNMT inhibitors. Some studies have shown that combination of radiation and DNMT inhibitor (zebularine) does not increase significantly sub-G1 population of apoptotic cells [167]. On the other hand, DNMT inhibitor (5-aza-2′-deoxycytidine) was demonstrated to induce radiosensitization in the gastric cancer cell line via induction of accelerated rate of apoptosis that was indicated by increased expression of the p53, RASSF1, and DAPK gene expression [168]. Psammaplin A was reported earlier to exert cytotoxic influence on the cancer cells via selective induction of the genes related to apoptosis [168]. This effect of psammaplin A was assumed to lead to the increased radiation induced apoptosis. However, such radiation induced apoptosis was noted in the A549 cell line only and was not found in the U373MG line. That may be explained, at least partially, by the state of the p53 expression. Cell line U373MG contains mutated p53 and may be relatively more resistant to the radiation apoptosis because generally apoptosis is linked to p53 protein. DNA recovery (repair) is another process occurring in the cellular radiosensitizing determination, and DNA reparation activation in the cancer cell after sublethal DNA damage induced by radiation may be one of the resistance factors. Gamma-H2AX been identified as a marker of DNA double-strand break and immune cytochemical analysis with anti-γH2AX-antibodies showed that γH2AX expression zones in cells treated with radiation only goes down in time but can be prolonged (remains unchanged) within 24 h period in the U373MG cells treated with DNMT inhibitor before radiation procedure [166]. These results suggest that inhibition of the DNA damage recovery process is a mechanism that makes a base for radiosesitisizing effects of the DNMT inhibitors in glioma cells. Therefore, psammaplin A possesses a potential to increase radiosensitivity in U373MG glioblastoma cells probably via suppression of the DNA reparative processes.

One of the anti-glioblastoma mechanisms of psammaplins may be related to the induction of the autophagic flux in tumor cells. This phenomenon is of great importance because at present targeting autophagic pathways is thought might play a critical role in designing novel chemotherapeutic approaches in the treatment of human cancers, and the prevention of tumor-derived drug resistance. A number of pharmacological compounds including psammaplin A shown to induce autophagy in various human tumor cells, and some of these compounds affect expression of TP53 protein in cancer cells via cell cycle arrest in the G_0_/G_1_ phase and expression of cyclins [169].

Psammaplin A isolated from the Psammaplinaplysilla sponge decreased cell viability of glioblastoma U87MG cells in a dose-dependent and time-dependent manner with IC_50_ ~7.5 μM for 24 h [164]. This concentration was used for the subsequent molecular analysis of expression of the tumor protein (TP)-p53 family members and their autophagic target genes. Psammaplin A led to a marked increase (4.3 × fold) in the protein levels for TP73α and dramatically induced TP73α phosphorylation (8.7 × fold) that may be a mechanism reducing tumor cell survival and inducing cell death. The present data demonstrate that the autophagy pathway is one of the molecular mechanisms leading to the tumor cell survival modulation [170]. Although autophagy can serve as a pro-survival phenomenon; it often delays tumor cell death via apoptosis, essentially contributing to the demise of tumor cells upon treatment with the anticancer compounds of various origins. TP53 family proteins were found to play a critical role in autophagy signaling [171] and induce the multiple molecular pathways including transcriptional activation of genes targeting autophagic machinery [172]. It was found out that psammaplin A activates transcription of autophagic genes through TP53 family member′s transcriptional function. It upregulates expression levels of ATG5 and UVRAG in U87MG cells and promotes ATG5 and UVRAG promoter activities. Psammaplin A have been demonstrated also to be able to stimulate expression of autophagic proteins involved into autophagy signaling in human glioblastoma tumor cells in vitro. Treatment of U87MG cells with 7.5 μM psammaplin A led to a substantial increase in the ATG5 and UVRAG protein expression [164]. Therefore, psammaplin A has a capacity to upregulate expression of autophagic signaling intermediates in human glioblastoma cells in vitro through a transcriptional regulation by TP53 family members.

Low efficiency of the chemotherapy of the brain malignancies due to the high chemoresistance of the glioblastoma stem cells remains problematic for modern neuro-oncopharmacology. This particular subpopulation of the tumor cells governs tumor initiation and recurrence, they are particularly difficult to be eradicated with chemotherapy. One of the causes of that phenomenon is an elevated expression of P-glycoprotein (Pgp), which is an efflux pump that recognizes chemotherapeutics including temozolomide and other anti-glioma drugs as substrates and pushes them out of the cells [173,174]. Implementation of the direct Pgp inhibitors in combinatorial therapy was rather not successful because of serious adverse events and toxicity. This toxicity is attributed to inhibition of Pgp present in healthy tissues and unexpected drug–drug interactions. Indirect inhibition of Pgp via influencing the carbonic anhydrase activity was thought an alternative approach. Tumor acidosis is known as a hallmark of cancer. Membrane-bound carbonic anhydrases IX (CAIX) and/or XII (CAXII) colocalized with the membrane drug efflux protein, Pgp, in a range of drug resistant cancer cells including glioblastoma, maintain the intracellular/extracellular pH for efficient Pgp activity, and optimal tumor growth, invasion, and metastasis [175]. Nowadays, CAIX- and CAXII-specific inhibitors are considered as potential antitumor agents that indirectly reduce Pgp activity and resensitize solid tumors to Pgp substrates [176].

**Figure 18:**
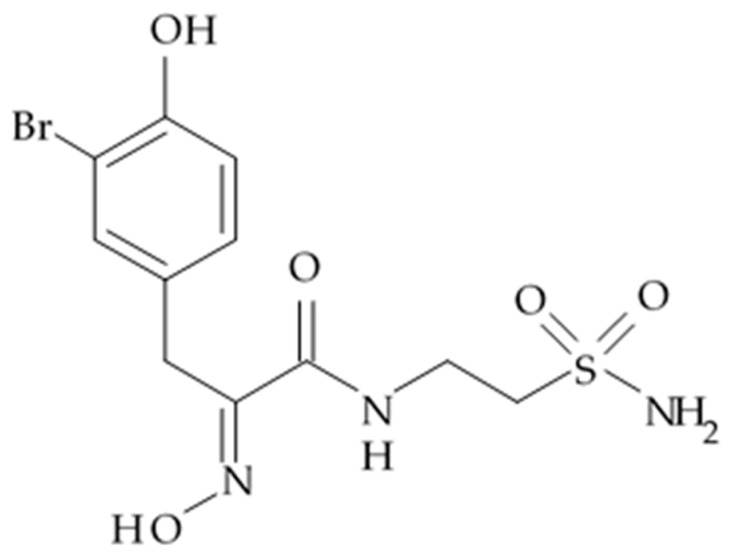
Chemical structure of psammaplin C.

One of the most potent inhibitors of CAIX and CAXII enzymes today is one of the psammaplin A derivatives, namely, psammaplin C isolated from the marine sponge *Pseudoceratina purpurea* in 1991 [177]. The structure of psammaplin C comprises several structural elements: (I) a 3-bromo-4-hydroxy benzylidene moiety; (II) an oxime group; and (III) an aminoethane sulfonamide chain (Figure 18). Alteration of the carbon anhydrase inhibiting activity of psammaplins on the human Ca isoenzyme panel showed that psammaplin C had exceptionally strong inhibition activity for hCA XII, with an inhibition constant (Ki) 0.79 nM. For comparison: acetazolamide, the par excellence therapeutically established CA inhibitor, had shown inhibition activity for hCA XII, with a Ki 5.7 nM [178]. Subnanomolar CA inhibition is uncommon. Moreover, one of the synthesized psammaplin C derivatives, which is a free oxime derived from the thiadiazoyl sulfonamide scaffold, showed very strong inhibition of all the CA isozymes, in particular CA XII with a Ki of 0.56 nM [179].

Experimental studies in mice with orthotopically implanted glioblastoma neurospheres derived tumors (with coexpression of Pgp and CAXII) or xenografts derived from the patients showed that co-therapy of temozolomide with a CA XII inhibitors may more effectively affect glioblastoma by suppressing an important temozolomide resistance mechanism. CA XII inhibitors themselves did not affect tumor growth in the mouse model studies and did not prolong life span of experimental animals, but significantly enhanced efficacy of temozolomide. Simultaneously, application of the CA XII inhibitors was particularly effective against the glioblastoma with high anti-TMZ resistance. The combination of TMZ with CA XII inhibitors rescued the antiproliferative and proapoptotic effects of TMZ, as verified by reduced intratumor-positive immunostaining for Ki67 and increased activation of caspase-3 [175,179]. Thus, although TMZ alone reduced Pgp expression and activity in neurospheres, this reduction was not sufficient to yield antitumor efficacy in vivo. The greater inhibition of Pgp, as achieved by combining the potent CA XII inhibitor (psammaplin C derivative) with TMZ, was however able to restore the intracellular cytotoxic levels of TMZ and facilitate enhanced drug efficacy. It should be emphasized that psammaplin C shows no toxicity and is only effective if used in combination with a chemotherapy [175].

Therefore, a new combination therapy, based on a CA XII inhibitor with TMZ may be considered as a potentially viable clinical tool to overcome Pgp-mediated TMZ-resistance in glioblastoma stem cells as well as more effective therapy of the brain tumors [179].

## 12. Xyloketals

**Figure 19:**
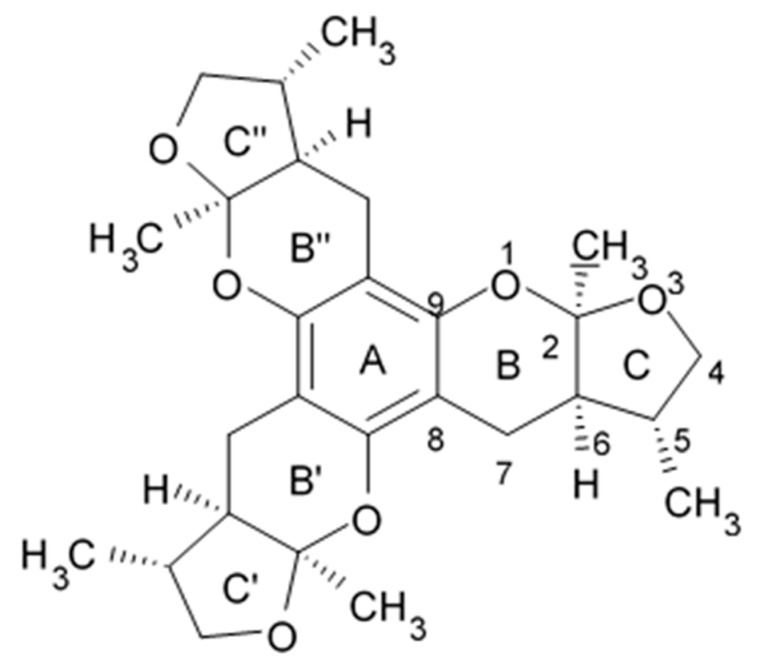
Structure of xyloketal A from the mangrove fungus Xylaria sp. [180].

The xyloketal group of natural compounds was isolated from the mangrove fungus *Xylaria* sp. This group of related ketals is structurally unique [180]. Xyloketal A has C3 symmetry with a cis-junction between the tetrahydropyran and tetrahydrofuran rings (Figure 19), the other members are missing axial symmetry. The methyl groups at C-5 of C rings are also oriented syn to other methyl groups at C-2 placed between the oxygens in the spiroketal functions. The angular skeleton of xyloketal B, a bis adduct analogue of the tris adduct xyloketal A, is more stable than the linearly condensed xyloketal C, which spontaneously rearranges in solution to the more stable angular structure of xyloketal B. Xyloketal D is an acetylated mono adduct structure, and xyloketal E is a tetrahydrofuran-linked angular bis adduct related to xyloketal B. Some studies reported that xyloketal B exerts several bioactive functions including anti-stress and anti-ageing, neuroprotective effects, and antioxidant activity [181].

24 h long treatment with xyloketal B in various concentrations of (from 31.25 to 1000 μM) reduced U251 cell viability in a concentration-dependent manner. Cell viability was significantly decreased by 85.4% ± 2.9%, 61.4% ± 4.3%, 12.2% ± 2.6%, and 1.3% ± 0.1% in comparison to control for 125, 250, 500, and 1000 μM xyloketal B concentration, respectively (*p* < 0.05). Nonlinear curve fit was carried out to evaluate the dose–response of xyloketal B with IC_50_ 287.1 ± 1.0 μM (Table 37). Xyloketal B applied in the concentration range 37.5–300 μM exert sell proliferation inhibiting activity depending on the period of application and concentration. It should be emphasized that xyloketal B in concentrations <300 μM generally exerted only cell proliferation inhibition activity but not a cytotoxic effect. In concentration greater than 300 μM xyloketal B significantly inhibited cell migration in U251 line [182].

Experiment devoted to investigation of the mechanisms providing antiproliferative activity xyloketal B have shown that p-Akt and p-ERK1/2 protein expression in the U251 cells was significantly decreased after xyloketal B treatment in concentration 300 μM for 24 h. Therefore, reduced cell viability, proliferation, and cell migration in glioblastoma is caused by suppression of the signaling pathways PI3K/Akt and MEK/ERK as well as by the TRPM7 current blockade without alteration of the TRPM7 protein expression in glioma cells. Authors suppose that the marine compound xyloketal B may be a promising drug candidate for anti-glioblastoma therapy [182].

## 13. Pheophorbide *a*

Pheophorbide α is a typical representative of the organic compound class tetrapyrroles, which as suggested by the name, consists of four pyrrole-derived substances linked either in linear or cyclic fashion via methine bridges. There are well known and studies members in this class of molecules such as hemes giving blood its red color and the chlorophylls, which are responsible for the green color of plants, algae, and some bacteria, as well as cobalamin, siroheme, coenzyme F430, heme d1, and the photopigments bilins [183]. Molecular formula of pheophorbide a is C_35_H_36_N_4_O_5_ and its IUPAC name is (3S,4S)-9-ethenyl-14-ethyl-21-(methoxycarbonyl)-4,8,13,18-tetramethyl-20-oxo-3-phorbinepropanic acid. Pheophorbide a purified from an edible red seaweed *Grateloupia elliptica* was shown to exhibit strong anticancer activity with no direct photo-irradiation against various cancer cell lines including mouse melanoma cells (B16-BL6), human epithelial carcinoma cells (HeLa), human cervical cancer cells (SiHa), and human ovarian cancer cells (SK-OV-3) with IC_50_ values 18.3 ± 2.9, 9.5 ± 2.3, 13.2 ± 2.6, and 7.0 ± 2.0 μg/mL, respectively. However, pheophorbide a exerted the strongest anticancer effect against U87MG glioblastoma cells that was comparable with activity of the positive control drug paclitaxel [184]. Cytotoxic effect of pheophorbide a was not observed in the normal HUVECs whereas the positive control paclitaxel was found to demonstrate significant and notable cytotoxic activity (Table 38).

The glioblastoma growth inhibition activity exerted by pheophorbide a was related to the cell cycle arrest in the G_0_/G_1_ phase and apoptosis as well as with genomic DNA degradation. Pheophorbide a specifically inhibited only growing U87MG cells but did not affect resting ones. The results obtained through the studies suggest that pheophorbide a isolated from *G. elliptica* could be a good source of glioblastoma-specific therapeutics with no clinically notable side effects [184].

## 14. Phorboxazoles

In 1995, two unique compounds named phorboxazole A and its C13 epimer phorboxazole B were isolated from the marine sponge Phorbas sp. Their complex chemical structure had given a base to consider these phorboxazoles as a new class of natural products containing an unprecedented array of oxane, oxazole, macrolide, and polyene moieties. In addition to the pronounced antifungal activity against *Candida albicans* shown previously in the in vitro experiments, phorboxazoles were reported to provide extremely high cytostatic effects towards the National Cancer Institute’s panel of 60 tumor cell lines with the mean GI_50_ values less than 1.6 × 10^−9^ M [185]. Later, a complete synthesis of phorboxazole A was performed [186] that gave a start to a series of studies devoted to fabrication of the synthetic phorboxazole derivatives. Thus, phorboxazoles were suggested to be the most potent natural cytostatic agents that are discovered to date [187].

Experimental studies comparing antiproliferative activity of the phorboxazole derivatives against human glioblastoma cell line U373 have shown these tumor cells in a concentration-dependent fashion with low nanomolar IC_50_ values were inhibited by synthetic phorboxazole A as well as its analogues 45,46-dehydrobromo-phorboxazole A bearing an alkyne part in C45–C46 position of the terminal bromide, and 33-O-methyl-phorboxazole A containing mixed methyl ketal instead of the C33 positioned hemiketal (Table 39). IC_50_ values for other synthetic phorboxazole analogues, especially, for 32-methyl-phorboxolide A (C1–C32 analog), 31-methyl-phorboxylate (C31–C46 analog), 29-phorboxamide A (hydrated analog), C1–C38 of phorboxazole A, and 18-methyl-phorboxylate (C18–C46) were experimentally shown to be greater than 2 mM.

Structure–activity relationship studies demonstrated that just simple modifications like replacement of the terminal vinyl bromide of phorboxazole A with an alkyne, or C33 hemiketal with a mixed methyl ketal did not lead to substantial loss of anticancer activity. Neither C1–C32 macrolide-containing domain, nor C31–C46 side-chain portion were separately sufficient to sustain the potent anticancer activity of phorboxazole A. The simple covalent joining of the macrolide and side-chain parts of phorboxazole A via an amide at C29–C31 instead of the planar vinyl-substituted oxazole at this position of phorboxazole A was not sufficient to regain appreciable activity. Maintaining the central oxazole but truncating the side chain by omission of the lipophilic C39–C46 polyene domain resulted in abolished antitumor activity. Deletion of the C2–C17 portion of the phorboxazoles containing oxazole, acrylate, and bispyran moieties similarly resulted in activity loss. These findings suggest that macrolide, central oxazole, and polyene side chain portions of phorboxazoles are necessary for a potent anticancer activity [187].

## 15. Phlorotannins

Phlorotannins are the tannin derivatives existing in a form of polyphenols that built as a result of the phloroglucinol unit polymerization. The most widely known phlorotannins are phloroglucinol, eckol, dieckol, 8,8′-bieckol, 6,6′-bieckol, dioxinodehydroeckol, phlorofucofuroeckol, and a few others [188].

**Figure 20:**
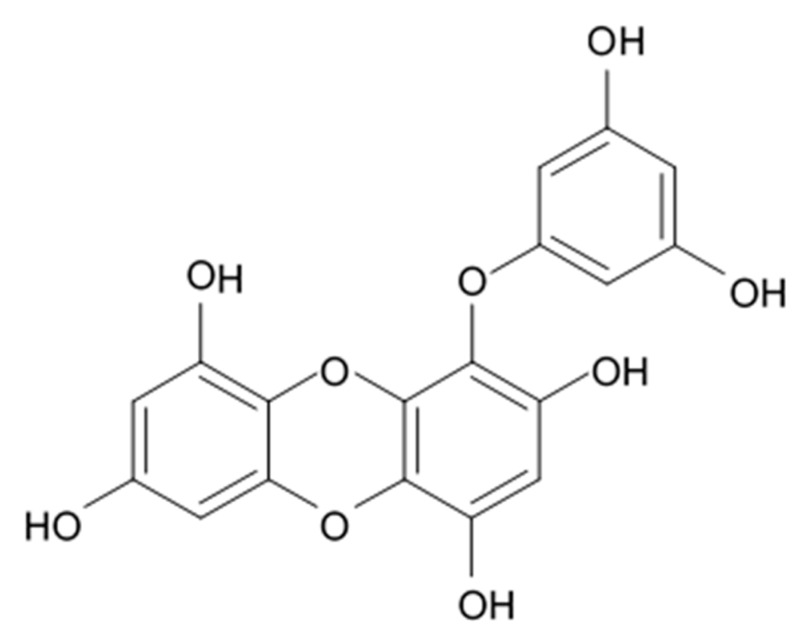
Structure of eckol.

Eckol is a precursor compound illustrating the dibenzo-1,4-dioxin class of phlorotannins and containing phloroglucinol components linked to each other in multiple fashion (Figure 20). Eckol is known to be produced in several marine organisms, in particular, in brown algae including *Ecklonia cava* (Laminariaceae), and red algae [189].

Hyun et al. [190] investigated the effect of eckol on stem cells and malignancies in glioma stem-like cells. The study was performed with the use of glioma cancer cell lines U87MG and U373MG. Patient-derived glioma stem-like cells X01GB and X03AOA were established from acutely resected human tumor tissues. The X01GB line was derived from a patient with a glioblastoma multiforme, and X03AOA was derived from a patient with anaplastic oligoastrocytoma.

It was shown before that glioma subpopulation expressing CD133 protein is enriched in cancer stem-like cells showing greater tumorigenic potential than CD133 negative cells. In a similar way to neural stem/progenitor culture, those glioma stem-like cells expressing CD133 can be enriched in a serum-free medium supplemented with growth factors, where glioma cell fraction continue to proliferate and form spheres instead of a monolayer [191]. Treatment of the sphere-cultured glioma cells with eckol in concentrations from 50 to 90 μM also decreased the expression of the glioma stem-like cell markers CD133, Nestin, and Musashi-1. Although cell death was not significantly triggered by the eckol treatment at the concentration range 10 to 80 μM, there was an increase of cell kill rate noted after application of eckol in concentration 90 μM. Eckol treatment led to a marked reduction of the Sox2 levels in glioma-initiating cells. Sox2 is a transcription factor essential for maintaining the self-renewal of several types of undifferentiated stem cells, in particular, neural stem cells. Sox2 is also key component for maintaining self-renewal capacity of the brain cancer stem cells [192]. Eckol suppressed expression of Notch2 and β-catenin in the cancer stem-like cells in several human cancers. Results of the in vivo experiments showed that eckol treatment contributes to marked tumor growth inhibition in xenograft mice [190]. Importantly, eckol treatment also effectively reduced the resistance of the glioma stem-like cells to ionizing radiation and temozolomide.

It is also known that the key signaling pathways PI3K-Akt and Ras-Raf-1-Erk activated in cancer stem-like cells are involved in the survival and maintaining the stemness in cancer stem-like cells. PI3K-Akt pathway contributes to the resistance of cancer cells to ionizing radiation as well [193]. Hyun et al. have discovered that eckol treatment effectively inhibits both PI3K-Akt and Ras-Raf-1-Erk pathways in the glioma stem-like cells. Treatment with eckol was also noted to induce a marked suppression of the PI3K and Akt activities, and completely inhibit Ras-Raf-1 interaction and Raf-1 and Erk activations in the sphere-forming glioma stem-like cells. On the base of presented results, authors made a conclusion that eckol may enhance the sensitivity of glioma stem-like cells to anticancer therapies such as ionizing radiation or chemical drugs via inhibition of PI3K-Akt and Ras-Raf-1-Erk pathways [190].

The synthesized phloroglucinol derivative 2,4-bis(4-fluorophenylacetyl)phloroglucinol (BFP) induced cell death and slowed proliferation rate in three glioma cell lines U251, U87, and C6 in a concentration-dependent manner. At the same time, it did not affect primary human astrocytes (Table 40) [194].

Treatment with BFP (3 µM) for 4 h induced a nuclear shrinkage and nuclear condensation. Nuclear fragmentation was noted after 8 h of treatment. BFP also induced concentration-dependent sub-G_1_ arrest in U251 glioma cells. Furthermore, treatment of U251 cells with BFP induced significantly upregulated Bax protein expression but did not affect expression of Bcl-2 protein. BFP application also increased procaspase-3 degradation and caspase-3 cleaved form expression in glioma cells. Upstream procaspase-9 also degraded, and cleaved-caspase-9 increased upon BFP treatment in U251 cells. BFP also increased cleaved-PARP expression time-dependently. Treatment of U251 glioma cells with BFP induced generation of the reactive oxygen species (ROS) and also increased expression of the endoplasmic reticulum (ER) stress markers such glucose-regulated protein (GRP)-78, GRP-94, IRE1, phosphorylation of eukaryotic translation initiation factor 2 alpha (eIF-2) and induced upregulation of the CAAT/enhancer-binding protein homologous protein (CHOP). In addition, BFP treatment provoked down-stream caspase activation such as pro-caspase-7 and procaspase-12 degradation suggesting the induction of ER stress [194]. These results indicate that the phloroglucinol derivative BFP induces glioma cell death via mediation of the ROS generation, which subsequently enhances GPR78 and CHOP expression, increases activity of caspase-9 and caspase-3 leading to apoptosis.

## 16. Carotenoids

Carotenoids along with chlorophylls and phycobiliproteins build a large group of organic pigments. They represent a class of tetraterpene pigments commonly found in bacteria, fungi, algae, as well as high plants and animals. The most known and studied carotenoids include fucoxanthin, astaxanthin, sifonaxanthin, violaxanthin, neoxanthin, β-carotene, capsanthin, lutein, and others [195,196].

**Figure 21:**
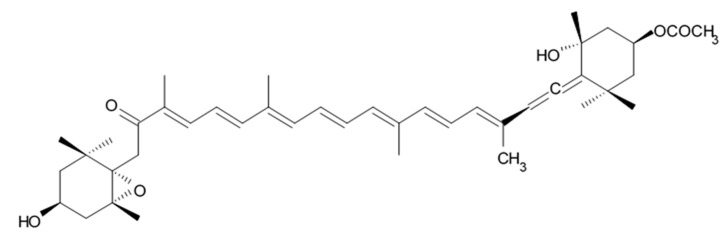
Chemical structure of fucoxanthin derived from marine brown algae.

Fucoxanthin is an orange pigment possessing unique structure that contains allenic bond, conjugated carbonyl, 5,6-monoepoxide, and acetyl groups (Figure 21) [197]. This compound is produced by both microalgae Bacillariophyta and macroalgae Phaeophyceae. Besides, it can be extracted from almost all photosynthetic and nonphotosynthetic organisms such as bacteria and fungi [198].

Seaweeds are rich with fucoxanthin, which is considered as effective anticancer, antioxidant, antiangiogenic, antidiabetic, antiobese, anti-inflammatory, and photo-protection pharmacological agent due to its powerful antioxidant properties [198]. Antiproliferative and antiapoptosis effects of fucoxanthin were confirmed in the human cancer cell lines including hepatic carcinoma HepG2, gastric adenocarcinoma MGC-803, and non-small-cell lung cancer cells [199].

Regarding the effects of fucoxanthin on the glioma cell growth, it was shown that fucoxanthin in concentrations 25, 50, 75, and 100 µM significantly reduced cell viability in the glioma U87 and U251cell lines in dose-dependent and time-dependent manner, but did not affect viability of healthy neurons. Applied in concentrations 25 and 50 µM it induced apoptosis due to the disruption of mitochondrial potential (∆ψm) and destroyed mitochondrial function. It was also found that fucoxanthin provides an increment in Bax expression and a decrement in Bcl-2 expression in both U87 and U251 cells [200]. The balance of Bcl-2/Bax dominates the switch which turns on or off the apoptosis. Enhanced activation of cleaved-PARP, caspase-9 and caspase-3 was noted in the fucoxanthin-treated tumor cells. Electron microscopy studies had confirmed that fucoxanthin promotes cell death by induction apoptosis via mitochondrial pathway. It was further figured out that expression of phosphorylation in Akt and mTOR was dramatically decreased by fucoxanthin treatment of the glioma cells in concentration of 25 and 50 µM. Moreover, the level of phosphorylated mTOR in the glioma cells was notably reduced at higher concentration of fucoxanthin. The expressions of active Akt/mTOR form were lessened due to the glioma cell exposure with fucoxanthin or PI3K inhibitor, LY294002, individually.

In was shown in the scratch wound healing assay and trans-well assays that fucoxanthin can inhibit invasion and migration rates of the human glioblastoma cells. It was found out also that fucoxanthin possess a capacity of human glioblastoma cell inhibition and the protein levels of MMP-2 and MMP-9 was significantly suppressed after incubation of glioma cells with fucoxanthin at the concentration of 25 and 50 µM. The degree of the phosphorylated-p38 expression was significantly decreased in a concentration-dependent manner after treatment of the cells with fucoxanthin. However, fucoxanthin abnormally increased the protein levels of phosphorylated ERK and did not affect expression of p-JNK/JNK. In addition, the MMPs was significantly reduced by the p38 inhibitor (SB203580) treatment. Results of the tumor xenograft studies in the immune compromised nude mice (BALB/c-nude) used as a test model had shown that tumor volume and weight were obviously lowered that demonstrate the tumor growth inhibiting capacity of fucoxanthin in U87 cells. Such molecules as p-Akt, p-mTOR, p-p38, MMP-2/9, and BCL-2 were all downregulated, whereas Bax and cleaved-caspases-9 were upregulated after fucoxanthin treatment of the tumor tissues. These findings were consistent with in vitro results [200]. Therefore, there are convincing experimental evidences indicating that fucoxanthin exerts antiglioma activity, promotes apoptosis via PI3K/Akt/mTOR pathway inhibition and suppresses tumor invasion and migration due to the restriction of the p38/MMPs signaling pathway in human glioblastoma cells. Authors of the research studies suppose that fucoxanthin may represent a new anticancer drug prototype that can damage glioma cells and prevent metastasis. It may find its way as an emerging therapeutic agent in the future.

### Hydratoperidinin

Carotenoid hydratoperidinin isolated from the sea anemone *Anthopleura midori* showed moderate antiproliferative activity against rat glioma C6 and human glioma U251 cells [111]. Antitumor activity of hydratoperidinin was significantly higher than temozolomide activity (Table 41).

## 17. Conclusions

Quantitative analysis of the data presented in the previous sections of this review shows that more than 100 structurally diverse marine natural compounds possess significant capacity of suppressing proliferative activity of the various glioma cell lines and often even reduce their viability. Some of those compounds are capable to inhibit activity of the cancer cells themselves as well as the tumor stem cells. Authors of the experimental investigations suppose that those compounds may be considered as prototypes for the development of novel anti-glioma pharmaceuticals. In the most studies average inhibiting concentrations of the tested compounds under in vitro conditions were comparable with cytotoxic concentration of antitumor drugs including temozolomide, which is the first line drug of the standard antiglioblastoma chemotherapy. A group of compounds such as ecteinascidin-770, renieramycin M, papuamine, actinomycins D, V and X0β, fradimycin B, valinomycin, streptodepsipeptides P11A and P11B had demonstrated extremely high cytotoxicity inducing glioma cell death in the nanomolar concentration range where half inhibiting concentrations of the officinal antitumor drugs are usually in the range between a few micromoles and several dozen micromoles. For example, IC_50_ for temozolomide is approximately 85.8 and 69.5 µM for the glioma cell lines SHG-44 and C6, respectively, and more than 100 µM for U87-MG and U251 cells [113]. At the same time, phorboxazoles isolated from marine sponges and exerted anti-glioma effects in nanomolar concentrations have a property of the most potent cytostatic agents among all pharmaceuticals studied so far [187]. Despite cytotoxic activity is not the only parameter of the anticancer potential of chemical compounds, we would like to point out that the substances with the IC_50_ values greater than 100–150 µM rather do not have a perspective as the potential anti-glioma drugs.

Important factor for the successful development of natural compounds towards novel anti-glioma agents is an index of selectivity degree of compounds regarding cancer cells. Usually, it is expressed as a ratio of IC_50_ for the normal cells (for example, for astrocytes, fibroblasts, or HUVECs) to the IC_50_ for glioma cells. Unfortunately, this parameter was provided only in few numbers of publications. Nevertheless, it should be mentioned that such compounds pheophorbide a isolated from read algae [184], triterpene and steroid glycosides from the starfish and holothurians [103,110], sesquiterpene aplysin discovered in gastropod molluscs [120], anthracycline antibiotic SZ-685C from marine fungi [97], as well as macrolide antibiotics flavofungin II and spectinabilin isolated from marine streptomyces bacteria [102] demonstrated substantial inhibition of the glioma cells along with mild toxic influence on the normal cells. At the same time, a series of compounds such as, for example, meriolins and fascaplysins apart of the extremely strong antiglioma activity exert pronounced toxic effect on the normal cells [51,64]. Structures of such compounds can be considered only as a base for further discovery of the new agents that would be less toxic for the normal cells.

Results of analysis of the taxonomical diversity of the marine species that were used as a source of the compounds with anti-glioma activity pose some interest (Table 42). Despite an enormous list of those compounds, about half of the active compounds were isolated from a few actinomyces bacterial strains belonging to the one genus Streptomyces (about 40 compounds) and from marine sponges (about 20 compounds) belonging to the class Demospongiae (phylum Porifera). Also, significant number of those compounds were isolated from star fish (class Asteroidea), sea cucumbers (class Holothuroidea) and ascidians (phylum Chordata, subphylum Tunicata). Compounds exerting anti-glioma activity in small amounts was found in cyanobacteria, marine fungi, red and brown alga, actinians (sea anemones), crustaceans, and mollusks. Therefore, now we have a substantially limited number of taxons that were marked with the presence of anti-glioma activity. That does not mean that the further search of active compounds should be limited with only those taxons. That is the opposites, taking to account the fact that taxonomical diversity of the marine species is substantially greater than the terrestrial species diversity. Also, chemical diversity of the marine compounds is probably exceeding the one of terrestrial species. Therefore, it would be reasonable to expand the search of the anticancer agents in representatives of other marine taxons.

## Figures and Tables

**Table 1:** Structure and inhibitory activity of marine metabolites from ascidian *Polyandrocarpa zorritensis* against glioma C6 line [39].

Compound	Structure	IC_50_, µM *
Zorrimidazolone	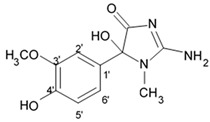	155 ± 13
3-indolylglyoxylic acid	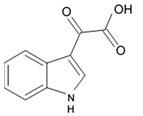	314 ± 17
3-indolylglyoxylic acid methyl ester	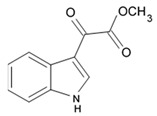	305 ± 15
Methyl 2-(4-hydroxy-3-methoxyphenyl)-2-oxoacetate	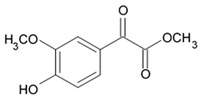	>10^3^

* IC_50_ values are expressed as mean ± SEM.

**Table 2:** Structure and cytotoxic effects of meriolins against glioma cell lines [51].

Compound	Structure	IC_50_ (nM)	
		SW1088	U87
Meriolin 3	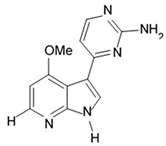	34	76
Meriolin 5	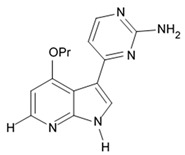	32	18.4
Meriolin 15		46	5.1

**Table 3:** Structure and cytotoxic activity of fascaplysin against glioma C6 cells [64].

Compound	Structure	IC_50_ (µM)
Fascaplysin	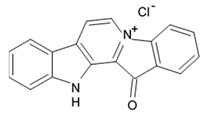	≈1.0 µM

**Table 4:** Anti-glioma activities of α-carboline derivatives against glioma cells in vitro [69].

α-Carboline Derivative	Structure	IC_50_ (μM)			
		U87	T98G	U251	C6
	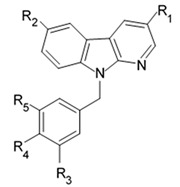				
TJY-13	R_1_=H; R_2_=C(=O)CH_3_; R_3_=H; R_4_=H; R_5_=H	>10	>10	>10	>10
TJY-14	R_1_=H; R_2_=C(OH)CH_3_; R_3_=H; R_4_=H; R_5_=H	>10	>10	>10	>10
TJY-16	R_1_=H; R_2_=C(=O)CH_3_; R_3_=OCH_3_; R_4_=OCH_3_; R_5_=OCH_3_	0.042	0.043	0.088	0.050
TJY-18	R_1_=H; R_2_=H; R_3_=OCH_3_; R_4_=OCH_3_; R_5_=OCH_3_	>10	>10	>10	>10
TJY-22	R_1_=CH_3_; R_2_=H; R_3_=OCH_3_; R_4_=OCH_3_; R_5_=OCH_3_	>10	6.222	7.643	8.842
TJY-24	R_1_=H; R_2_=CH_3_; R_3_=OCH_3_; R_4_=OCH_3_; R_5_=OCH_3_	7.769	1.591	6.314	7.078

**Table 5:** Anti-glioblastoma activities of tetrahydroisoquinoline alkaloids against human glioblastoma cells U373MG [72].

Compound	Structure	IC_50_ (nM)
	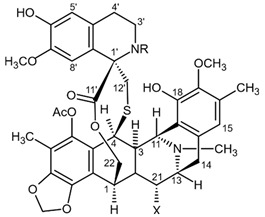	
Ecteinascidin-770	R=H, X=CN	4.83
2′-N-4″-pyridinecarbonyl derivative of ET-770	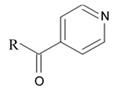	1.70
Renieramycin M	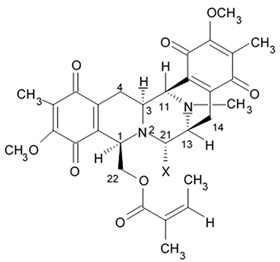	3.10

**Table 6:** Structure and antiproliferative activities of C2-methyl-7-deazahypoxanthines against U-87 glioma cells [79].

Formula	Structure	GI_50_ (μM)
C_20_H_15_N_3_NaO_2_	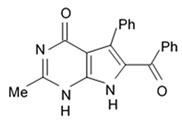	0.077 ± 0.002
C_20_H_15_ClN_3_O_2_	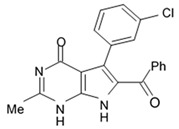	0.90 ± 0.16
C_20_H_14_BrN_3_NaO_2_	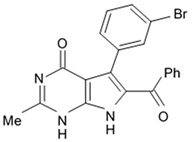	0.94 ± 0.12
C_20_H_14_FKN_3_O_2_	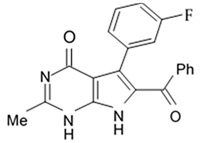	1.7 ± 0.1
C_19_H_14_N_4_NaO_2_	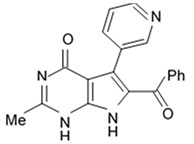	9.23 ± 2.13
C_19_H_14_BrN_4_O_2_	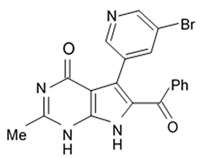	2.4 ± 0.6
C_20_H_14_Br_2_N_3_O_2_	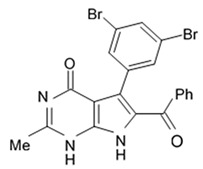	0.72 ± 0.06

**Table 7:** Anti-glioma activity of pyrrospirones C–J and penicipyrroether A isolated from the cultures of marine-derived fungus *Penicillium* sp. ZZ380 against the proliferation of glioma cells [80,81].

Compound	IC_50_ (µM)			
	U87MG	U251	SHG44	C6
Pyrrospirone C	12.15 ± 0.61	22.12 ± 1.11	10.03 ± 0.10	13.87 ± 1.26
Pyrrospirone D	9.95 ± 0.50	23.39 ± 1.27	13.74 ± 0.55	14.56 ± 0.95
Pyrrospirone E	16.24 ± 0.68	26.64 ± 2.14	15.76 ± 1.20	21.03 ± 3.15
Pyrrospirone F	12.44 ± 0.81	22.82 ± 1.15	8.93 ± 0.76	14.87 ± 1.66
**Pyrrospirone G**	**1.06 ± 0.05**	**1.28 ± 0.06**	**2.14 ± 0.11**	**8.52 ± 1.01**
Pyrrospirone H	12.89 ± 0.64	23.92 ± 1.20	13.02 ± 0.79	23.24 ± 2.24
Pyrrospirone I	13.67 ± 0.53	13.46 ± 0.76	7.44 ± 0.97	19.18 ± 2.11
Pyrrospirone J	10.52 ± 0.62	17.92 ± 0.93	n.d. *	n.d.
**Penicipyrroether A**	**1.64 ± 0.05**	**5.50 ± 0.12**	n.d.	n.d.
Doxorubicin	1.20 ± 0.06	8.03 ± 1.20	0.67 ± 0.10	0.47 ± 0.10

*, no data. The bold was used to indicate the promising agents.

**Table 8:** Structure and anti-glioma activity of pseurotin A isolated from marine bacterium *Bacillus sp*. FS8D [83].

Compound	Structure	IC_50_ (μM)			
		C6	U251	SHG-44	U87-MG
Pseurotin A	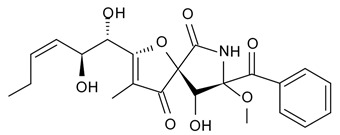	0.51	4.9	5.8	29.3
Doxorubicin		0.5	9.6	2.5	1.9

**Table 9:** Structure and cytotoxicity activity of polycyclic diamine alkaloids from marine sponge *Neopetrosia* cf. *exigua* against glioblastoma SF-295 cells [86].

Compound	Structure	IC_50,_ μM
Papuamine	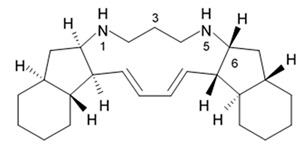	0.8
Haliclonadiamine	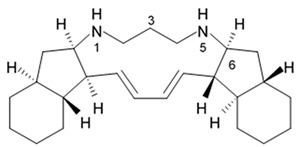	6.3
Neopetrocyclamine A	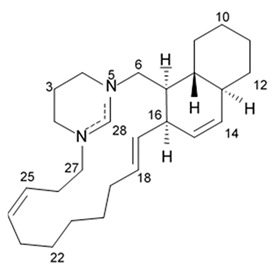	>20
Neopetrocyclamine B	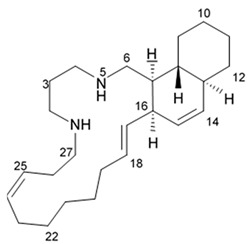	>20

**Table 10:** In vitro inhibitory concentrations of granulatimide, isogranulatimide from ascidian *Didemnum granulatum* and amino analogues against glioma cell lines [89].

Compound	Structure	IC_50_ (μM)	
		HS683	U373
Granulatimide	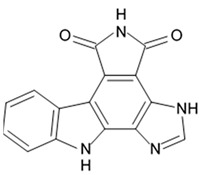	23	6
Isogranulatimide	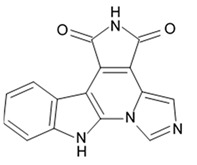	>100	>100
Amino analog 1	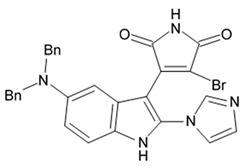	6	3
Amino analog 2	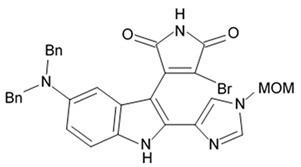	3	3

**Table 11:** Structure and antiproliferative activity of actinomycins D, V, and X_0β_ isolated from *Streptomyces* sp. ZZ338 against glioma cells [94].

Compound and Structure	IC_50_		
	U251	SHG44	C6
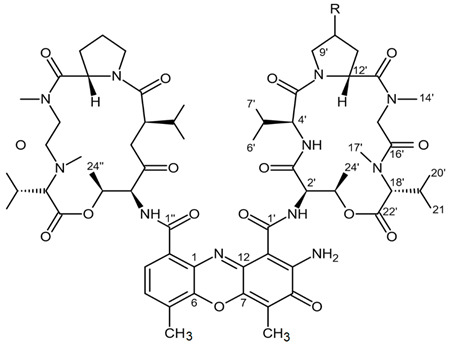			
Actinomycin D (R = H)	10.06 ± 0.6 nM	3.31 ± 0.25 nM	1.01 ± 0.05 nM
Actinomycin V (R = O)	1.80 ± 0.19 nM	1.37 ± 0.07 nM	0.42 ± 0.23 nM
Actinomycin X_0β_ (R = OH)	8.71 ± 0.66 nM	3.26 ± 0.32 nM	25.18 ± 0.47 nM
Doxorubicin (positive control)	9.61 ± 1.25 μM	2.54 ± 0.23 μM	0.70 ± 0.01 μM

**Table 12:** Structure and anti-glioblastoma activity of antimycins from the *Streptomyces antibioticus* H12-15 of marine origin against human glioblastoma cell line SF-268 [96].

Compound	Structure	IC_50,_ μg/mL
Neoantimycin A (R_1_ = CH(CH_3_)CH_2_CH_3_	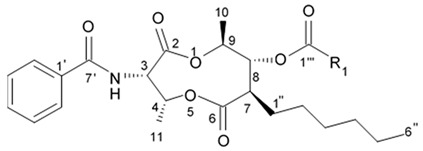	33.6
Neoantimycin B (R_1_ = CH(CH_3_)_2_		41.6
Antimycin A_1ab_ (a) (R_2_ = CH(CH_3_)CH_2_CH_3_), R_3_ = *n*Hex (b)(R_2_ = CHCH_2_(CH_3_)_2_, R_3_ = *n*Hex	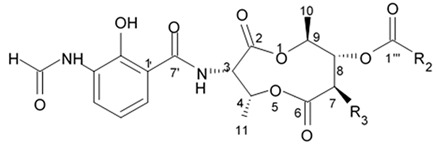	<1.6
Antimycin A_2a_ (R_2_ = CH(CH_3_)_2_, R_3_ = *n*Hex		<1.6
Antimycin A_9_ (R_2_ = CH_2_Ph) R_3_ = *n*Hex		<1.6
*Cis*-dichlorodiamine platinum (positive control)		41.0

**Table 13:** Structure and anti-glioma activity of the anthracycline analogue SZ-685C from mangrove endophytic fungus no. 1403 against glioma cell line LN-444 [97].

Compound	Structure	IC_50_ (μM)
SZ-685C	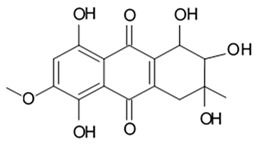	7.8

**Table 14:** Structure and inhibitory activity of bagremycins B and C from a mangrove-derived actinomycete, *Streptomyces* sp. Q22 against glioma cells lines [98].

Compound	Structure	IC_50_ (µM)			
		U87MG	U251	SHG44	C6
Bagremycin B	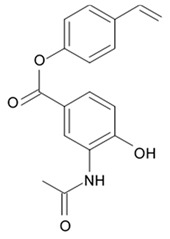	10.2 ± 0.5	9.7 ± 1.9	7.3 ± 0.8	13.3 ± 2.4
Bagremycin C	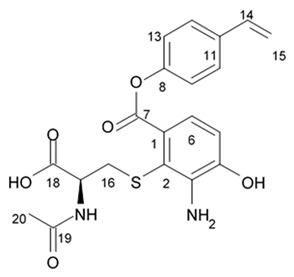	2.2 ± 0.1	4.3 ± 0.4	2.4 ± 0.2	6.4 ± 0.5
Doxorubicin		0.4 ± 0.0	3.3 ± 0.7	1.9 ± 0.0	0.5 ± 0.1

**Table 15:** Structure and cytotoxicity activity of the capoamycin-type antibiotics from marine *Streptomyces fradiae* PTZ0025 against glioma C6 cells [100].

Compound	Structure	IC_50_ (μM)
Fradimycin A	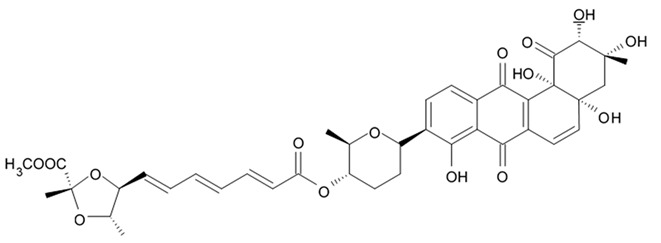	1.28 ± 0.37
Fradimycin B	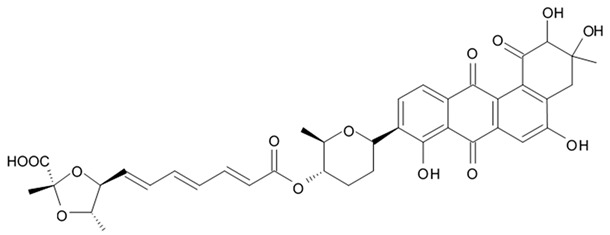	0.47 ± 0.09
MK844-mF10	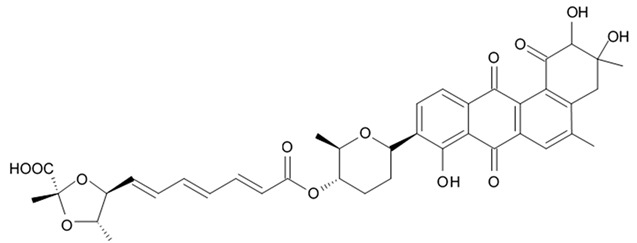	1.31 ± 0.32

**Table 16:** Structure and inhibitory activity of polyene-polyol macrolides from *Streptomyces* sp. ZQ4BG against glioma cells lines U251, U87MG, SHG44, and C6 [102].

Compound	Structure	IC_50_ (μM)
Flavofungin I	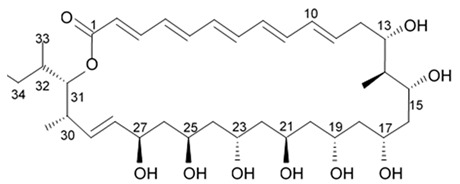	45.91–87.45
Flavofungin II	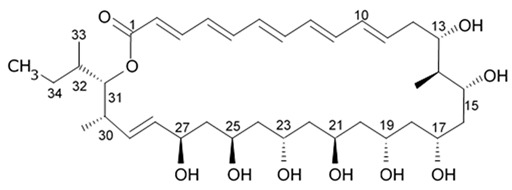	15.67–56.67
Spectinabilin	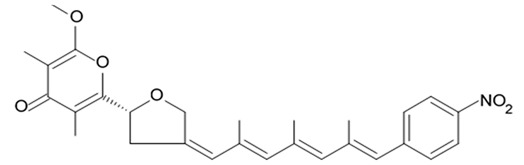	10.86–42.75

**Table 17:** Structure and cytotoxicity of asterosaponin 1 from the starfish *Culcita* *novaeguineae* on human glioblastoma U87MG cells [104].

Compound	Structure	IC_50_, μg/mL
Asterosaponin 1	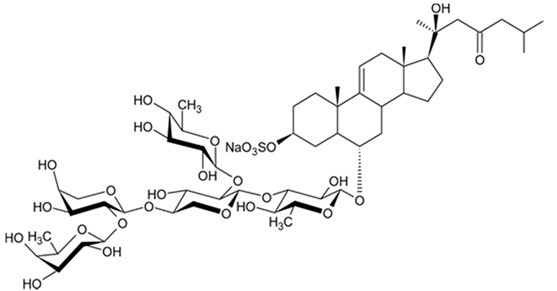	4.3

**Table 18:** Structure of asterosaponins from starfish *Culcita* *novaeguineae* and their inhibiting activity against human and mouse glioblastoma cell lines and human astrocytes [103].

Sample and Structure	IC_50_ (μM)					
	U87MG	U251MG	BT325	SHG44	C-6	Astrocytes
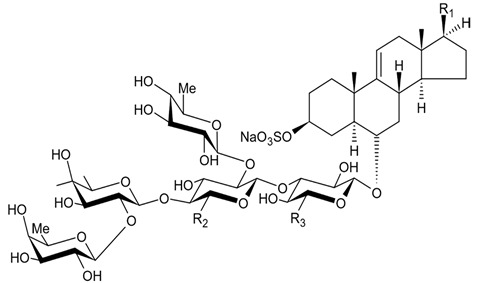						
	1		2		3	
	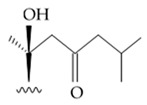		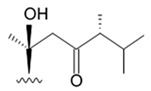		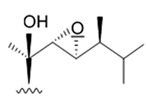	
Saponin 1	2.88 ± 0.31	2.24 ± 0.38	3.72 ± 0.85	3.86 ± 0.92	1.82 ± 0.09	>50
Saponin 2	2.10 ± 0.24	2.45 ± 0.40	2.66 ± 0.78	3.05 ± 0.60	1.40 ± 0.07	>50
Saponin 3	2.49 ± 0.28	3.05 ± 0.31	2.83 ± 0.86	3.22 ± 0.74	1.45 ± 0.10	>50
Saponin 4 (degrading the terminal *D*-fucose based on saponin 1)	1.22 ± 0.25	1.46 ± 0.22	1.88 ± 0.45	1.97 ± 0.51	0.59 ± 0.05	>50

**Table 19:** Structure of polyhydroxysteroidal glycosides from starfish *Culcita novaeguinea* and their cytotoxic activity against glioblastoma cell lines in vitro [105].

Compound and Structure		IC_50_, µM		
		U87	U251	SHG44
Culcinoside A	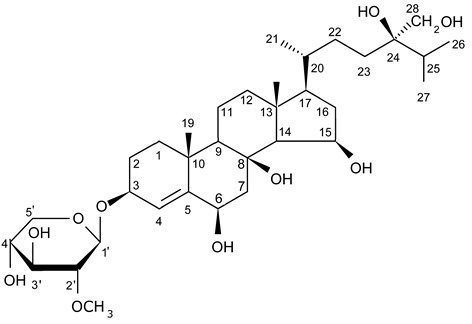	9.35 ± 0.46	11.28 ± 0.65	8.04 ± 0.32
Culcinoside B	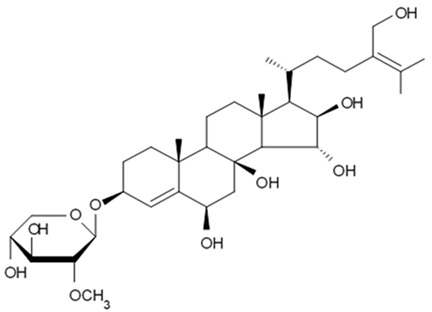	33.52 ± 1.23	40.76 ± 1.58	36.54 ± 1.44
Culcinoside C	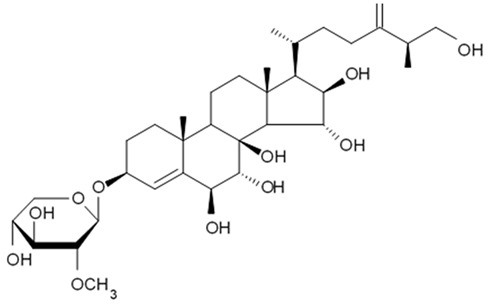	26.33 ± 1.16	22.66 ± 1.28	35.26 ± 1.51
Culcinoside D	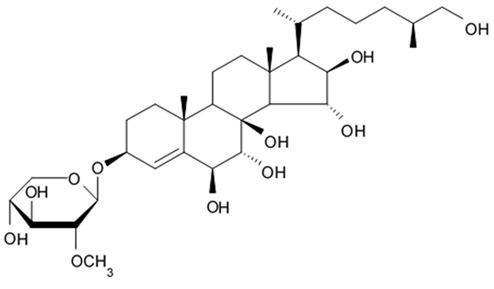	43.25 ± 1.73	28.93 ± 1.83	26.22 ± 1.64
Doxorubicin		0.33 ± 0.02	0.24 ± 0.01	0.15 ± 0.01

**Table 20:** Structure of anthenoside A from the starfish *Anthenea chinensis* and their cytotoxic activity against glioblastoma U87MG cells [106].

Compound	Structure	IC_50_ (μg/mL)
Anthenoside A	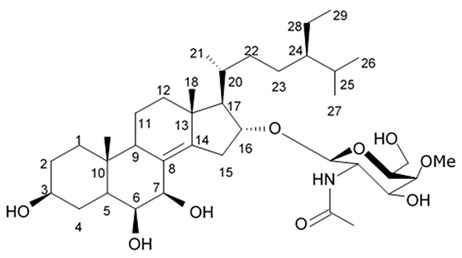	1.9

**Table 21:** Structure of triterpene glycosides from *Pentacta quadrangularis* and their cytotoxicity against glioblastoma U87MG cell lines in vitro [109].

Compound	Structure	IC_50_, µM
	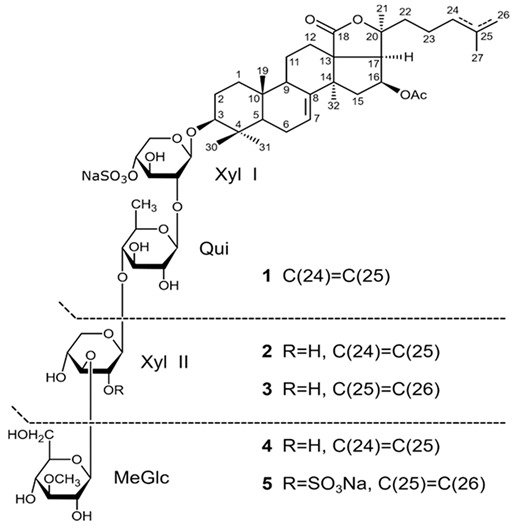	
**1**—Pentactaside III		3.95 ± 0.53
**2**—Pentactaside I		3.57 ± 0.35
**3**—Pentactaside II		3.44 ± 0.40
**4**—Philinopside A		2.74 ± 0.32
**5**—Philinopside B		1.90 ± 0.21
Hydroxycamptothecine (positive control)		1.39 ± 0.17

**Table 22:** Structure and anti-glioma activity of sulfated saponins from sea cucumber *Holothuria moebii* [113].

Compound and Structure	IC_50_, μM			
	U87-MG	U251	SHG-44	C6
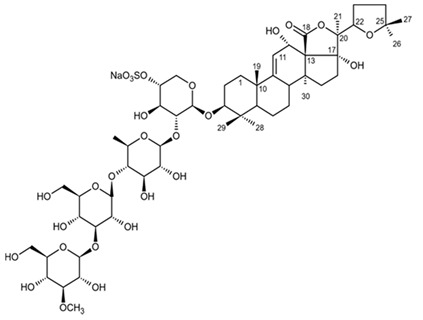	4.03 ± 0.55	3.76 ± 0.08	3.68 ± 0.16	0.99 ± 0.17
Holothurin A				
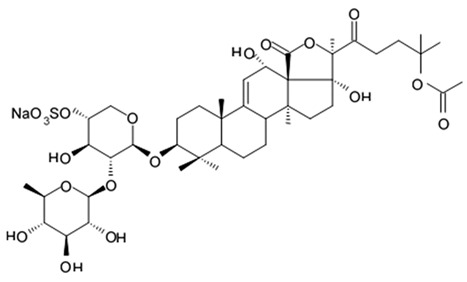	3.00 ± 0.13	8.64 ± 2.30	1.39 ± 0.83	2.86 ± 0.23
Holothurin B				
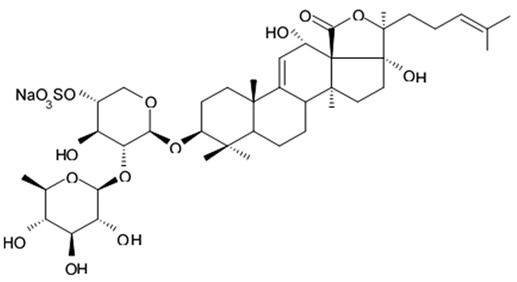	2.72 ± 0.04	6.10 ± 0.71	1.99 ± 0.18	2.09 ± 0.72
24-dehydroechinoside B				
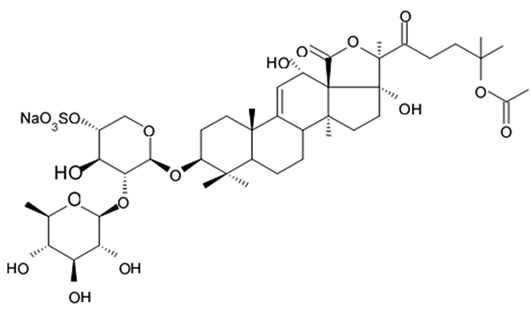	3.81 ± 0.07	4.39 ± 0.52	2.80 ± 0.69	1.22 ± 0.10
New triterpenoid saponin (see text)				
Temozolomide	>100.00	>100.00	85.8 ± 4.10	69.5 ± 6.10

**Table 23:** Structure and anti-glioma activities of plakortide O isolated from the marine sponge *Plakortis halichondroides* [123].

Compound	Structure	IC_50,_ μmol/L	
		U87-MG	U373-MG
Plakortide O	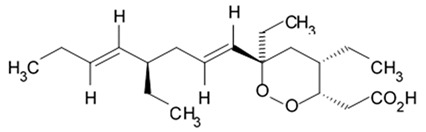	4.0	4.0

**Table 24:** Structure and anti-glioma activities of diterpenes from the gorgonian octocoral *Eunicea succinea* [123].

Compound	Structure	IC_50_ (μmol/L)	
		U87-MG	U373-MG
Eupalmerin acetate	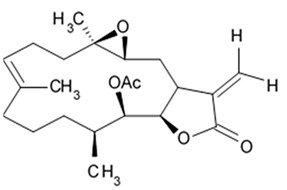	5.1	6.9
Isoeupalmerin acetate	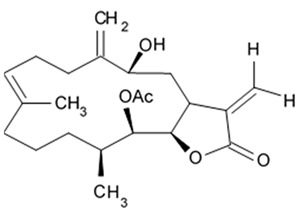	6.7	16.0
3′-O-acetyl-pseudopterosin U	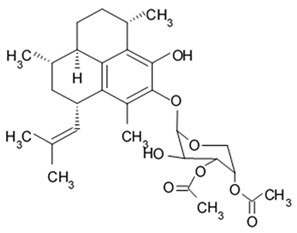	14.0	37.0
3-Epi-14-deoxycrassin	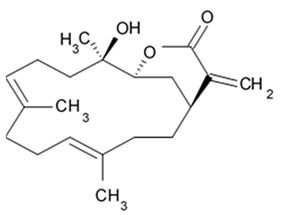	25.8	64.2
Asperdicin	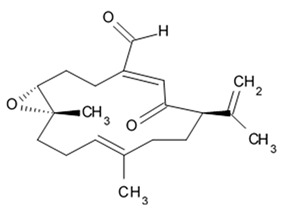	26.3	56.0

**Table 25:** In vitro anti-glioma activity of stellettin B from marine sponge *Jaspis stellifera* against human glioblastoma cell line SF295 [124].

Compound	Structure	IC_50_
Stellettin B	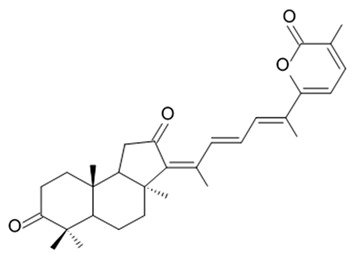	0.01 μM
IG_50_ (IC_50_)		>100

**Table 26:** Structure and cytotoxicity of the coibamide A from the marine cyanobacterium *Leptolyngbya* sp. against glioma cells [134].

Compound	Structure	EC_50_ (nM)	
		U87-MG	SF-295
Coibamide A	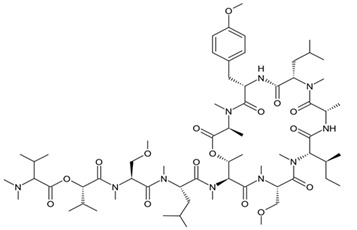	28.8 ± 8.4	96.2 ± 23

**Table 27:** Structure and anti-glioma activity of cyclodepsipeptides from actinomycete *Streptomyces* sp. P11-23B [137].

Compound and Structure	IC_50_ (μM)			
	U251	U87-MG	SHG-44	C6
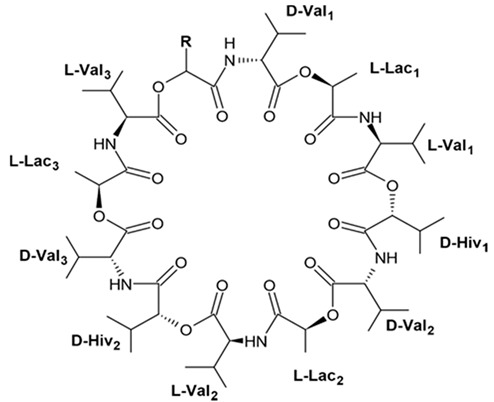				
Valinomycin, **R** = -(CH)(CH_3_)_2_ (D-Hiv_3_)	7.6 ± 0.7 nM	30.0 ± 2.8 nM	21.0 ± 2.9 nM	24.0 ± 2.0 nM
Streptodepsipeptide P11A, **R** = -CH_2_CH_3_ (D-Hba)	0.4 ± 0.0	0.4 ± 0.0	0.3 ± 0.0	0.3 ± 0.0
Streptodepsipeptide P11B, **R** = -CH_3_ (L-Lac_4_)	0.5 ± 0.0	0.2 ± 0.0	1.4 ± 0.2	0.1 ± 0.0
Doxorubicin	3.3 ± 0.7	0.4 ± 0.0	1.9 ± 0.0	0.5 ± 0.1

**Table 28:** Cytotoxic activity of ergosterols from the marine actinomycete *Streptomyces anandii* H41-59 against human glioblastoma SF-268 cells [141].

Compound	IC_50_ (µg/mL)	Compound	IC_50_ (µg/mL)
ananstrep A	>50	**8**	15.5
ananstrep B	>50	**9**	>50
ananstrep C	13.0	**10**	27.8
4 *	>50	**11**	25.1
**5**	>50	**12**	>50
**6**	>50	**13**	>50
**7**	>50	*cis*-dichlorodiamine platinum	41.0

* see text.

**Table 29:** Anti-glioma activity of polyoxygenated 24,28-epoxyergosterols from the sea anemone *Anthopleura midori* [142].

Compound	IC_50_ (μM)		Compound	IC_50_ (μM)	
	C6	U251		C6	U251
1 *	2.41 ± 0.52	40.94 ± 1.06	**7**	38.38 ± 3.25	80.45 ± 4.07
**2**	18.59 ± 0.32	64.26 ± 3.75	**8**	72.14 ± 2.70	NA
**3**	43.60 ± 0.93	NA **	**9**	NA	NA
**4**	NA	NA	**10**	NA	NA
**5**	10.58 ± 0.57	41.91 ± 2.99	**11**	38.38 ± 3.25	46.08 ± 1.65
**6**	NA	NA	Temozolomide	69.58 ± 6.10	NA

* see text; ** NA: no inhibitory activity at concentration of 100 μM.

**Table 30:** Antiglioma activity of anthraquinones from *Streptomyces* sp. ZZ406 [143].

Compound and Structure	IC_50_ (μM)			CC_50_ (μM)
	U251	U87MG	SHG44	Human Astrocytes
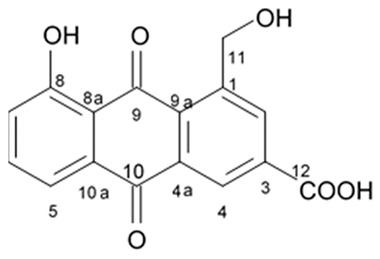	5.7 ± 0.3	4.7 ± 0.2	8.1 ± 0.4	>100
1-hydroxymethyl-8-hydroxy-anthraquinone-3-carboxylic acid				
(CC_50_/IC_50_)	>17.5	>21.3	>12.3	
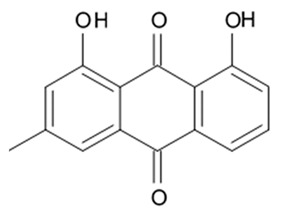	1.2 ± 0.1	0.1	3.0 ± 0.2	No testing
Chrysophanol				
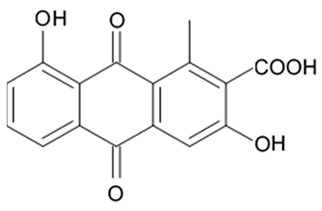	13.0 ± 0.6	10.4 ± 0.5	36.3 ± 0.2	No testing
3,8-dihydroxy-1-methyl-anthraquinone-2-carboxylic acid				
Doxorubicin (CC_50_/IC_50_)	9.6 ± 1.3 (0.9)	1.9 ± 0.4 (4.6)	2.5 ± 1.1 (3.5)	8.7 ± 1.2

**Table 31:** Structure and anti-glioma activities of the polycyclic quinones from marine *Streptomyces* sp. 182SMLY [145].

Compound and Structure	IC_50_ (μM, 72 h)			
	U251	U87-MG	SHG-44	C6
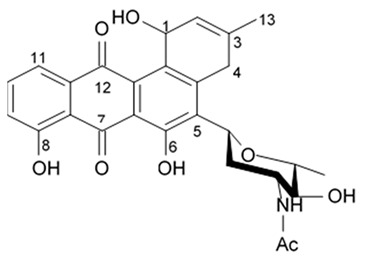	0.7 ± 0.2 (35)	1.4 ± 0.1 (18)	3.9 ± 0.4 (6.4)	0.5 ± 0.1 (53)
N-acetyl-N-demethylmayamycin (Racios of IC_50 astrocytes_/IC_50 glioma cells_)				
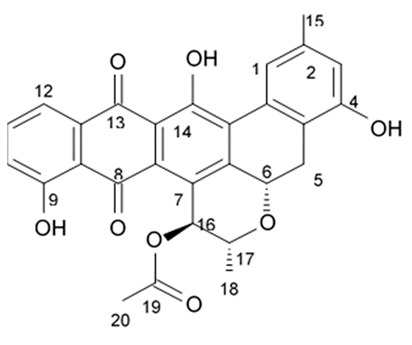	3.3 ± 0.3 (>31)	4.6 ± 0.3 (>22)	6.5 ± 1.1 (>16)	7.3 ± 1.4 (>14)
Streptoanthraquinone A (Racios of IC_50 astrocytes_/IC_50 glioma cells_)				
Doxorubicin	6.7 ± 1.1	0.9 ± 0.1	9.0 ± 0.8	1.0 ± 0.1

**Table 32:** Structure and cytotoxicity of the anthraquinone derivatives from the sea lilies *Comanthus* sp. against C6 glioma cells [147].

Compound	Structure	IC_50_, μM
1′-Deoxyrhodoptilometrin	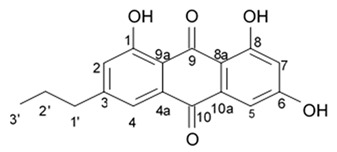	23.2
(*S*)-(−)-rhodoptilometrin	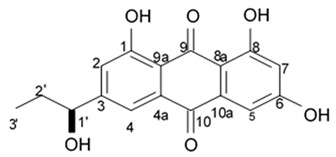	30.0

**Table 33:** Cytotoxic activity of glycolipid fractions containing three glucosylceramides from starfish *Narcissia canariensis* against human astrocytoma cells [154].

Compounds and Structure	IC_50,_ (μM)
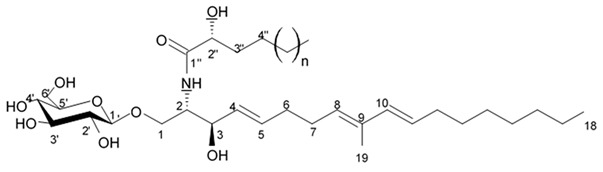	
Ophidiacerebroside B (*n* = 16) Ophidiacerebroside C (*n* = 17), 63% Ophidiacerebroside D (*n* = 18)	34.6 ± 5.1



**Table 34:** Cytotoxic activity of glycolipid fractions containing three glycosphingolipids from marine sponge *Axinyssa djiferi* against human astrocytoma cells [155].

Compounds and Structure	IC_50_, μM
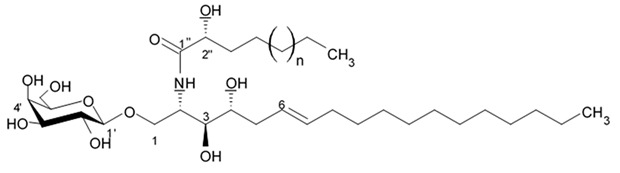	
Axidjiferoside A (*n* = 18), 60.8% Axidjiferoside B (*n* = 17), 22.4% Axidjiferoside C (*n* = 19), 16.8%	
	>60 μM


**Table 35:** In vitro growth inhibitory activity of ethyl acetate fraction containing two sphingosines from marine sponge *Haliclona tubifera* against human glioma U87 and human neuroblastoma SH-SY5Y cell lines [156].

Compounds	Structure	IC_50_, μg/mL	
		U87	SH-SY5Y
Halisphingosine A	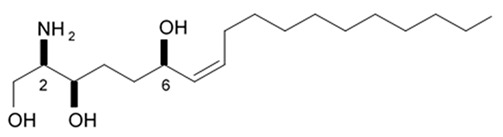	12.47 ± 1.28	16.72 ± 1.24
Halisphingosine B	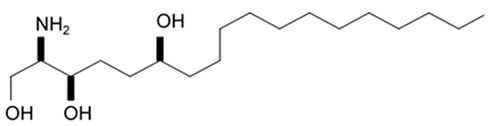		

**Table 36:** Structure and anti-glioma activity of psammaplin A against glioblastoma U373MG cells [166].

Compound	Structure	IC_50_
Psammaplin A	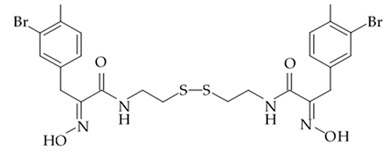	5 μg/mL

**Table 37:** Chemical structure and antiglioblastoma U251 activity of the xyloketal B from mangrove fungus *Xylaria* sp. (No. 2508) [182].

Compound	Structure	IC_50_ (μM)
Xyloketal B	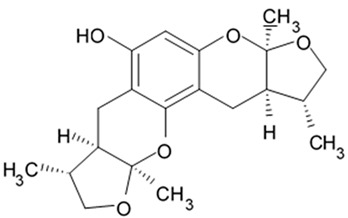	287.1 ± 1.0

**Table 38:** Anti-glioma activity pheophorbide *a* from a red seaweed *Grateloupia elliptica* in U87MGcell line, in vitro [184].

Compound	Structure	IC_50_ (μg/mL)	
		U87MG	HUVEC
Pheophorbide *a*	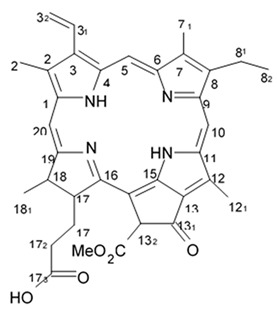	2.8 ± 1.0	>50
Paclitaxel (positive control)		3.2 ± 1.2	0.3 ± 1.0

**Table 39:** Structure and antiproliferative activity of synthetic phorboxazole A and its analogues against human glioblastoma cell line U373 [187].

Compound	Structure	IC_50_ (nM)
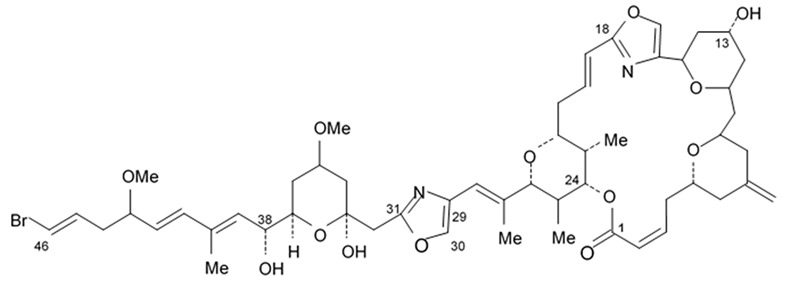		6.7
Phorboxazole A		
45,46-Dehydrobromo-phorboxazole A		27.4
33-O-Methyl-phorboxazole A		29.2
32-Methyl-phorboxolide A		>2000
31-Methyl-phorboxylate		>2000
29-Phorboxamide A		>2000
C1–C38 of phorboxazole A		>2000
18-Methyl-phorboxylate		>2000

**Table 40:** Antiproliferative activity of the phloroglucinol derivative against glioma cells, U251, U87, and C6 [194].

Compound and Structure	IC_50_ (µM)			
	U251	U87	C6	Human Astrocytes
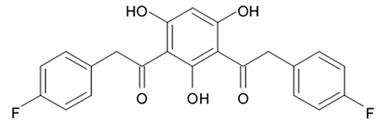	<1 µM	<3 µM	<10 µM	>30 µM
BFP				
Staurosporine (positive control)	≈30 nM			n.d.

**Table 41:** Anti-glioma activity of the hydratoperidinin from the sea anemone *Anthopleura midori* [111].

Compound	Structure	IC_50_, μM	
		C6	U251
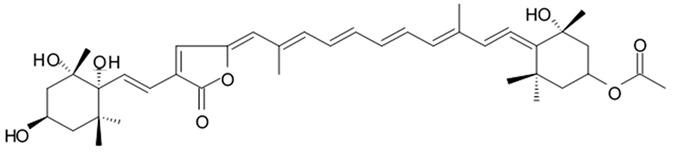			43.72 ± 1.73
Hydratoperidinin 18.70 ± 0.81			
Temozolomide		69.58 ± 6.10	NA *

* NA: no inhibitory activity at concentration of 100 μM.

**Table 42:** Taxonomy of marine organisms containing bioactive compounds with experimentally confirmed anti-glioma activity (classification is presented in accordance with World Register of Marine Species).

Species */Strain	Anti-Glioma Compound	Reference
Kingdom Bacteria		
Phylum Actinobacteria		
Class Actinobacteria		
Order Actinomycetales		
Family Streptomycetaceae		
*Streptomyces* sp. ZZ406	Anthraquinones: 1-hydroxymethyl-8-hydroxy-anthraquinone-3-carboxylic acid, chrysophanol, 3,8-dihydroxy-1-methyl-anthraquinone-2-carboxylic acid. Chromon phaeochromycin I.	[143]
*Streptomyces* sp. P11-23B	Cyclodepsipeptides: valinomycin, streptodepsipeptide P11A, streptodepsipeptide P11B.	[137]
*Streptomyces* sp. ZQ4BG	Antibiotics macrolides: flavofungin I, flavofungin II, spectinabilin.	[102]
*Streptomyces* sp. Q22	Bagremycin antibiotics: bagremycins B, C.	[98]
*Streptomyces* sp. 182SMLY	Polycyclic quinones: N-acetyl-N-demethylmayamycin, streptoanthraquinone A.	[145]
*Streptomyces* sp. ZZ338	Actinomycin antibiotics: actinomycin D, actinomycin V, actinomycin X_0β_.	[94]
*Streptomyces fradiae* PTZ0025	Capoamycin-type antibiotics: fradimycin A, fradimycin B, MK844-mF10.	[100]
*Streptomyces antibioticus* H12-15	Antimycin antibiotics: neoantimycins A, B, antimycins A_1ab_, A_2a_, A_9_.	[96]
*Streptomyces anandii* H41-59	New ergosterols: ananstreps A, B, C, ten known ergosterols.	[141]
Phylum Firmicutes		
Class Bacili		
Order Bacillales		
Family Bacillaceae		
*Bacillus* sp. FS8D	Alkaloid pseurotin A	[83]
Phylum Cyanobacteria		
Class Cyanophyceae		
Order Synechococcales		
Family Leptolyngbyaceae		
*Leptolyngbya* sp.	Cyclopeptide coibamide A	[134]
Kingdom Fungi		
Phylum (Division) Ascomycota		
Class Sordariomycetes		
Order Xylariales		
Family Xylariaceae		
*Xylaria* sp. (No. 2508)	Xyloketal B	[182]
*Halorosellinia* sp. (No. 1403)	Anthracycline antibiotic SZ-685C	[97]
Class Eurotiomycetes		
Order Eurotiales		
Family Trichocomaceae		
*Penicillium* sp. ZZ380	Alkaloids: pyrrospirones C–J, penicipyrroether A	[80,81]
Order: incertae sedis		
*Trichobotrys effuse* 4729	Alkaloid trichobamide A	[82]
Kingdom Plantae		
Phylum (Division) Rhodophyta		
Class Florideophyceae		
Order Ceramiales		
Family Rhodomelaceae		
*Laurencia tristicha*	Sesquiterpene aplysin	[118]
Order Halymeniales		
Family Halymeniaceae		
*Grateloupia elliptica*	Pheophorbide *a*	[184]
Red algae	Phlorotannin eckol	[190]
Kingdom Chromista		
Phylum Ochrophyta		
Class Phaeophyceae		
Brown algae	Phlorotannin eckol	[190]
Microalgae and macroalgae	Carotenoid fucoxanthin	[200]
Kingdom Animalia		
Phylum Porifera		
Class Demospongiae		
Order Trachycladida		
Family Trachycladidae		
*Trachycladus laevispirulifer*	Nucleosides: trachycladines A, B	[149]
Order Suberitida		
Family Halichondriidae		
*Axinyssa djiferi*	Glycosphingolipids: axidjiferosides A, B, C	[155]
Order Haplosclerida		
Family Chalinidae		
*Haliclona (Reniera) tubifera*	Sphingosins: halisphingosines A, B	[156]
Family Petrosiidae		
*Xestospongia* sp.	Alkaloid renieramycin M	[72]
*Neopetrosia* cf. *chaliniformis* (*Neopetrosia* cf. *exigua*)	Alkaloids: papuamine, haliclonadiamine, neopetrocyclamine A, neopetrocyclamine B	[86]
Order Verongiida		
Family Pseudoceratinidae		
*Pseudoceratina* (*Psammaplysilla*) sp., *Pseudoceratina purpurea*	Psammaplins: A, C	[164,166]
Order Poecilosclerida		
Family Hymedesmiidae		
*Phorbas* sp.	Phorboxazoles: A, B	[187]
Order Dictyoceratida		
Family Thorectidae		
*Fascaplysinopsis* sp.	Alkaloids: fascaplisin, 7-phenylfascaplisin, 3-chlorofascaplisin, 3-bromofascaplisin, 10-bromofascaplysin	[64]
Order Tetractinellida		
Family Ancorinidae		
*Jaspis stellifera*	Terpene stellettin B	[124]
Class Homoscleromorpha		
Order Homosclerophorida		
Family Plakinidae		
*Plakortis halichondroides*	Plakortide O	[123]
Phylum Cnidaria		
Class Anthozoa		
Order Actiniaria		
Family Actiniidae		
*Anthopleura anjunae* (*Anthopleura midori*)	7 sterols, Carotenoid hydratoperidinin	[142]
Order Alcyonacea		
Family Plexauridae		
*Eunicea succinea*	Terpenes: eupalmerin acetate, isoeupalmerin acetate, 3′-o-acetyl-pseudopterosin U, 3-epi-14-deoxycrassin, asperdicin, pseudoplexauric acid methyl ester, 2-deoxyasperdiol acetate	[123]
Phylum Echinodermata		
Class Crinoidea		
Order Comatulida		
Family Comatulidae		
*Comanthus AH Clark* (*Comanthus*) sp.	Anthraquinone derivatives: 1′-deoxyrhodoptilometrin, (*S*)-(−)-rhodoptilometrin	[147]
Class Asteroidea		
Order Valvatida		
Family Oreasteridae		
*Culcita* *novaeguineae*	Steroidal saponin asterosaponin 1	[104]
	4 steroidal saponins;	[103]
	Polyhydroxysteroidal glycosides: culcinosides A, B, C, D	[105]
*Anthenea pentagonula* (*Anthenea chinensis*)	Polyhydroxysteroidal glycoside anthenoside A	[106]
*Pentaceraster chinensis*	2 steroidal glycosides	[108]
Family Ophidiasteridae		
*Narcissia canariensis*	Glycosphingolipids: ophidiacerebroside B, C, D	[154]
Order Paxillosida		
Family Ctenodiscidae		
*Ctenodiscus crispatus*	Steroid: (25S)-5α-cholestane-3β,5,6β,15α,16β,26-hexaol	[139]
Class Holothuroidea		
Order Dendrochirotida		
Family Cucumariidae		
*Pentacta* sp.	Triterpene glycosides: pentactasides I, II, III, philinopsides A, B	[109]
Order Holothuriida		
Family Holothuriidae		
*Bohadschia marmorata*	3 triterpene glycosides	[103]
*Holothuria* (*Stauropora*) *fuscocinerea*	Triterpene glycoside fuscocineroside A	[110]
*Holoturia* (*Microthele*) *fuscopunctata* (*Holoturia axiologa*)	Triterpene glycoside pervicoside D	[103]
*Holothuria* (Selenkothuria) *moebii*	Triterpenoid saponins: holothurin A, holothurinan B, 24-dehydroechinoside B	[113]
Phylum Arthropoda		
Subphylum Chelicerata		
Class Merostomata		
Order Xiphosurida		
Family Limulidae		
*Tachypleus tridentatus*	Tachyplesin I	[130,131]
Phylum Mollusca		
Class Gastropoda		
Order Aplysiida		
Family Aplysiidae		
*Aplysia kurodai*	Terpene aplysin	[119,120]
Phylum Chordata		
Subphylum Tunicata		
Class Ascidiacea		
Order Stolidobranchia		
Family Styelidae		
*Polyandrocarpa zorritensis*	Alkaloids: zorrimidazolone, 3-indolylglyoxylic acid, 3-indolylglyoxylic acid methyl ester	[39]
Dendrodoa grossularia	Alkaloids α-carbolines	[69]
Polycarpa aurata	Alkaloids α-carbolines	[69]
Order Phlebobranchia		
Family Perophoridae		
*Ecteinascidia turbinata*	Alkaloids ecteinascidin-770, 2′-N-4″-pyridinecarbonyl derivative of ET-770	[72]
Order Aplousobranchia		
Family Didemnidae		
*Didemnum granulatum*	Alkaloids: granulatimide, isogranulatimide, 2 amino analogs.	[89]
*Eudistoma* cf. glaucum (*Eudistoma* cf. *rigida*)	Alkaloids: 7 synthetic analogues of rigidins	[79]

* Taxons in parentheses are synonyms.

## Data Availability

All the data provided in the review article.
